# Plastome evolution in Aurantioideae and plastome-based classification of *Citrus*

**DOI:** 10.1371/journal.pone.0349882

**Published:** 2026-10-07

**Authors:** Eranga Pawani Witharana, Myat Htoo San, Nadeeka U. Jayawardana, Nobuhiro Kotoda, Masashi Yamamoto, Yukio Nagano

**Affiliations:** 1 Faculty of Agriculture, University of Peradeniya, Peradeniya, Sri Lanka; 2 Analytical Research Center for Experimental Sciences, Saga University, Saga, Japan; 3 Graduate School of Advanced Health Science, Saga University, Saga, Japan; 4 The United Graduate School of Agricultural Sciences, Kagoshima University, Kagoshima, Japan; 5 Faculty of Agriculture, Saga University, Saga, Japan; 6 Applied BioSciences, Macquarie University, Sydney, New South Wales, Australia; 7 Faculty of Agriculture, Kagoshima University, Kagoshima, Japan; 8 Vishnu Institute of Pharmaceutical Education and Research, Narsapur, Telangana, India; 9 Shri Vishnu College of Pharmacy, Bhimavaram, Andhra Pradesh, India; Institute for Biological Research, University of Belgrade, SERBIA

## Abstract

Plastomes are widely used in plant systematics and also retain signals of structural evolution. In this study, we investigated plastome evolution in the subfamily Aurantioideae of Rutaceae, focusing on structural variation at the LSC–IR boundary regions, putative pseudogenization of plastid genes, plastome-based classification of *Citrus*, and divergence-time patterns. Comparative analyses revealed substantial structural variation around the *rpl22* and *rps19* regions at the LSC–IR boundaries and repeated pseudogenization of plastid genes such as *rpl22*, *ycf68*, and *orf56*. Additional lineage-specific sequence disruptions were detected in *cemA*, *ycf15*, *rps4*, and *ycf1*. Phylogenetic analyses of complete plastomes from 56 *Citrus* accessions resolved eight major plastome clusters within *Citrus*. Divergence-time analysis indicated two comparable Asia–Africa splits within Aurantioideae, whereas the divergence between Australian and Asian *Citrus* lineages was more recent. These results show that plastome evolution in Aurantioideae combines phylogenetically informative sequence variation with recurrent local structural change and provides useful information for classification and evolutionary interpretation in this economically important plant group.

## Introduction

Plastomes have become widely used resources in plant systematics because they can be assembled efficiently from genome-skimming data and provide abundant phylogenetic information for tracing plastome lineages [1,2]. Their relatively conserved organization, uniparental inheritance, and moderate genome size make plastomes particularly useful for resolving relationships among closely related taxa [1]. At the same time, complete plastome sequences offer more than substitution-based phylogenetic signal alone and provide additional insight into genome evolution, including structural rearrangements [3].

The subfamily Aurantioideae of the family Rutaceae provides an excellent system for exploring such plastome evolution. This group includes *Citrus* and its relatives, which are of major agricultural and economic importance [4,5]. Some members of Aurantioideae, including bael (*Aegle marmelos*), wood apple (*Limonia acidissima*), and curry tree (*Murraya koenigii*), have long histories of cultivation and human use, making their evolutionary relationships relevant not only to systematics but also to germplasm management, breeding, and the interpretation of crop origins [6–8]. Within this subfamily, *Citrus* is especially challenging because its taxonomy has been complicated by extensive morphological variation and a long history of hybridization [5,7].

Previous studies have shown that phylogenetic relationships in Aurantioideae are not always consistent across genomic compartments [4,9]. Trees based on plastomes may differ from those based on nuclear data, reflecting differences in inheritance and evolutionary processes. Whereas plastome DNA traces a single lineage, nuclear genomes may retain signals of incomplete lineage sorting, introgression, and hybrid origin [9]. Despite these limitations, plastome data remain highly informative because they provide a coherent framework for tracing maternal lineages, and complete plastome sequences are particularly useful for examining diversity within taxonomically complex groups such as *Citrus* [10,11].

In addition to phylogenetic reconstruction, plastome data are valuable for understanding structural aspects of genome evolution. Although plastomes in land plants are often regarded as structurally conserved, comparative studies have revealed lineage-specific rearrangements and variation around junctions between genomic regions, including the boundaries between the large single-copy region (LSC) and the inverted repeat regions (IRs) [3]. These architectural features can complement sequence-based phylogenies and may help clarify patterns that are otherwise difficult to interpret. In Aurantioideae, such information is especially relevant because both deep divergences among major lineages and more recent differentiation within *Citrus* are important for understanding the evolutionary history of the group [11,12].

Gene pseudogenization represents another aspect of plastome evolution that can occur within an otherwise conserved genomic framework [13]. Pseudogenes are gene-derived sequences that have lost their original coding capacity; in comparative plastome analyses, putative pseudogenes are generally identified by disruptions such as frameshift mutations, premature termination codons, loss of recognizable start codons, or substantial deletions [14]. Such changes have been documented across diverse plant lineages and may accumulate under relaxed selective constraints or, in some cases, following functional replacement by a nuclear-encoded copy [14,15]. Pseudogenization can also accompany structural changes, including shifts in IR boundaries that generate truncated gene copies, linking variation in gene integrity with plastome architecture. However, sequence disruption alone does not establish the underlying evolutionary mechanism or demonstrate loss of biological function, particularly for putative coding regions whose functional status remains uncertain [16]. In Aurantioideae, examining these disruptions alongside boundary variation and plastome phylogeny provides an opportunity to distinguish shared patterns from lineage-specific changes within a taxonomically complex group. Accordingly, comparative assessment of gene integrity can complement structural and phylogenetic analyses without implying experimental validation of gene function.

Here, we investigate plastome conservation and localized variation in Aurantioideae within a phylogenetic and temporal framework. This study builds on previously generated sequencing resources and plastome analyses presented in our earlier bioRxiv preprint [17], complementing our peer-reviewed studies of plastome–nuclear phylogenetic discordance [9] and a large plastome inversion [3]. We first characterize plastome organization and gene content across the subfamily, then examine structural variation at the LSC–IR boundaries and recurrent patterns of putative gene pseudogenization. We also investigate additional lineage-specific coding-sequence disruptions affecting *cemA*, *ycf15*, *rps4*, and *ycf1*. Subsequently, we use whole-plastome phylogenetic analysis to assess maternal-lineage relationships within *Citrus* and estimate divergence times among major Aurantioideae lineages, with particular attention to Asia–Africa disjunctions. Together, these analyses reveal broadly conserved plastome organization alongside localized structural and coding-sequence variation, while providing a phylogenetic and temporal context for understanding plastome diversification within the subfamily.

## Materials and methods

### Plant materials, plastome dataset, assembly, and annotation

The present study investigated plastome evolution within the subfamily Aurantioideae of the family Rutaceae. A total of 103 plastome accessions were analyzed, comprising 88 Aurantioideae accessions and 15 outgroup taxa. The dataset included 29 accessions for which sequencing data had been generated in our previous studies [3,9], 15 accessions represented by publicly available raw sequencing datasets, and 59 accessions represented by publicly available complete plastome sequences. Thus, no new sequencing data were generated specifically for the present study. A complete list of the taxa, data sources, and corresponding sequence accession numbers is provided in S1 Table.

Different subsets of this dataset were used for specific analyses. Pseudogenization patterns were examined in 45 Aurantioideae taxa together with 14 additional Rutaceae accessions for comparative purposes. Plastome-based classification within *Citrus* was conducted using 56 *Citrus* accessions. Divergence-time estimation focused primarily on Aurantioideae and included four additional Rutaceae accessions, with *Ailanthus altissima* designated as the outgroup.

For the 29 accessions generated in our previous studies [3,9], DNA originated from the same extraction batches used in those studies. DNA quality was evaluated by electrophoresis on a 1% agarose gel, and DNA concentration was determined using a DNA BR Assay Kit (Thermo Fisher Scientific, USA). Sequencing libraries were prepared from total genomic DNA by Novogene (Singapore) using the NEBNext Ultra DNA Library Prep Kit for Illumina (New England Biolabs, USA) and sequenced on an Illumina NovaSeq 6000 platform to generate 150-bp paired-end reads. In addition, raw sequencing data for 15 accessions were obtained from public databases. Accession numbers and data sources for all raw sequencing datasets are provided in S1 Table. Raw reads used for plastome assembly were preprocessed using fastp v0.23.2 with default settings [18]. Summary statistics for the filtered reads, including the number of read pairs, total filtered bases, average read length, Q20 and Q30 percentages, and GC content, were calculated using a custom Python script (S2 Table).

De novo plastome assembly and annotation were performed following the procedures described in our previous study [9]. Plastomes were assembled from the 44 raw-read datasets using GetOrganelle v1.7.5 [19] with the parameters -R 15, -k 21,45,65,85,105, and -F embplant_pt. Assembly quality was evaluated by mapping the quality-filtered reads back to the corresponding assembled plastomes using Bowtie2 v2.4.4 [20]. The resulting alignments were processed using SAMtools v1.14 [21] and visually inspected in Integrative Genomics Viewer (IGV) to assess read coverage and assembly consistency. Qualimap v2.1.1 [22] was used to obtain quantitative mapping statistics, including the percentage of reads mapped to the assembled plastome, mean sequencing coverage, and mean mapping quality (S2 Table).

Plastome annotation was performed using GeSeq v2.03 [23], with protein-coding sequences predicted using Chloë v0.1.0, tRNA genes identified using tRNAscan-SE v2.0.7 [24], and rRNA genes identified using HMMER. Following automated annotation, all plastome annotations were manually inspected and corrected where necessary to ensure annotation accuracy and consistency. Final circular plastome maps were generated using OGDRAW v1.3.1 [25].

Accession numbers for the source raw sequencing reads and previously deposited plastome sequences, together with the corresponding data sources and information on plant materials processed by our group, are provided in S1 Table. The 15 plastomes assembled de novo from additional public raw-read datasets and the re-annotated plastome files derived from 59 public complete plastome sequences, manually curated where necessary, are available in the Supplementary Dataset deposited in Zenodo (https://doi.org/10.5281/zenodo.22743534).

### Comparative plastome sequence and LSC–IR boundary analyses

To assess sequence conservation and divergence among representative Aurantioideae plastomes, whole-plastome sequence comparisons were performed using mVISTA [26] with the Shuffle-LAGAN alignment mode [27], with the *Citrus sinensis* plastome used as the reference. Representative taxa included *Aegle marmelos*, *Atalantia bilocularis*, *A. ceylanica*, *A. roxburghiana*, *Citropsis gabunensis*, *Clausena anisata*, *Citrus glauca*, *Limonia acidissima*, *Glycosmis citrifolia*, *Naringi crenulata*, *Murraya caloxylon*, *Micromelum minutum*, *Murraya koenigii*, *Murraya paniculata*, *Paramignya lobata*, *Swinglea glutinosa*, and *Triphasia trifolia*. The resulting alignments were examined to evaluate patterns of sequence conservation and divergence across the plastomes.

To further investigate structural variation at plastome junctions, boundary regions were manually examined and compared among representative Aurantioideae plastomes, with particular attention to the junctions between the LSC and the IRs. The analysis focused especially on the LSC–IR boundary regions containing *rpl22* and *rps19*. Structural differences, including gene fragmentation, pseudogenization, and local gene loss, were identified manually by comparing gene order, gene orientation, and junction positions among the annotated plastomes.

### Whole-plastome alignment and phylogenetic analyses

Whole-plastome alignment and phylogenetic analyses were performed as in the previous study [9]. Complete plastome sequences were aligned using HomBlocks (v1.0) [28] with default settings. The best-fit evolutionary model was identified using ModelTest-NG (v0.1.7) [29]. Maximum-likelihood (ML) trees were constructed using RAxML (v8.2.12) [30] with 1,000 bootstrap replicates. Bayesian inference (BI) trees were generated using BEAST (v1.10.4) [31] with Markov chain Monte Carlo sampling for 10,000,000 generations, sampling every 10,000 generations and discarding the first 10% as burn-in. ML and BI trees were visualized using Dendroscope (v3.8.8) and FigTree (v1.4.4), respectively. In the present study, this framework was applied to the *Citrus* plastome dataset and the Aurantioideae-centered datasets used for comparative analyses.

### Identification and phylogenetic analysis of pseudogenes

Protein sequences were extracted from annotation files using genbank_to (v0.42) [32]. Orthologous protein groups were identified using OrthoFinder (v2.5.4) [33], and amino acid sequences were aligned using MAFFT (v7.515) [34]. The resulting alignments were inspected in MEGA11 (v11.0.13) [35] for comparative analysis. Pseudogenes were identified on the basis of frameshift-causing insertions or deletions, premature stop codons, or loss of recognizable start codons, with comparisons made against intact homologous sequences. This strategy was applied to analyses of *rpl22*, *ycf68*, *orf56*, *cemA*, *ycf15*, *rps4*, and *ycf1*. Where raw sequencing reads were available, candidate pseudogenization events were further validated by visual inspection of read alignments in IGV. Read coverage across the affected regions was examined to confirm that the sequence variants responsible for pseudogenization were supported by the underlying raw reads rather than resulting from assembly or annotation errors.

To assess whether similar pseudogenization states corresponded to particular phylogenetic branches, pseudogenization patterns were mapped onto an Aurantioideae-centered plastome phylogeny that included 14 additional Rutaceae accessions for comparison. We focused on the distribution of repeated pseudogenization involving *rpl22*, *ycf68*, and *orf56*, and separately examined lineage-specific events involving *cemA*, *ycf15*, *rps4*, and *ycf1*. The phylogenetic interpretation of these events was based on the whole-plastome trees described above.

### Divergence-time estimation

Divergence times were estimated using MCMCtree in PAML v4.10.10 [36] with the approximate-likelihood method [37]. The ML topology inferred from the plastome alignment using IQ-TREE v2.3.6 [38] was fixed for molecular dating and rooted with *Ailanthus altissima* as the outgroup. The analysis included 47 taxa and a 77,661-bp nucleotide alignment. Branch lengths, gradient vectors, and the Hessian matrix required for the approximate-likelihood calculation were estimated using BASEML under the HKY85 + Γ model with five discrete gamma rate categories and subsequently used in MCMCtree [37,39,40].

Four temporal constraints were applied following published fossil assessments and divergence estimates. The root separating *A. altissima* from the sampled Rutaceae was assigned a secondary soft bound of 100–117 Ma [B (1.00, 1.17)] [41]. Fossil-based soft minimum constraints were assigned to stem *Zanthoxylum* at 47.8 Ma [L (0.478)] [41], the stem *Clausena* + *Glycosmis* lineage at 27.23 Ma [L (0.2723)] [42], and stem *Citrus* at 11.6 Ma [L (0.116)] [43]. A prior-only analysis (usedata = 0) was first performed to evaluate the effective time priors. Posterior dating was then conducted using the approximate likelihood (usedata = 2) under an independent-rates relaxed clock (clock = 2) with a birth–death-sampling prior (BDparas = 1 1 0.1 m), rgene_gamma = 2 20 1, and sigma2_gamma = 1 10 1. Two independent MCMC chains were run, each with 200,000 burn-in iterations followed by 20,000 retained samples collected every 100 iterations. Convergence was assessed using rank-normalized R-hat and bulk and tail effective sample sizes [44]. Calibration sensitivity was assessed by replacing the 47.8-Ma minimum-age constraint for *Zanthoxylum* with a 66-Ma minimum-age constraint based on the fossil *Rutaspermum biornatum* [41], while keeping all other analytical settings unchanged.

### Use of generative AI

ChatGPT (https://chat.openai.com/) was used to rewrite the manuscript into more appropriate English and proofread the English text. Subsequently, the authors verified the correctness of the revisions.

## Results

### Aurantioideae plastomes were highly conserved with localized genomic and sequence variation

A total of 103 plastome accessions were analyzed, comprising 88 Aurantioideae accessions and 15 outgroup taxa (S3 Table). Among the Aurantioideae accessions, plastome size ranged from 158,779 bp in *Naringi crenulata* to 161,172 bp in *Clausena excavata*. Overall GC content varied by only 0.39 percentage points, ranging from 38.28% in *C. excavata* to 38.67% in *Clausena harmandiana* and *Clausena lansium*. Gene content was also highly uniform across the subfamily. All 88 Aurantioideae plastomes contained 30 unique tRNA genes and four unique rRNA genes. Most accessions (85 of 88) contained 83 unique protein-coding genes, corresponding to a total of 117 unique genes per plastome (S3 Table).

Whole-plastome sequence conservation was further evaluated among representative Aurantioideae taxa using mVISTA, with *Citrus sinensis* as the reference plastome (S1 Fig). The comparison included representatives from multiple genera across the subfamily, including *Aegle*, *Atalantia*, *Citropsis*, *Clausena*, *Citrus*, *Glycosmis*, *Micromelum*, *Murraya*, *Naringi*, *Paramignya*, *Swinglea*, and *Triphasia*. Coding regions were generally more conserved than non-coding regions, whereas greater sequence divergence was observed mainly in intergenic and other non-coding regions (S1 Fig). The inverted-repeat regions, including the regions containing the rRNA genes, were highly conserved across the sampled taxa. In contrast, localized reductions in sequence similarity were evident at several positions within the large single-copy and small single-copy regions. Despite these localized differences, the overall alignment profiles were highly similar among the representative plastomes (S1 Fig).

### Structural variation at the LSC–IR boundaries and repeated putative pseudogenization across Aurantioideae plastomes

Despite the overall sequence conservation observed among Aurantioideae plastomes, structural variation was detected at the LSC–IR boundaries (Fig 1). Comparative analysis revealed pronounced variation in the regions containing *rpl22* and *rps19*, particularly at the LSC–IRB and IRA–LSC junctions (Fig 1). These differences included gene fragmentation, putative pseudogenization, and local gene loss, indicating substantial structural variation at the LSC–IR boundaries among the examined taxa.

**Fig 1:**
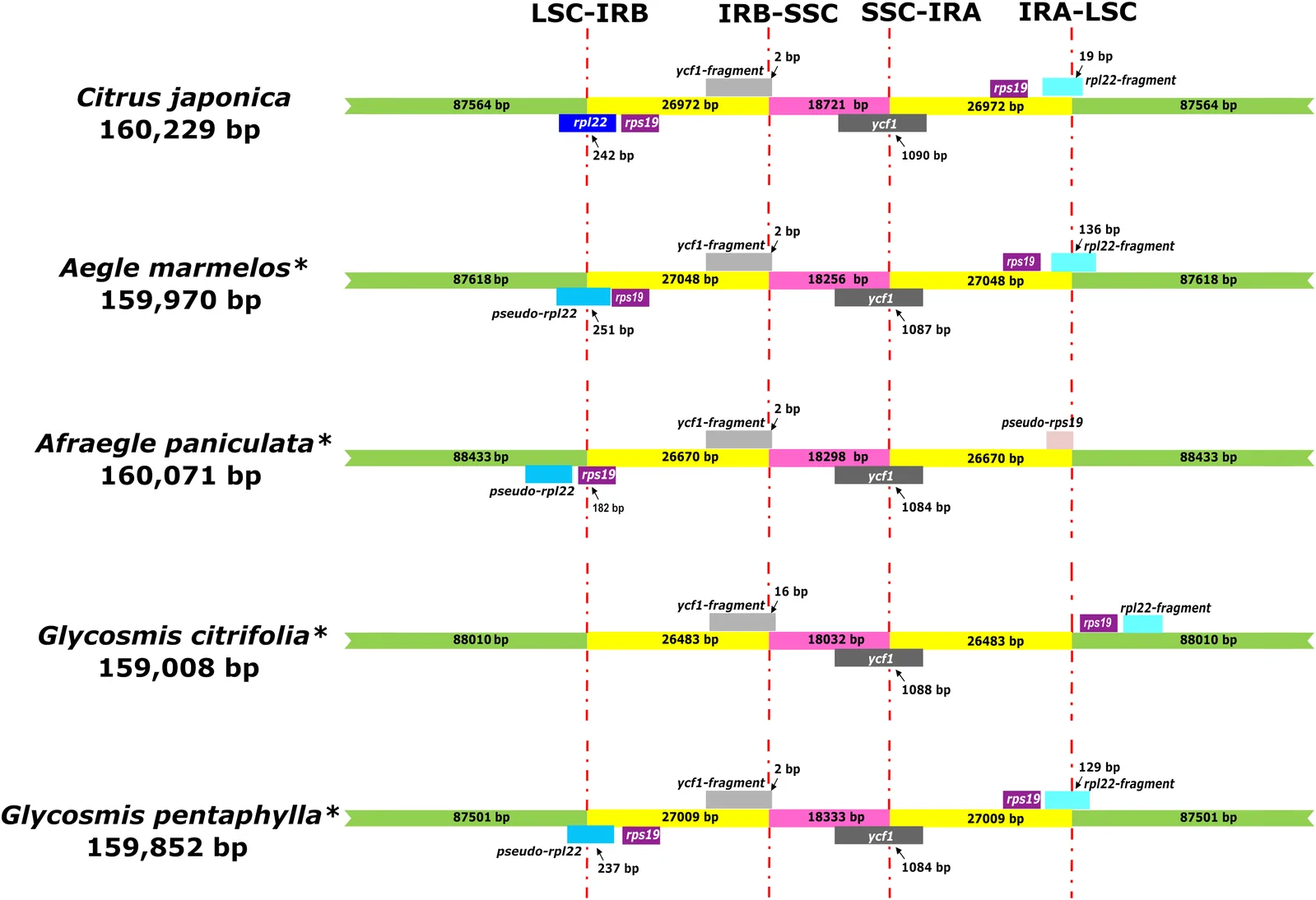
Structural variation at plastome boundary regions in representative Aurantioideae species. The structures of the LSC–IRB, IRB–SSC, SSC–IRA, and IRA–LSC junctions are compared among representative Aurantioideae plastomes. Gene positions and orientations near the boundaries are shown together with the lengths of adjacent regions. Fragmentation, pseudogenization, and local loss involving *rpl22* and *rps19* are evident in several lineages. These comparisons illustrate that plastome boundary regions are structurally variable within Aurantioideae, especially around the LSC–IR boundaries. Asterisks indicate sequences assembled de novo.

The *rpl22* region showed considerable variation among the examined species. Two *rpl22*-associated sequences were identified near the LSC–IR junctions (Fig 1). The sequence located at the LSC–IRB boundary generally retained the expected gene length with identifiable start and stop codons, whereas the corresponding sequence at the IRA–LSC boundary was truncated or fragmented relative to the intact gene (Fig 1). A representative configuration is shown for *Citrus japonica* in Fig 1. In several lineages, the LSC–IRB-associated *rpl22* copy was inferred to be pseudogenized, primarily through the occurrence of internal premature stop codons (Figs 1 and 2). Notably, *rpl22* was absent from the corresponding boundary region in *Glycosmis citrifolia*, whereas *Glycosmis pentaphylla* retained a putatively pseudogenized copy at this position (Figs 1 and 2).

**Fig 2:**
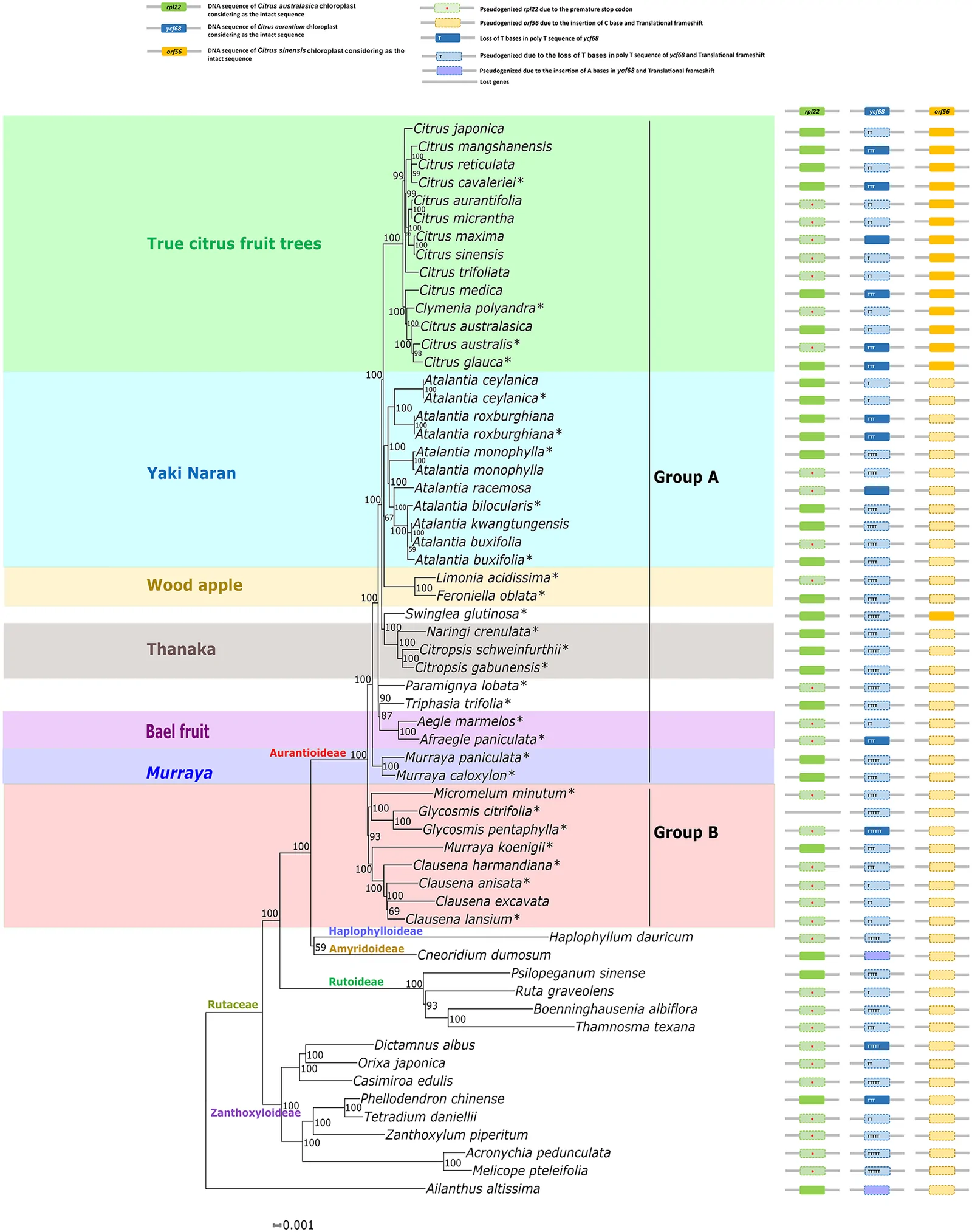
Distribution of *rpl22*, *ycf68*, and *orf56* pseudogenization across Aurantioideae and additional Rutaceae accessions. The phylogenetic tree shows the distribution of pseudogenization states of *rpl22*, *ycf68*, and *orf56* across Aurantioideae, with 14 additional Rutaceae accessions included for comparison. Intact reference sequences were defined from *Citrus australasica* (*rpl22*), *Citrus aurantium* (*ycf68*), and *Citrus sinensis* (*orf56*), and all other sequences were evaluated relative to these references. Colored symbols indicate pseudogenization states or gene loss in each lineage. Representative mutation types are summarized on the right: premature stop codons in *rpl22* (green symbols with internal asterisks), C insertion–induced frameshifts in *orf56* (yellow dashed symbols), and multiple patterns in *ycf68*, including poly-T deletions (dark blue symbols with internal T counts), associated frameshifts (light blue dashed symbols with internal T counts), and A insertions (blue symbols). Gene loss is indicated by gray lines. These results demonstrate that similar pseudogenization states occurred in multiple phylogenetically separated lineages. Asterisks indicate sequences assembled de novo.

Variation was also observed in *rps19* at the plastome boundaries. In *Afraegle paniculata*, the *rps19* sequence located at the IRA–LSC boundary was inferred to be pseudogenized, whereas the corresponding gene remained intact in *Aegle marmelos* (Fig 1). In addition, *rps19* was absent from the LSC–IRB boundary of *Glycosmis citrifolia* (Fig 1).

Beyond these boundary-associated changes, putative plastid gene pseudogenization was assessed across Aurantioideae together with 14 additional Rutaceae accessions (Fig 2). The *infA* gene was inferred to be pseudogenized in all examined taxa, whereas putative pseudogenization of *ycf68* and *orf56* occurred in multiple lineages across the phylogeny (Fig 2). Sequence comparisons showed that *orf56* pseudogenization was associated with the insertion of a cytosine nucleotide, resulting in a frameshift mutation (Fig 2). Similarly, *ycf68* pseudogenization was associated with either the deletion of thymine residues within a poly-T region or the insertion of adenine residues, both of which resulted in frameshift mutations (Fig 2). Representative IGV read alignments showed that the mutations associated with putative pseudogenization of *rpl22*, *orf56* and *ycf68* were consistently supported by mapped sequencing reads (S2 Fig for *rpl22* and o*rf56*; S3 Fig for *ycf68*).

### Additional lineage-specific putative pseudogenization events were detected in *cemA*, *ycf15*, *rps4*, and *ycf1*

In addition to the more broadly distributed pseudogenization patterns described above, several lineage-specific events were identified at the sequence level (S4–S6 Figs). In *Afraegle paniculata*, *cemA* was inferred to be pseudogenized relative to the intact sequence in *Aegle marmelos*, with a 5-bp insertion of adenine residues disrupting the coding sequence (S4 and S5 Figs). In *Aegle marmelos*, *ycf15* lacked a recognizable start codon compared with the intact sequence in *Citrus sinensis* (S4 and S5 Figs). Putative pseudogenization of *rps4* was also detected in *C. sinensis* (S6 Fig).

A further lineage-specific event was detected in *Atalantia kwangtungensis*, in which *ycf1* was inferred to be pseudogenized relative to the intact gene in *Atalantia buxifolia* (S6 Fig). This disruption was associated with deletion of a thymine nucleotide, resulting in disruption of the reading frame.

### Whole-plastome phylogeny resolved detailed relationships within *Citrus*

ML analysis was conducted using complete plastome sequences from 56 *Citrus* accessions with *Atalantia buxifolia* designated as the outgroup (Fig 3). The final alignment comprised 88,414 nucleotide positions, including 1,672 parsimony-informative sites. The resulting topology showed high overall resolution, with most major and several species-level relationships receiving strong bootstrap support. Eight major plastome-defined groups were recovered: the Pummelo, Papeda, Ichang Papeda/Yuzu, Mandarin, Poncirus, Fortunella, Citron, and Australian clusters (Fig 3).

**Fig 3:**
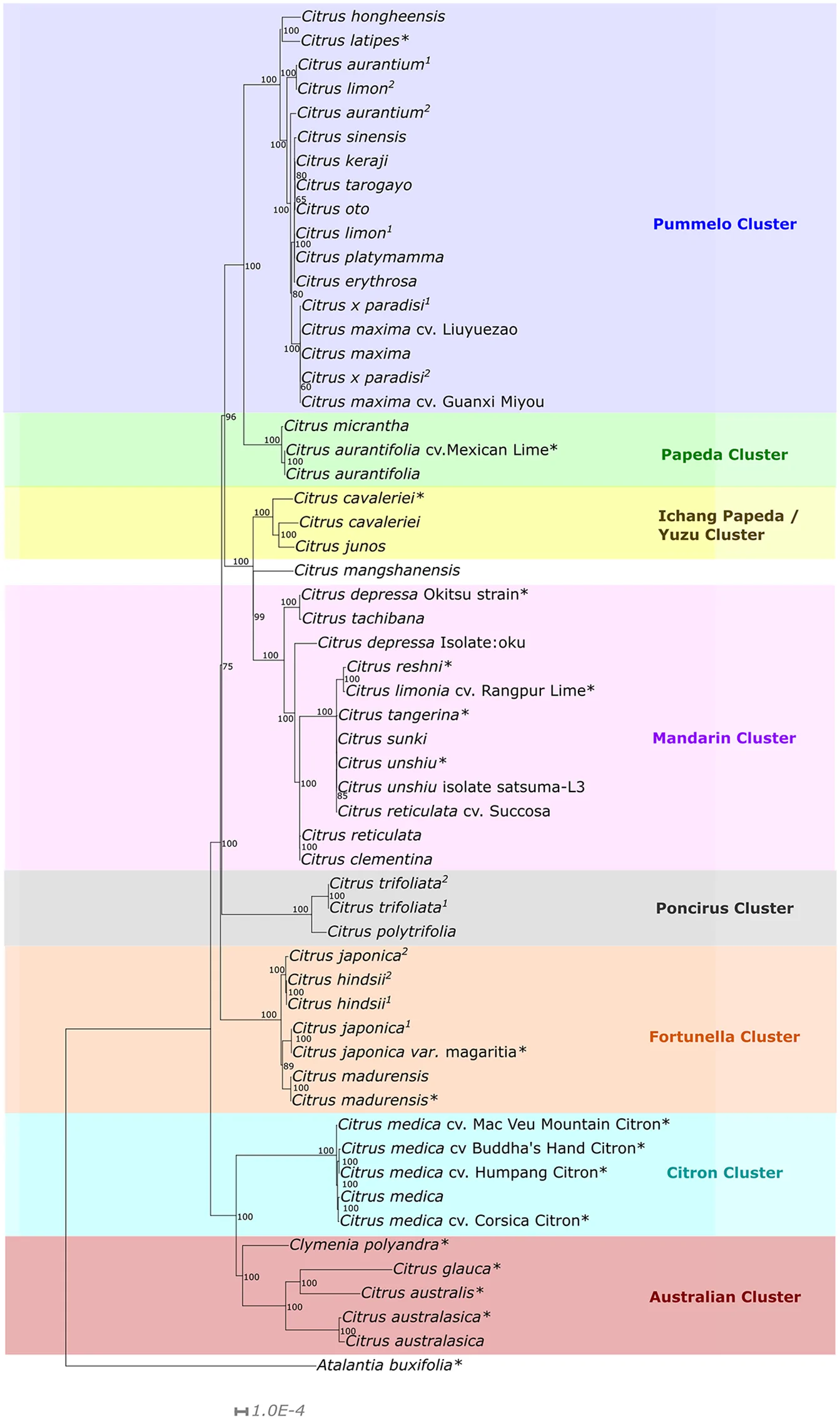
ML phylogenetic tree of *Citrus* based on complete plastome sequences. The tree was constructed using complete plastome sequences from 56 *Citrus* accessions with *Atalantia buxifolia* used as the outgroup. For this analysis, the optimal evolutionary model (GTRGAMMAIX) was implemented in RAxML. The alignment used for tree construction contained 88,414 nucleotide positions, including 1,672 parsimony-informative sites. Bootstrap values are shown at the nodes. Superscript numbers distinguish different accessions assigned to the same species name. The eight major plastome clusters recognized in this study are indicated by different colors. The scale bar represents genetic divergence in substitutions per site. Sequences assembled de novo are denoted by asterisks.

The Pummelo Cluster comprised the largest assemblage of accessions and included *C. maxima*, *C. sinensis*, *C. aurantium*, *C. limon*, *C. × paradisi*, and several other cultivated taxa (Fig 3). Notably, the two wild species *C. hongheensis* and *C. latipes* were also placed within the Pummelo Cluster and formed a strongly supported sister pair (BS = 100) (Fig 3). The sampled *C. maxima* accessions occurred within the same broader plastome lineage as grapefruit, sweet orange, sour orange, and lemon accessions. The Papeda Cluster comprised *C. micrantha* and two *C. aurantiifolia* accessions and received strong bootstrap support (BS = 100) (Fig 3). *Citrus cavaleriei* and *C. junos* formed a separate, strongly supported Ichang Papeda/Yuzu Cluster (Fig 3).

A distinct phylogenetic position was observed for *C. mangshanensis*, which occurred outside the defined Mandarin Cluster rather than grouping with the core mandarin accessions (Fig 3). The Mandarin Cluster comprised *C. depressa*, *C. tachibana*, *C. reshni*, *C. limonia*, *C. tangerina*, *C. sunki*, *C. unshiu*, *C. reticulata*, and *C. clementina*, with strong support for several internal relationships. Within this cluster, the two *C. reticulata* accessions grouped with *C. clementina*, whereas *C. tangerina*, *C. sunki*, and the *C. unshiu* accessions formed a separate sublineage (Fig 3).

The Poncirus Cluster contained two *C. trifoliata* accessions together with *C. polytrifolia*, whereas the Fortunella Cluster comprised *C. japonica*, *C. hindsii*, *C. japonica* var. *margaritia*, and *C. madurensis* (Fig 3). All sampled *C. medica* accessions were confined to a highly supported Citron Cluster, including Mac Veu Mountain Citron, Buddha’s Hand Citron, Humpang Citron, Corsica Citron, and *C. medica* (Fig 3).

The Australian Cluster comprised *Clymenia polyandra*, *C. glauca*, *C. australis*, and *C. australasica* (Fig 3). Within this lineage, *C. polyandra* was resolved as the earliest-diverging taxon, followed by *C. glauca*, whereas *C. australis* and the two *C. australasica* accessions occupied more derived positions (Fig 3). The two *C. australasica* accessions formed a strongly supported sister pair (BS = 100) (Fig 3).

BI based on the same plastome dataset produced a topology highly congruent with the ML analysis (S7 Fig), recovering the same eight major plastome clusters and principal interspecific relationships, with most nodes receiving high posterior probabilities.

### Divergence-time analysis suggested two comparable Asia–Africa splits within Aurantioideae

To place plastome diversification in a temporal framework, we estimated divergence times using MCMCtree for Aurantioideae and four additional Rutaceae accessions, with *Ailanthus altissima* as the outgroup (Fig 4). Calibration sensitivity was assessed by replacing the *Zanthoxylum*-based minimum constraint with an alternative *Rutaspermum*-based constraint. This analysis produced a comparable overall temporal pattern and retained the relative ordering of the principal divergence events, indicating that these conclusions were not strongly affected by the alternative calibration tested. Subgroup names followed our previous study [9], in which Aurantioideae classifications were based on conserved nuclear genes and plastome data.

**Fig 4:**
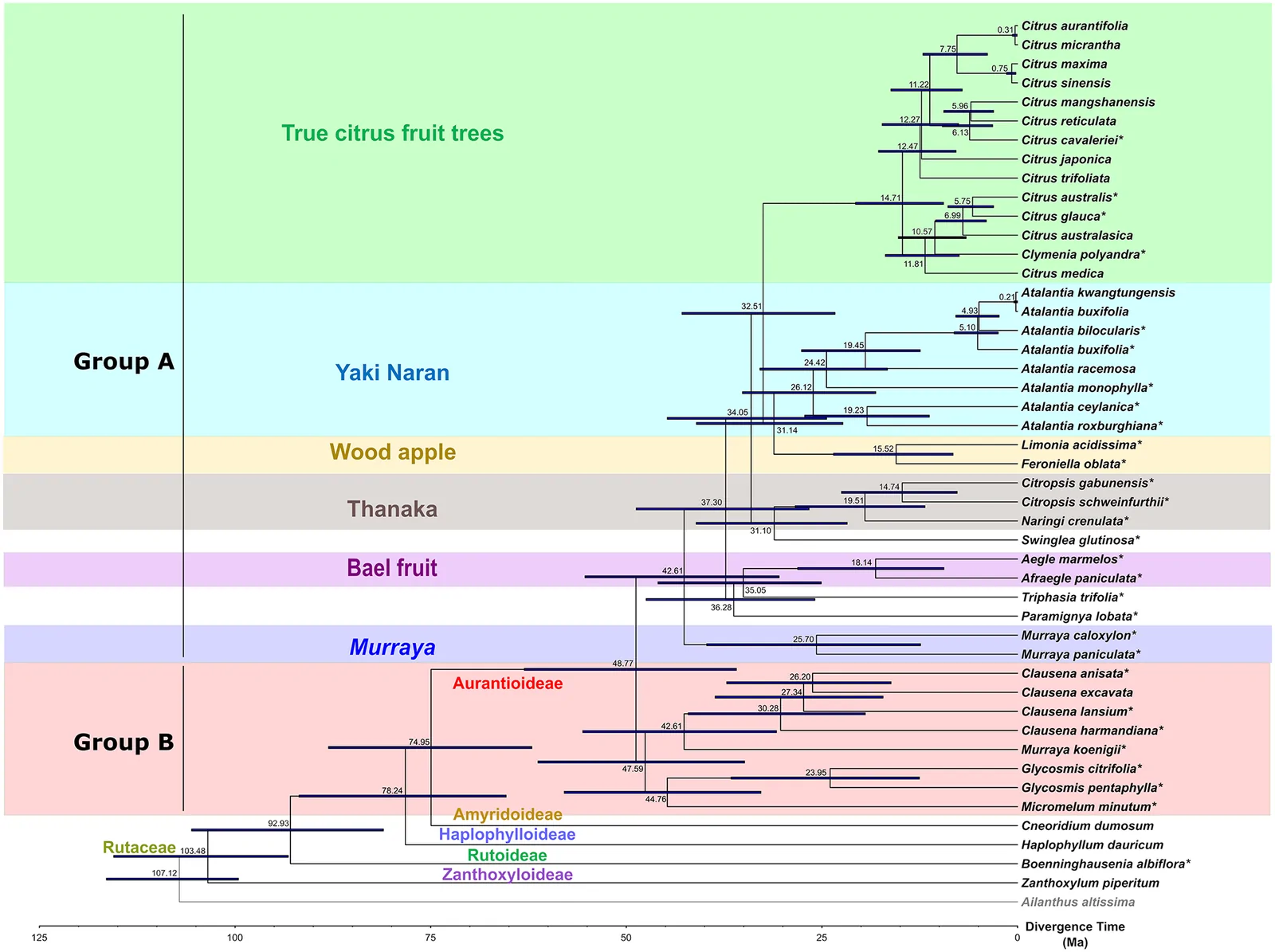
Divergence-time tree of Aurantioideae inferred from complete plastome sequences. Divergence times were estimated using MCMCtree based on the fixed maximum-likelihood topology inferred with IQ-TREE. The analysis included 47 taxa, with *Ailanthus altissima* as the outgroup. Four temporal constraints were applied: a secondary root calibration of 100–117 Ma and fossil-based minimum-age constraints of 47.8 Ma for stem *Zanthoxylum*, 27.23 Ma for the stem *Clausena* + *Glycosmis* lineage, and 11.6 Ma for stem *Citrus*. Node bars represent 95% highest posterior density (HPD) intervals, and the scale indicates divergence time in millions of years ago (Ma). The two Asia–Africa lineage splits discussed in the text are indicated for *Citropsis*–*Naringi* and *Aegle*–*Afraegle*. Asterisks indicate plastomes assembled de novo from raw sequence data.

Two biogeographically parallel lineage separations were identified within Aurantioideae (Fig 4). In the Thanaka group, African *Citropsis* and Asian *Naringi* diverged at an estimated 19.51 Ma (95% HPD: 11.85–28.40 Ma). In the Bael fruit group, African *Afraegle* and Asian *Aegle* diverged at 18.14 Ma (95% HPD: 9.43–28.08 Ma) (Fig 4). Their similar posterior mean estimates and broadly overlapping HPD intervals indicate comparable early Miocene divergence times.

Divergences involving Australian *Citrus* had younger posterior mean estimates, although their HPD intervals overlapped those of the two Asia–Africa separations (Fig 4). The lineage comprising Australian *Citrus*, *Clymenia polyandra*, and *Citrus medica* diverged from the remaining sampled true citrus fruit trees at 14.71 Ma (95% HPD: 9.47–20.69 Ma) (Fig 4). Within this lineage, *C. medica* separated from *Clymenia polyandra* and Australian *Citrus* at 11.81 Ma (95% HPD: 7.47–16.88 Ma), followed by the separation of *Clymenia polyandra* from Australian *Citrus* at 10.57 Ma (95% HPD: 6.58–15.22 Ma; Fig 4).

## Discussion

The present study provides an integrated assessment of plastome evolution and diversification within Aurantioideae through comparative plastome analyses, evaluation of pseudogenization patterns, phylogenetic inference, and divergence-time estimation. Aurantioideae plastomes were highly conserved in overall organization and gene content, with localized variation concentrated at LSC–IR boundaries and in genes exhibiting putative pseudogenization. This combination of broad conservation and localized change provides a framework for examining structural and coding-sequence evolution alongside phylogenetic relationships. Some of these plastome-based analyses were initially reported in our earlier preprint [17] but had not previously been developed in peer-reviewed form.

The conserved organization and largely uniform gene content observed among Aurantioideae plastomes indicate that substantial lineage diversification has occurred without extensive changes in their overall genomic architecture. Nevertheless, this conservation does not imply structural or functional uniformity at individual loci. Comparative analyses revealed boundary-associated modifications and disruptive sequence changes that were not apparent from overall plastome size or gene counts alone. Consequently, general plastome characterization and detailed comparisons of individual regions provide complementary perspectives on plastome evolution within the subfamily.

Variation at the LSC–IR boundaries was particularly evident around *rpl22* and *rps19*, including fragmentation, putative pseudogenization, and local gene loss. Similar boundary-associated changes have been reported in other angiosperm lineages. In Acanthaceae, variation at the LSC–IR junction has been associated with pseudogenization of *rps19* and, in some taxa, *rpl22* [45], whereas Urticeae exhibits repeated boundary shifts involving these genes and occasional gene loss [46]. Comparable, although more limited, variation around *rps19* has also been documented in *Prunus* [47]. These observations show that local structural variation can occur within otherwise conserved plastomes. The repeated occurrence of boundary modifications across lineages suggests that plastome junctions are evolutionarily labile regions. Boundary architecture may therefore provide informative evolutionary characters, although recurrent changes should be considered when interpreting their distribution across a phylogeny.

The distribution of putative pseudogenization events further indicates that localized plastome evolution in Aurantioideae has involved changes at multiple loci. Recurrent sequence disruptions affecting *rpl22*, *ycf68*, and *orf56* suggest that coding-sequence degeneration has occurred through different mutational routes. Comparable patterns have been reported elsewhere in angiosperms. For example, *infA* has been independently lost or pseudogenized multiple times, in several cases following functional transfer to the nucleus [48], while repeated plastome loss of *rpl22* has been associated with nuclear functional replacement [49,50]. By contrast, *ycf68* appears to be nonfunctional or weakly constrained in many angiosperms [51], emphasizing the need for caution when interpreting sequence disruptions in genes of uncertain functional status. The patterns detected here may therefore reflect several evolutionary processes, including relaxed selective constraint, lineage-specific structural changes, or, for some genes, functional replacement outside the plastome. However, these mechanisms cannot be distinguished using plastome sequences alone. Recurrent disruptions also caution against treating individual putative pseudogenization events as definitive phylogenetic characters.

In addition to these more broadly distributed patterns, several lineage-specific sequence disruptions warrant further investigation. In *Afraegle paniculata*, the putative pseudogenization of *cemA* [52] raises questions about whether its function has been retained or compensated through another mechanism. Possible explanations include compensation involving the nuclear-encoded *DLDG1* [53] or functional transfer of *cemA* to the nucleus followed by acquisition of a plastid-targeting transit peptide. Neither possibility, however, can be established from the present data. The putative pseudogenization of *ycf15* in *Aegle marmelos* is best interpreted primarily as a lineage-specific sequence feature because the functional status of this gene remains uncertain in many angiosperms [16]. The disruption observed in *rps4* in *Citrus sinensis* is also noteworthy because the sweet orange plastome has been widely used as a reference in citrus plastome studies, yet this feature was not explicitly discussed in the original plastome report [54].

In *Atalantia kwangtungensis*, we recovered sequence-level evidence consistent with the previously reported putative pseudogenization of *accD* [55] and additionally identified disruptive changes in *ycf1*. The *accD* gene encodes a subunit of acetyl-CoA carboxylase [56,57], whereas *ycf1* has been proposed to encode a component of the plastid protein translocon [58]. Because co-evolution between *accD* and *ycf1* has previously been suggested [58], the occurrence of disruptive changes in both genes within the same lineage may warrant further functional investigation. Their co-occurrence alone, however, does not demonstrate a functional relationship or a shared mechanism of degeneration. Collectively, the lineage-specific changes identified here provide sequence-level evidence of putative pseudogenization. Raw-read support for the underlying mutations strengthens confidence in the sequence observations but does not establish loss of gene function. Transcriptomic, nuclear-genome, and functional analyses will be required to determine whether these changes are associated with functional loss, compensation, or gene transfer.

Alongside the localized structural and coding-sequence changes identified here, the whole-plastome analysis of 56 accessions recovered eight major clusters within the sampled *Citrus* assemblage: Pummelo, Papeda, Ichang Papeda/Yuzu, Mandarin, Poncirus, Fortunella, Australian, and Citron. *Clymenia polyandra* was sister to the sampled Australian species, with this assemblage sister to the Citron cluster. Its position within the true citrus group agrees with the expanded circumscription of *Citrus* supported by Bayer et al. (2009) [12]. Although the name *Clymenia polyandra* is retained here following the NCBI taxonomy database, Plants of the World Online (POWO) recognizes it as a synonym of *Citrus polyandra*; its inclusion within *Citrus* therefore accords with established phylogenetic and taxonomic treatments.

Several relationships corroborate earlier plastome studies. Our previous *matK* analysis of 93 accessions identified three principal groups corresponding to citron, pummelo, and mandarin, with *Poncirus* and *Fortunella* outside these groups [10]. Subsequently, Carbonell-Caballero et al. (2015), analyzing 34 plastomes, associated citron with Australian citrus, pummelo with *C. micrantha*, and mandarins with Ichang papeda, while recognizing *Poncirus* and *Fortunella* as distinct lineages [11]. Our tree similarly places the Citron cluster sister to the Australian assemblage, recovers sister Pummelo and Papeda clusters, and associates Ichang Papeda/Yuzu with the broader mandarin-related assemblage, while retaining distinct Poncirus and Fortunella clusters. The eight-cluster scheme therefore describes finer subdivisions within several previously recognized broader relationships. At the accession level, the placement of *C. sinensis*, *C. aurantium*, *C. × paradisi*, and *C. limon* within the Pummelo cluster, the association of *C. aurantifolia* with *C. micrantha*, and the inclusion of *C. limonia* within the Mandarin cluster are consistent with the maternal affinities reported by Carbonell-Caballero et al. (2015) [11]. These relationships illustrate how cultivated accessions with different taxonomic identities can share closely related plastomes, reflecting their maternal ancestry.

Relative to our earlier *matK* analysis, which emphasized citron, pummelo, and mandarin groups [10], the present tree provides a more differentiated description of the sampled plastome lineages. However, Jena et al. (2009) also recovered eight major clusters using PCR-RFLP data, so the cluster count itself should not be regarded as novel [59]. Our contribution is to reconstruct and characterize these relationships using complete plastome sequences, extending sequence coverage beyond selected markers. Oueslati et al. (2016), by comparison, developed diagnostic SNP markers from eight plastome regions to identify established Aurantioideae clades [8]. Their marker-based identification and our phylogenetic reconstruction serve related purposes, but their clades span multiple taxonomic levels and are not directly equivalent to our eight clusters.

The occurrence of cultivated hybrids within different plastome clusters also illustrates why these groups should be interpreted as maternal lineages rather than species boundaries. Nuclear genomic analyses have demonstrated extensive hybridization and admixture in *Citrus*, explaining how accessions sharing a plastome lineage can have different nuclear ancestries [5]. Our phylogeny therefore provides a framework for interpreting the structural variation, putative pseudogenization, and divergence times examined here, while nuclear evidence remains essential for reconstructing the broader reticulate history of *Citrus*.

The divergence-time analysis extends this phylogenetic framework by placing major lineage separations within a temporal context. The estimated separations between *Citropsis* and *Naringi* and between *Afraegle* and *Aegle*, approximately 19.5 and 18.1 Ma, respectively, fall within the early Miocene. These broadly comparable estimates suggest a temporal parallel between two lineages exhibiting present-day Asia–Africa disjunctions, although similar point estimates do not establish synchronous geographic isolation or a shared cause. Progressive Himalayan–Tibetan uplift and associated changes in atmospheric circulation, monsoon dynamics, and habitat distribution provide a possible environmental context for changes in the ranges of their Asian ancestors [60]. However, these prolonged geological processes cannot be directly linked to the inferred separations, and dispersal, range fragmentation, and regional extinction remain alternative explanations for the observed distributions.

The younger divergences involving Australian *Citrus* may instead be considered in the context of changing dispersal opportunities between Southeast Asia and Australasia. Increased Miocene floristic exchange between the Sunda and Sahul regions provides a plausible regional framework but does not establish the route or timing of *Citrus* migration into Australia [61]. Wu et al. (2018) previously inferred a late Miocene radiation of Asian *Citrus*, followed by an early Pliocene radiation of Australian limes [5]. The present study offers a complementary plastome-based assessment within a broader Aurantioideae framework. However, the deeper separations examined here are not equivalent to diversification within the Australian clade and therefore do not directly confirm the previously reported radiation age. These findings highlight temporal variation among geographically differentiated lineages within Aurantioideae. Their biogeographic interpretation remains conditional on age uncertainty and the distinction between plastome divergence and geographic separation. Ancestral-range reconstruction and independent nuclear evidence will be needed to evaluate the processes underlying these distributions.

Overall, Aurantioideae plastomes combine broadly conserved organization and gene content with localized boundary modifications and lineage-specific sequence degeneration. Complete plastome sequences also provide a detailed framework for interpreting maternal-lineage relationships within *Citrus* and estimating the timing of major lineage divergences. Integrating these perspectives extends the contribution of plastome data beyond sequence-based phylogenetic inference to the study of structural evolution, putative pseudogenization, and lineage diversification within Aurantioideae.

## Supporting information

S1 Fig mVISTA comparison of complete plastome sequences across representative Aurantioideae taxa. Complete plastome sequences were compared using the Shuffle-LAGAN alignment algorithm, with *Citrus sinensis* as the reference. Sequence identity is displayed from 50% to 100%. Annotated genes and their transcriptional orientations are shown above the alignment. Exons, untranslated regions, conserved noncoding sequences, and mRNA regions are indicated by blue, turquoise, pink, and gray, respectively.
(PDF)

S2 Fig IGV validation of mutations associated with pseudogenization of *rpl22* and *orf56.* A) Read alignment supporting the nucleotide substitution that introduced a premature stop codon in *rpl22* of *Afraegle paniculata*. The mutation position was supported by a read depth of 503 × . B) Read alignment supporting the insertion of a cytosine (C) associated with pseudogenization of *orf56* in *Atalantia ceylanica*. The insertion position was supported by a read depth of 613 × . Arrows indicate the mutation positions.
(PDF)

S3 Fig IGV validation of deletions associated with pseudogenization of *ycf68.* A) Read alignment supporting the deletion of three thymine (T) bases within a poly-T region of *ycf68* in *Citrus medica*. The displayed region had a mean read depth of 435.9 × . B) Read alignment supporting the deletion of four T bases within a poly-T region of *ycf68* in *Limonia acidissima*, resulting in a frameshift. The displayed region had a mean read depth of 831 × . Brackets indicate the deleted nucleotides.
(PDF)

S4 Fig Representative lineage-specific pseudogenization events in *cemA* and *ycf15* within Aurantioideae plastomes. A) Pseudogenization of *cemA* in *Afraegle paniculata*. A1) Intact *cemA* sequence in *Aegle marmelos*. A2) Disrupted *cemA* in *Afraegle paniculata*. B) Pseudogenization of *ycf15* in *Aegle marmelos*. B1) Intact *ycf15* sequence in *Citrus sinensis*. B2) Disrupted *ycf15* in *Aegle marmelos*. Insertions and deletions leading to frameshifts or loss of coding potential are highlighted in the alignments.
(PDF)

S5 Fig IGV validation of mutations associated with pseudogenization of *cemA* and *ycf15.* A) Read alignment supporting the insertion of five adenine (A) bases associated with pseudogenization of *cemA* in *Afraegle paniculata*. The displayed region had a mean read depth of 453.9 × . B) Read alignment across the upstream region of *ycf15* in *Aegle marmelos*, confirming read support for the sequence lacking a recognizable start codon. The arrow indicates the region in which a recognizable start codon was absent.
(PDF)

S6 Fig Independent pseudogenization patterns of *rps4* and *ycf1* across Aurantioideae lineages. A) Pseudogenization of *rps4* in *Citrus sinensis*. A1) Intact *rps4* sequence in *Citrus keraji*. A2) Pseudogenized *rps4* in *Citrus sinensis*. B) Pseudogenization of *ycf1* in *Atalantia kwangtungensis*. B1) Intact *ycf1* sequence in *Atalantia buxifolia*. B2) Pseudogenized *ycf1* in *Atalantia kwangtungensis*. Insertions and deletions associated with frameshifts or loss of coding capacity are indicated in the alignments.
(PDF)

S7 Fig BI phylogenetic tree of *Citrus* based on complete plastome sequences. The tree was generated using the same dataset analyzed in Fig 3, consisting of 56 *Citrus* accessions. *Atalantia buxifolia* was used as the outgroup. BI posterior probabilities are shown at the nodes. Superscript numbers distinguish different accessions assigned to the same species name. The eight major plastome clusters recognized in this study are indicated by different colors. The scale bar represents genetic divergence in substitutions per site. Asterisks indicate sequences assembled de novo.
(PDF)

S1 Table List of samples included in this study. The table lists all taxa included in the plastome analyses. For each taxon, it provides the accepted species name, synonym and common name when available, source of plant material when processed by our group, and accession numbers for source raw sequencing reads and previously deposited plastome sequences, where applicable. The table distinguishes sequences generated in our previous study, sequences assembled from publicly available short-read data, and complete plastome sequences obtained from public databases. The de novo assembled and re-annotated plastome files are available in Zenodo (https://doi.org/10.5281/zenodo.22743534).
(XLSX)

S2 Table Summary of filtered sequencing reads and plastome mapping statistics. The table summarizes the sequencing and read-mapping statistics for the 29 accessions generated in our previous study and the 15 accessions assembled from publicly available raw sequencing data. Reported metrics include the number of read pairs, total filtered bases, average read length, percentages of bases with Q20 and Q30 quality scores, GC content of the filtered reads, percentage of reads mapped to the plastome, mean plastome coverage, and mean mapping quality.
(XLSX)

S3 Table Summary of plastome characteristics of the analyzed species. The table provides the accession number, plastome size, GC content, numbers of unique protein-coding, tRNA, and rRNA genes, and total number of unique genes for each taxon. Asterisks indicate plastomes assembled de novo. Superscript numbers distinguish different accessions assigned to the same species name.
(XLSX)
