# Supplementary material for: Plastome evolution in Aurantioideae and plastome-based classification of Citrus
Source: PLoS One. 2026 Oct 7;21(10):e0349882. doi: 10.1371/journal.pone.0349882 (PMC13645046; doi:10.1371/journal.pone.0349882)
Supplement: Supplementary file 1 [file pone.0349882.s001.pdf]

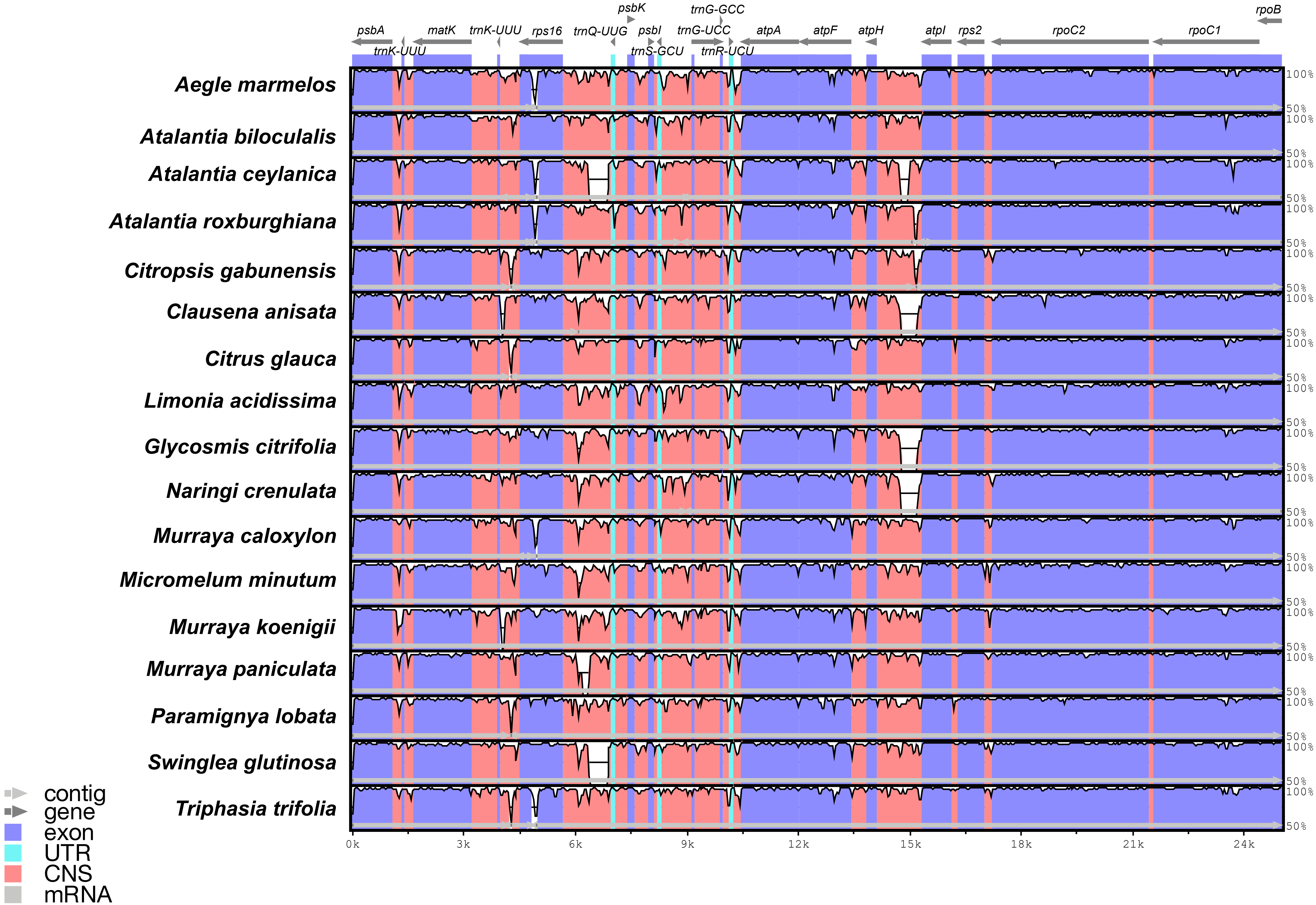

psbA
trnK-UUU
matK
trnK-UUU
rps16
trnQ-UUG
psbK
psbI
trnS-GCU
trnG-GCC
trnG-UCC
trnR-UCU
atpA
atpF
atpH
atpI
rps2
rpoC2
rpoC1
rpoB
Aegle marmelos
Atalantia biloculalis
Atalantia ceylanica
Atalantia roxburghiana
Citropsis gabunensis
Clausena anisata
Citrus glauca
Limonia acidissima
Glycosmis citrifolia
Naringi crenulata
Murraya caloxylon
Micromelum minutum
Murraya koenigii
Murraya paniculata
Paramignya lobata
Swinglea glutinosa
Triphasia trifolia
100%
50%
0k
3k
6k
9k
12k
15k
18k
21k
24k
contig
gene
exon
UTR
CNS
mRNA

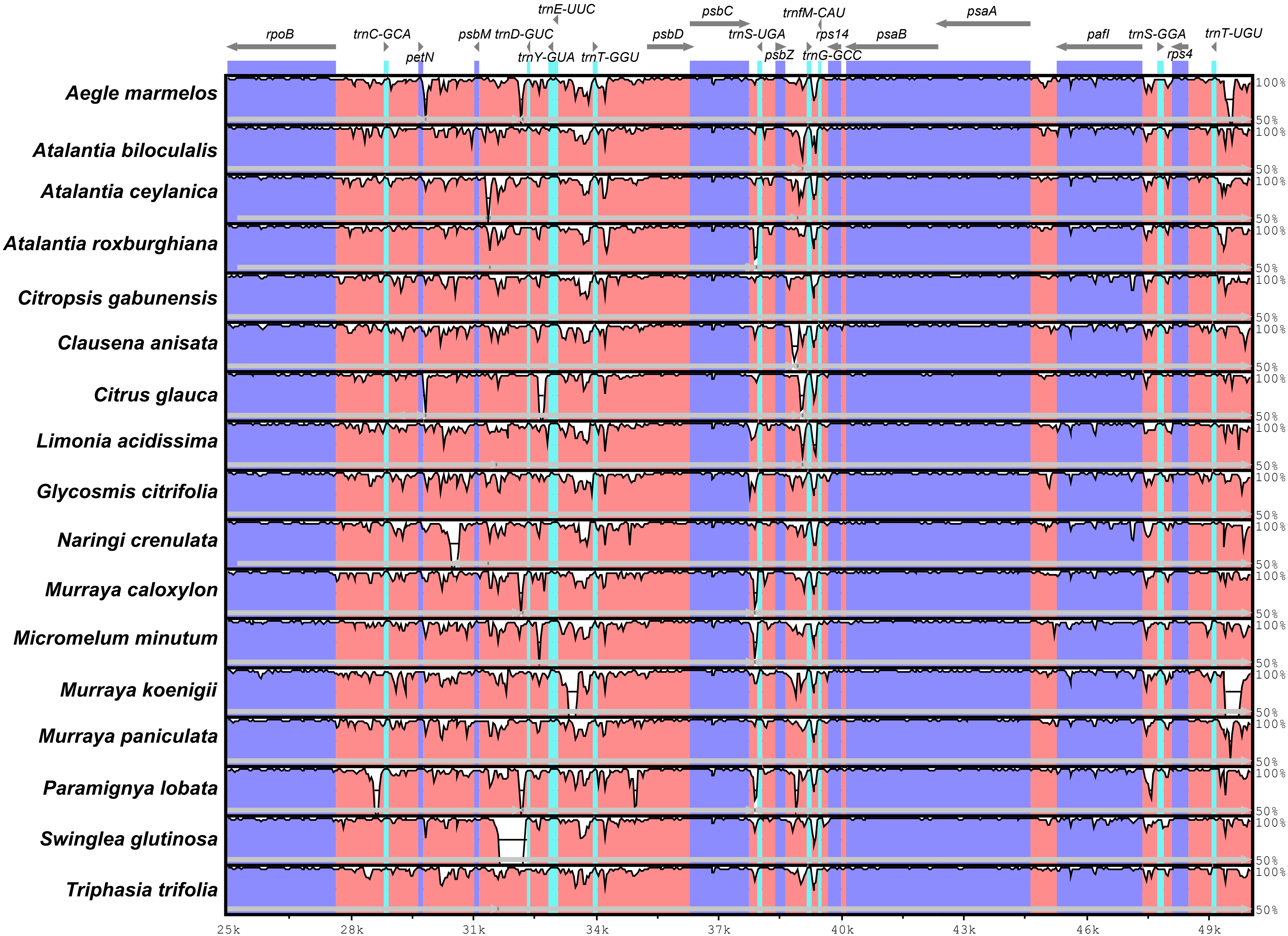

rpoB
trnC-GCA
petN
psbM
trnD-GUC
trnE-UUC
trnY-GUA
trnT-GGU
psbD
psbC
trnS-UGA
psbZ
trnfM-CAU
rps14
trnG-GCC
psaB
psaA
pafI
trnS-GGA
rps4
trnT-UGU
Aegle marmelos
Atalantia biloculalis
Atalantia ceylanica
Atalantia roxburghiana
Citropsis gabunensis
Clausena anisata
Citrus glauca
Limonia acidissima
Glycosmis citrifolia
Naringi crenulata
Murraya caloxylon
Micromelum minutum
Murraya koenigii
Murraya paniculata
Paramignya lobata
Swinglea glutinosa
Triphasia trifolia
100%
50%
25k
28k
31k
34k
37k
40k
43k
46k
49k

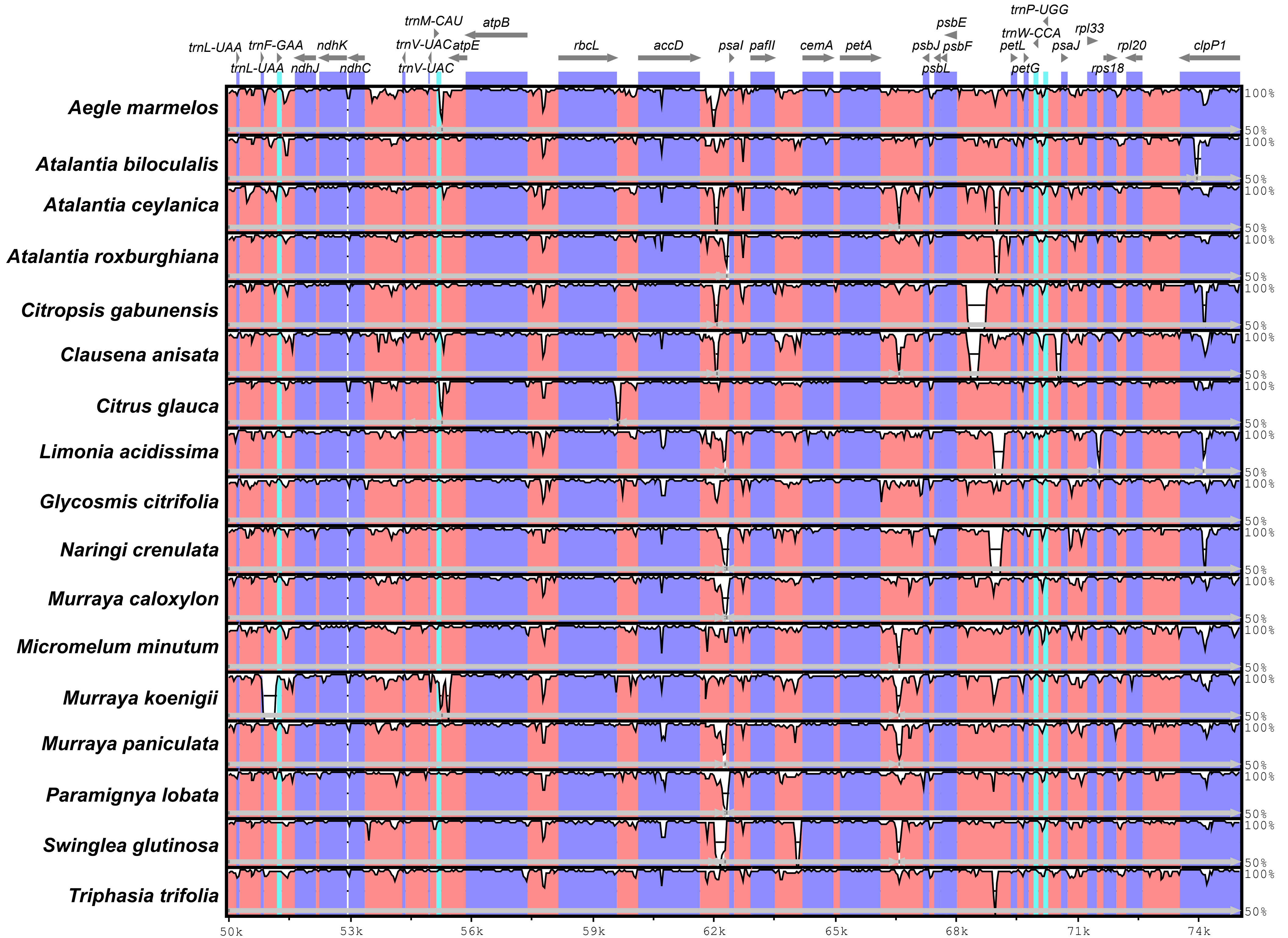

trnP-UGG
trnM-CAU
atpB
psbE
trnW-CCA
rpl33
trnL-UAA
trnF-GAA
ndhK
trnV-UAC
atpE
rbcL
accD
psaI
pafII
cemA
petA
psbJ
psbF
petL
psaJ
rpl20
clpP1
trnL-UAA
ndhJ
ndhC
trnV-UAC
psbL
petG
rps18
Aegle marmelos
Atalantia biloculalis
Atalantia ceylanica
Atalantia roxburghiana
Citropsis gabunensis
Clausena anisata
Citrus glauca
Limonia acidissima
Glycosmis citrifolia
Naringi crenulata
Murraya caloxylon
Micromelum minutum
Murraya koenigii
Murraya paniculata
Paramignya lobata
Swinglea glutinosa
Triphasia trifolia
100%
50%
50k
53k
56k
59k
62k
65k
68k
71k
74k

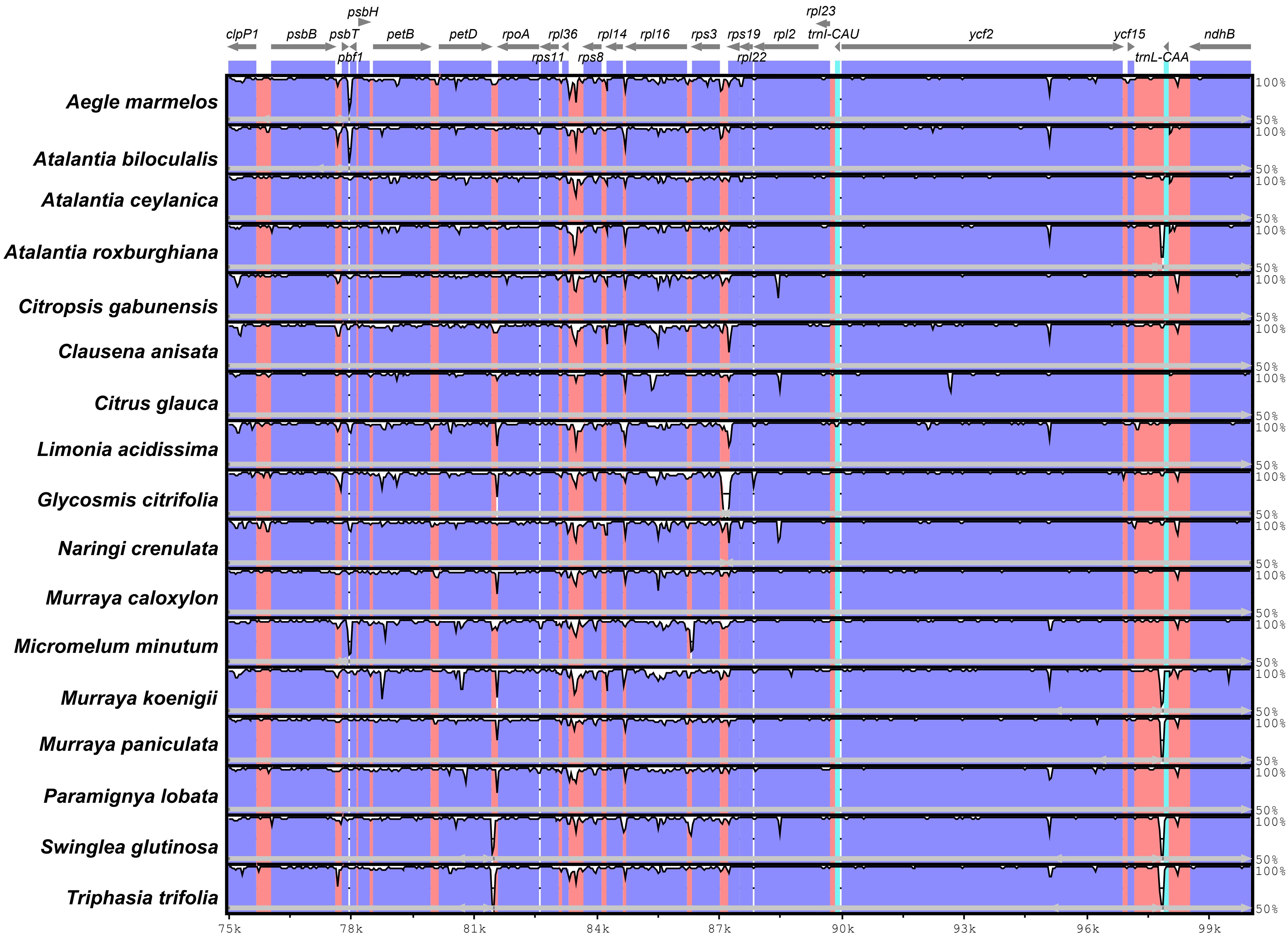

psbH
rpl23
clpP1
psbB
psbT
petB
petD
rpoA
rpl36
rpl14
rpl16
rps3
rps19
rpl2
trnI-CAU
ycf2
ycf15
ndhB
pbf1
rps11
rps8
rpl22
trnL-CAA
Aegle marmelos
Atalantia bilocularis
Atalantia ceylanica
Atalantia roxburghiana
Citropsis gabunensis
Clausena anisata
Citrus glauca
Limonia acidissima
Glycosmis citrifolia
Naringi crenulata
Murraya caloxylon
Micromelum minutum
Murraya koenigii
Murraya paniculata
Paramignya lobata
Swinglea glutinosa
Triphasia trifolia
100%
50%
75k
78k
81k
84k
87k
90k
93k
96k
99k

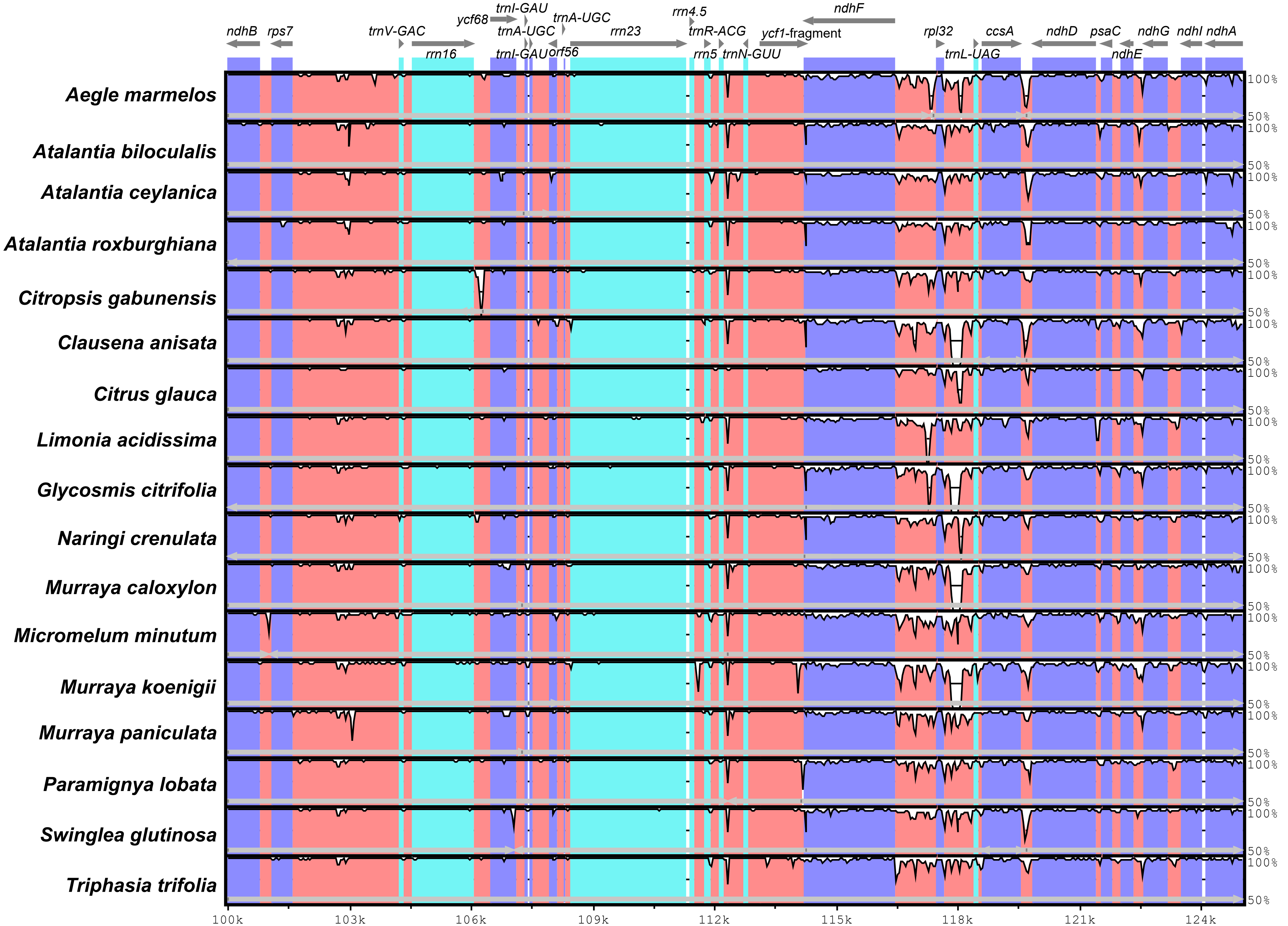
ndhB
rps7
trnV-GAC
rrn16
ycf68
trnI-GAU
trnA-UGC
trnI-GAU
orf56
trnA-UGC
rrn23
rrn4.5
trnR-ACG
rrn5
trnN-GUU
ycf1-fragment
ndhF
rpl32
trnL-UAG
ccsA
ndhD
psaC
ndhE
ndhG
ndhI
ndhA
Aegle marmelos
Atalantia biloculalis
Atalantia ceylanica
Atalantia roxburghiana
Citropsis gabunensis
Clausena anisata
Citrus glauca
Limonia acidissima
Glycosmis citrifolia
Naringi crenulata
Murraya caloxylon
Micromelum minutum
Murraya koenigii
Murraya paniculata
Paramignya lobata
Swinglea glutinosa
Triphasia trifolia
100%
50%
100k
103k
106k
109k
112k
115k
118k
121k
124k

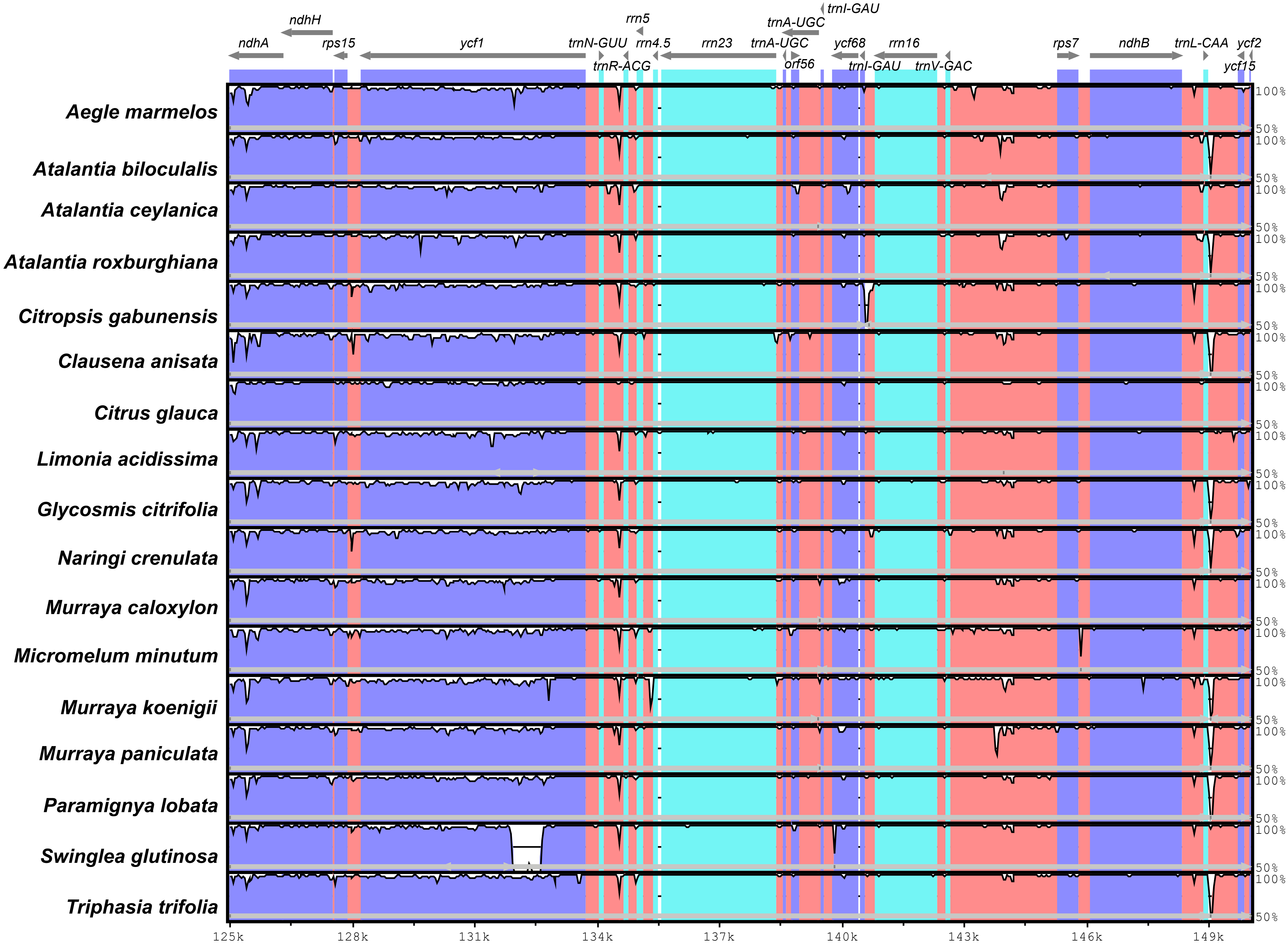

ndhA
ndhH
rps15
ycf1
trnN-GUU
rrn5
trnR-ACG
rrn4.5
rrn23
trnA-UGC
trnA-UGC
trnI-GAU
orf56
ycf68
trnI-GAU
rrn16
trnV-GAC
rps7
ndhB
trnL-CAA
ycf2
ycf15
Aegle marmelos
Atalantia bilocularis
Atalantia ceylanica
Atalantia roxburghiana
Citropsis gabunensis
Clausena anisata
Citrus glauca
Limonia acidissima
Glycosmis citrifolia
Naringi crenulata
Murraya caloxylon
Micromelum minutum
Murraya koenigii
Murraya paniculata
Paramignya lobata
Swinglea glutinosa
Triphasia trifolia
100%
50%
125k
128k
131k
134k
137k
140k
143k
146k
149k

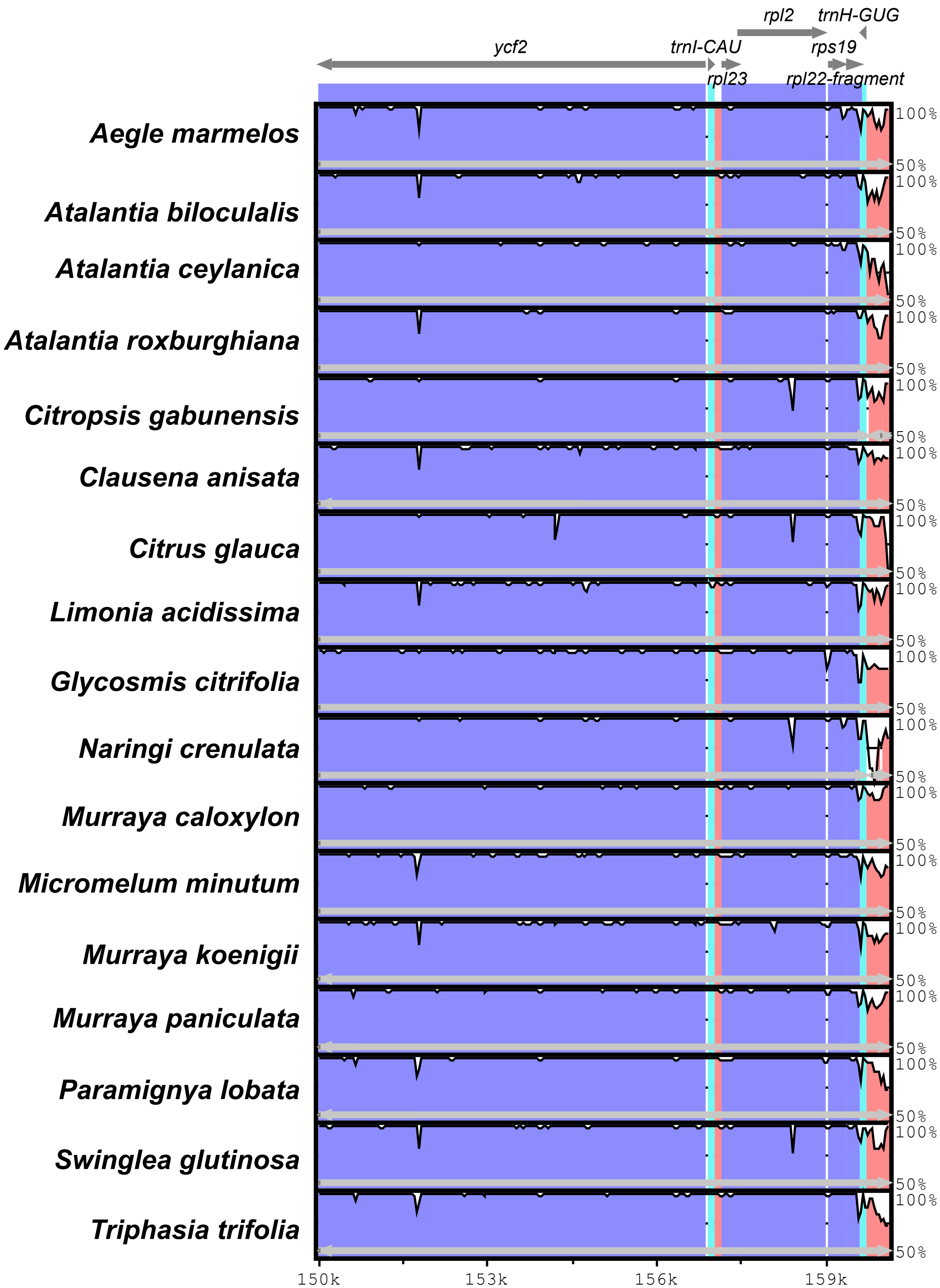

ycf2
trnI-CAU
rpl2
trnH-GUG
rps19
rpl23
rpl22-fragment
Aegle marmelos
Atalantia biloculalis
Atalantia ceylanica
Atalantia roxburghiana
Citropsis gabunensis
Clausena anisata
Citrus glauca
Limonia acidissima
Glycosmis citrifolia
Naringi crenulata
Murraya caloxylon
Micromelum minutum
Murraya koenigii
Murraya paniculata
Paramignya lobata
Swinglea glutinosa
Triphasia trifolia
100%
50%
150k
153k
156k
159k
