# Supplementary material for: Plastome evolution in Aurantioideae and plastome-based classification of Citrus
Source: PLoS One. 2026 Oct 7;21(10):e0349882. doi: 10.1371/journal.pone.0349882 (PMC13645046; doi:10.1371/journal.pone.0349882)
Supplement: Supplementary file 2 [file pone.0349882.s002.pdf]

A)

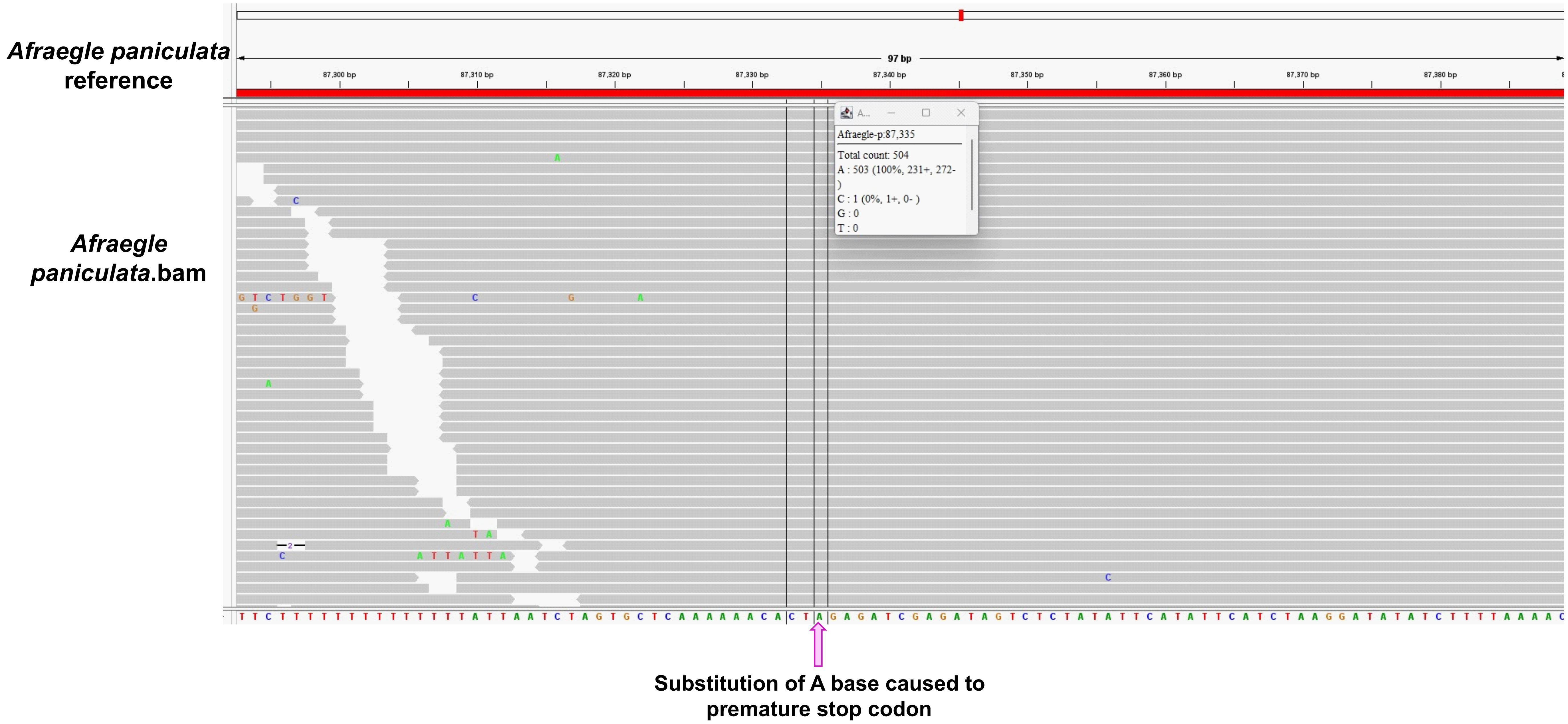
Afraegle paniculata reference
97 bp
87,300 bp
87,310 bp
87,320 bp
87,330 bp
87,340 bp
87,350 bp
87,360 bp
87,370 bp
87,380 bp
Afraegle paniculata.bam
Afraegle-p:87,335
Total count: 504
A : 503 (100%, 231+, 272-)
C : 1 (0%, 1+, 0-)
G : 0
T : 0
Substitution of A base caused to premature stop codon

B)

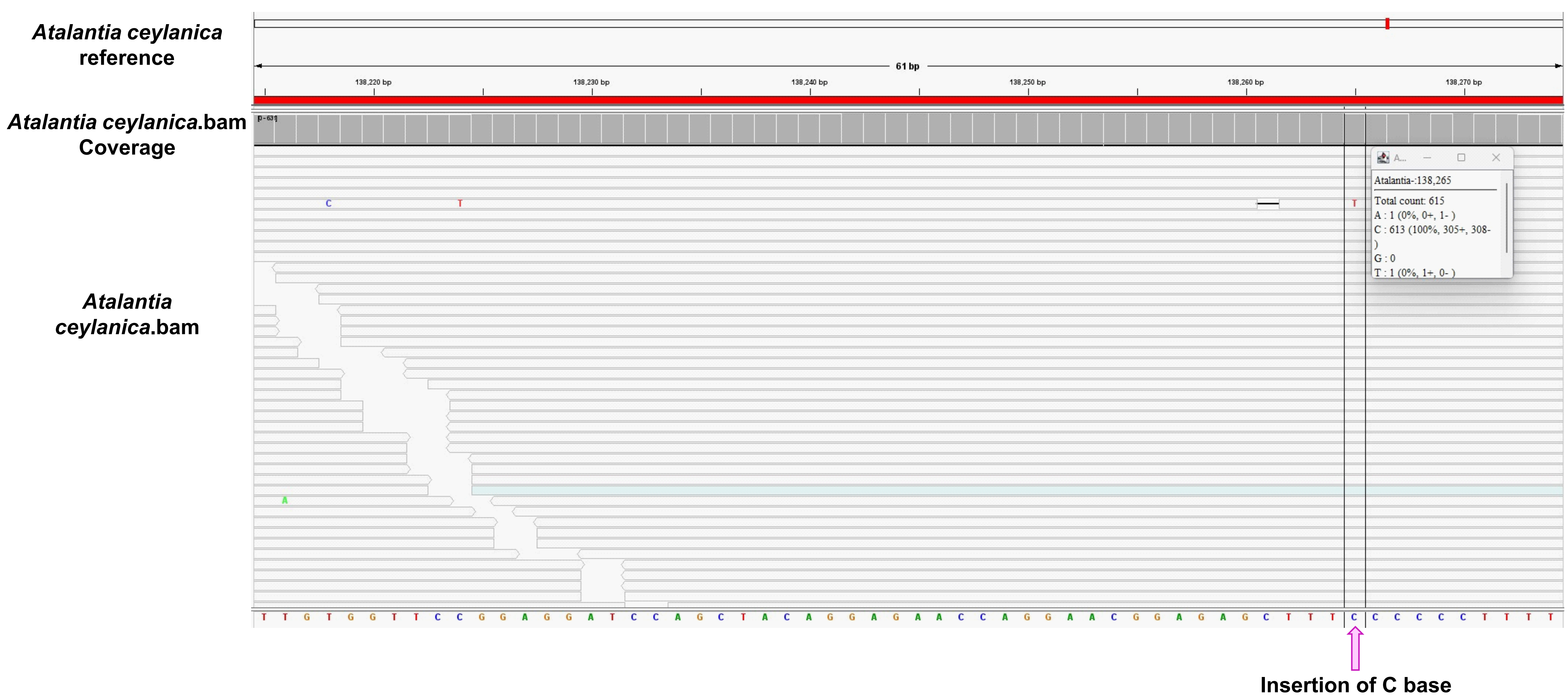
Atalantia ceylanica reference
61 bp
138,220 bp
138,230 bp
138,240 bp
138,250 bp
138,260 bp
138,270 bp
Atalantia ceylanica.bam Coverage
Atalantia ceylanica.bam
Atalantia-:138,265
Total count: 615
A : 1 (0%, 0+, 1-)
C : 613 (100%, 305+, 308-)
G : 0
T : 1 (0%, 1+, 0-)
Insertion of C base
