# Supplementary material for: Plastome evolution in Aurantioideae and plastome-based classification of Citrus
Source: PLoS One. 2026 Oct 7;21(10):e0349882. doi: 10.1371/journal.pone.0349882 (PMC13645046; doi:10.1371/journal.pone.0349882)
Supplement: Supplementary file 3 [file pone.0349882.s003.pdf]

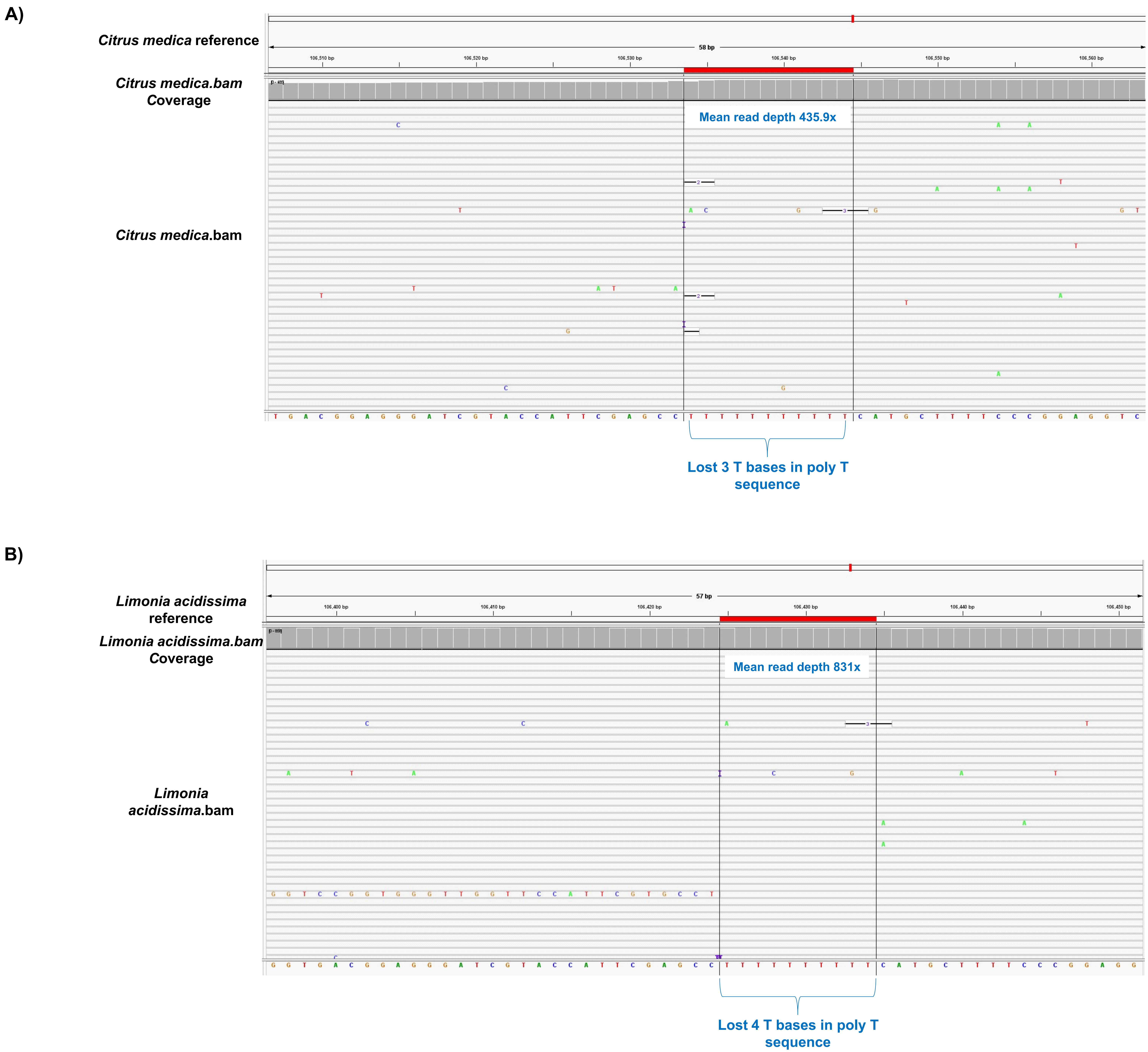
A)
Citrus medica reference
58 bp
106,510 bp
106,520 bp
106,530 bp
106,540 bp
106,550 bp
106,560 bp
Citrus medica.bam Coverage
Mean read depth 435.9x
Citrus medica.bam
Lost 3 T bases in poly T sequence
B)
Limonia acidissima reference
57 bp
106,400 bp
106,410 bp
106,420 bp
106,430 bp
106,440 bp
106,450 bp
Limonia acidissima.bam Coverage
Mean read depth 831x
Limonia acidissima.bam
Lost 4 T bases in poly T sequence
