# Supplementary material for: Plastome evolution in Aurantioideae and plastome-based classification of Citrus
Source: PLoS One. 2026 Oct 7;21(10):e0349882. doi: 10.1371/journal.pone.0349882 (PMC13645046; doi:10.1371/journal.pone.0349882)
Supplement: Supplementary file 4 [file pone.0349882.s004.pdf]

A)

A1)

```
M  T  K  K  N  A  S  I  P  L  R  Y  L  S  S  I  V  F  V  V
ATGACAAAAAAGAACGCATCCATTCCCCTTAGATATCTTTCATCTATAGTATTTGTAGTA

F  L  P  W  W  I  P  L  S  F  N  K  S  L  E  S  W  V  T  N
TTTTTGCCCTGGTGGATCCCTCTCTCATTTAATAAAAGTCTGGAATCCTGGGTTACTAAT

W  W  N  T  S  Q  S  E  T  F  L  N  G  I  Q  E  K  A  I  L
TGGTGGAATACTAGTCAATCCGAAACCTTTTTGAATGGTATTCAAGAAAAGGCTATTCTA

E  K  F  I  E  L  E  E  L  F  L  L  D  E  M  I  K  E  F  P
GAAAAATTCATAGAATTAGAGGAATTATTCCTCTTGGATGAAATGATAAAGGAATTTCCG

E  R  R  L  E  K  L  R  I  G  L  Q  K  E  T  I  Q  L  I  K
GAAAGACGTCTAGAAAAGCTTCGTATAGGGCTCCAGAAAGAAACAATCCAATTAATCAAG

M  H  D  E  D  H  I  H  T  I  F  H  F  S  T  N  T  I  C  F
ATGCATGATGAGGATCATATCCATACGATTTTTCACTTCTCGACAAATACAATCTGCTTC

V  I  L  S  G  Y  S  I  L  C  N  E  E  L  F  I  L  N  S  W
GTTATTCTAAGTGGTTATTCTATTCTGTGTAATGAAGAACTTTTTATTCTTAACTCTTGG

V  Q  E  F  L  Y  N  L  S  D  T  L  K  A  F  S  I  L  F  V
GTTCAAGAATTCCTATATAATTTAAGCGACACACTAAAAGCCTTTTCGATTCTTTTCGTA

T  D  L  C  I  G  F  H  S  P  R  G  W  E  L  L  I  D  Y  V
ACTGATTTATGTATCGGATTCCATTCACCCCGCGGTTGGGAACTACTGATTGACTATGTC

S  N  D  F  G  L  A  H  N  D  I  I  L  S  V  L  V  S  T  F
TCCAACGACTTTGGGCTTGCTCATAATGATATTATTCTATCTGTTCTTGTTTCCACTTTT

P  V  V  L  D  T  F  F  K  Y  W  L  F  S  Y  L  N  R  V  S
CCAGTCGTTCTAGATACATTTTTTAAATATTGGCTTTTTTCTTATTTAAATCGTGTATCT

P  S  L  V  V  I  Y  H  S  M  T  E  *
CCGTCACTTGTAGTGATTTATCATTCAATGACTGAGTGA
```

A2)

```
R  T  I  E  S  T  K  E  V  G  S  L  T  I  H  R  *  Q  N  D
CGAACTATAGAATCAACAAAAGAGGTGGGTTCATTAACAATTCACCGATGACAAAATGAC

K  K  K  K  N  A  S  S  P  L  R  Y  L  S  S  I  V  F  V  V
AAAAAAAAAAAGAACGCATCCAGTCCCCTTAGATATCTTTCATCTATAGTATTTGTAGTA

F  L  P  W  W  I  P  L  S  F  N  K  S  L  E  S  W  V  T  N
TTTTTGCCCTGGTGGATCCCTCTCTCATTTAATAAAAGTCTGGAATCCTGGGTTACTAAT

W  W  N  T  S  Q  S  E  T  F  L  N  E  I  Q  E  K  A  I  L
TGGTGGAATACTAGTCAATCCGAAACCTTTTTGAATGAGATTCAAGAAAAGGCTATTCTA

E  K  F  I  E  L  E  E  L  F  L  L  D  E  M  I  K  E  F  P
GAAAAATTCATAGAATTAGAGGAATTATTCCTCTTGGACGAAATGATAAAGGAATTTCCG

E  R  R  L  E  K  L  R  I  G  F  Q  K  E  T  I  Q  L  I  K
GAAAGACGTCTCGAAAAGCTTCGTATAGGGTTCCAGAAAGAAACAATCCAATTAATCAAG

M  Y  D  E  D  H  I  H  T  I  F  H  F  S  T  N  T  I  C  F
ATGTATGATGAGGATCATATCCATACGATTTTTCACTTTTCGACAAATACAATCTGCTTC

V  I  L  S  G  Y  S  I  L  C  N  K  E  L  F  I  L  N  S  W
GTTATTCTAAGTGGTTATTCTATTCTATGTAATAAAGAACTTTTTATTCTTAACTCTTGG

V  Q  E  F  L  Y  N  L  S  D  T  L  K  A  F  S  I  L  F  V
GTTCAAGAATTCCTATATAATTTAAGCGACACACTAAAAGCCTTTTCGATTCTTTTCGTA

T  D  L  C  I  G  F  H  S  P  R  G  W  E  L  L  I  G  Y  V
ACTGATTTATGTATCGGATTCCATTCACCCCGCGGTTGGGAACTACTGATTGGCTATGTC

S  N  D  F  G  L  A  H  N  D  I  I  L  S  V  L  V  S  T  F
TCCAACGACTTTGGGCTTGCTCATAATGATATTATTCTATCTGTTCTTGTTTCCACTTTT

P  V  V  L  D  T  F  F  K  Y  W  L  F  S  Y  L  N  R  V  S
CCAGTCGTTCTAGATACATTTTTTAAATATTGGCTTTTTTCTTATTTAAATCGTGTATCT

P  S  L  V  V  I  Y  H  S  M  T  E  *
CCGTCACTTGTAGTGATTTATCATTCAATGACTGAGTGA
```

B)

B1)

```
1    A  T  A  E  T  *  K  N  *  N  L  R  S  K  H  Y  V  W  M  V
1   C  Y  C  *  N  I  E  E  L  K  S  *  I  K  T  L  C  M  D  G
1   M  L  L  L  K  H  R  R  I  E  I  L  D  Q  N  T  M  Y  G  W
1   ATGCTACTGCTGAAACATAGAAGAATTGAAATCTTAGATCAAAACACTATGTATGGATGG

21   *  T  A  *  T  R  I  L  E  Q  R  T  T  R  A  I  T  N  Y  I
21  M  N  C  L  N  K  N  S  *  T  A  N  H  Q  S  Y  Y  *  L  H
21  Y  E  L  L  K  Q  E  F  L  N  S  E  P  P  E  L  L  L  T  T
61  TATGAACTGCTTAAACAAGAATTCTTGAACAGCGAACCACCAGAGCTATTACTAACTACA

41   K  K  F  P  L
41  Q  K  I  S  I
41  S  K  N  F  H  *
121 TCAAAAAATTTCCATTAA
```

B2)

```
1    E  M  K  I  G  F  M  *  Q  E  K  G  F  P  L  L  S  R  K  D
1   *  N  E  N  W  I  H  V  T  G  E  R  F  P  I  T  *  P  E  R
1   M  K  *  K  L  D  S  C  N  R  R  K  V  S  H  Y  L  A  G  K
1   ATGAAATGAAAATTGGATTCATGTAACAGGAGAAAGGTTTCCCATTACTTAGCCGGAAAG

21   M  W  P  *  N  R  N  *  V  E  E  N  *  L  G  G  R  V  V  E
21  Y  V  A  M  K  *  E  L  S  G  R  E  L  T  G  W  *  S  R  G
21  I  C  G  H  E  I  G  I  K  W  K  R  I  D  W  V  V  E  S  W
61  ATATGTGGCCATGAAATAGGAATTAAGTGGAAGAGAATTGACTGGGTGGTAGAGTCGTGG

41   T  P  V  S  S  I  F  W  T  L  A  P  W  N  T  T  A  E  T  *
41  N  T  S  F  F  H  I  L  D  L  S  S  M  E  H  Y  C  *  N  I
41  K  H  Q  F  L  P  Y  F  G  P  *  L  H  G  T  L  L  L  K  H
121 AAACACCAGTTTCTTCCATATTTTGGACCTTAGCTCCATGGAACACTACTGCTGAAACAT

61   K  N  G  N  L  R  S  K  H  Y  V  W  M  V  *  T  A  *  T  R
61  E  E  W  K  S  *  I  K  T  L  C  M  D  G  M  N  C  L  N  K
61  R  R  M  E  I  L  D  Q  N  T  M  Y  G  W  Y  E  L  L  K  Q
181 AGAAGAATGGAAATCTTAGATCAAAACACTATGTATGGATGGTATGAACTGCTTAAACAA

81   I  L  E  Q  R  T  T  R  G  Y  Y  *  L  H  Q  K  I  S  I
81  N  S  *  T  A  N  H  Q  R  L  L  L  T  T  S  K  N  F  H  *
81  E  F  L  N  S  E  P  P  E  A  I  T  N  Y  I  K  K  F  P  L
241 GAATTCTTGAACAGCGAACCACCAGAGGCTATTACTAACTACATCAAAAAATTTCCATTA
                              ↑
                        Insertion of G

301 A
301
301 T
```
