# Supplementary material for: Plastome evolution in Aurantioideae and plastome-based classification of Citrus
Source: PLoS One. 2026 Oct 7;21(10):e0349882. doi: 10.1371/journal.pone.0349882 (PMC13645046; doi:10.1371/journal.pone.0349882)
Supplement: Supplementary file 5 [file pone.0349882.s005.pdf]

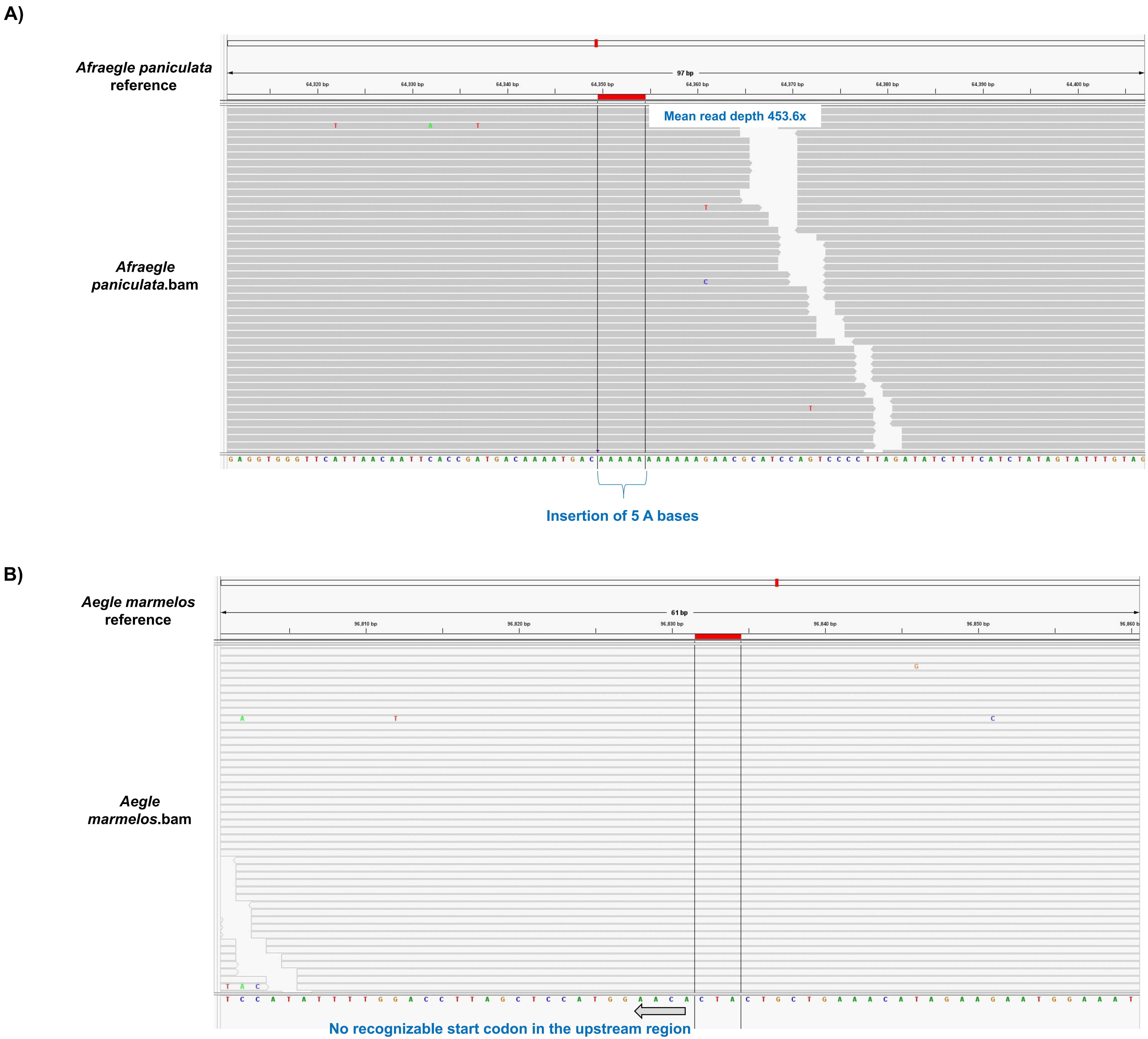
A)
Afraegle paniculata reference
97 bp
64,320 bp
64,330 bp
64,340 bp
64,350 bp
64,360 bp
64,370 bp
64,380 bp
64,390 bp
64,400 bp
Mean read depth 453.6x
Afraegle paniculata.bam
Insertion of 5 A bases
B)
Aegle marmelos reference
61 bp
96,810 bp
96,820 bp
96,830 bp
96,840 bp
96,850 bp
Aegle marmelos.bam
No recognizable start codon in the upstream region
