# Supplementary material for: Plastome evolution in Aurantioideae and plastome-based classification of Citrus
Source: PLoS One. 2026 Oct 7;21(10):e0349882. doi: 10.1371/journal.pone.0349882 (PMC13645046; doi:10.1371/journal.pone.0349882)
Supplement: Supplementary file 6 [file pone.0349882.s006.pdf]

A)

A1)

```
1    V  T  L  P  R  A  S  F  Q  K  N  T  P  S  G  G  F  A  G  T
1  C  H  V  T  E  G  L  V  S  K  K  Y  A  V  W  G  L  C  R  D
1 M  S  R  Y  R  G  P  R  F  K  K  I  R  R  L  G  A  L  P  G
1 ATGTCACGTTACCGAGGGCCTCGTTTCAAAAAAATACGCCGTCTGGGGGCTTTGCCGGGA
```

(180 bp)

```
61   R  T  T  I  T  *  I  C  S  Y  R  Q  K  S  Q  R  V  N  R  S
61 Q  N  D  N  Y  L  N  M  F  V  S  P  E  K  P  K  G  Q  P  V
61 T  E  R  Q  L  L  K  Y  V  R  I  A  R  K  A  K  G  S  T  G
181 ACAGAACGACAATTACTTAAATATGTTCGTATCGCCAGAAAAGCCAAAGGGTCAACCGGT
```

(60 bp)

```
101  F  N  Y  S  S  S  P  P  I  G  *  P  S  T  Y  F  S  *  W  S
101 L  Q  L  F  L  K  P  A  N  W  L  T  I  D  I  F  *  L  M  V
101 A  S  T  I  P  Q  A  R  Q  L  V  N  H  R  H  I  L  V  N  G
301 GCTTCAACTATTCCTCAAGCCCGCCAATTGGTTAACCATCGACATATTTTAGTTAATGGT
```

A2)

```
1    V  T  L  P  R  A  S  F  Q  K  N  T  P  S  G  G  F  A  G  T
1  C  H  V  T  E  G  L  V  S  K  K  Y  A  V  W  G  L  C  R  D
1 M  S  R  Y  R  G  P  R  F  K  K  I  R  R  L  G  A  L  P  G
1 ATGTCACGTTACCGAGGGCCTCGTTTCAAAAAAATACGCCGTCTGGGGGCTTTGCCGGGA
```

(180 bp)

```
61   R  T  T  I  T  *  Y  V  R  I  A  R  K  A  K  G  S  T  G  L
61 Q  N  D  N  Y  L  I  C  S  Y  R  Q  K  S  Q  R  V  N  R  S
61 T  E  R  Q  L  L  N  M  F  V  S  P  E  K  P  K  G  Q  P  V
181 ACAGAACGACAATTACTTAATATGTTCGTATCGCCAGAAAAGCCAAAGGGTCAACCGGTC
```

Deletion of A

(60 bp)

```
101  S  T  I  P  Q  A  R  Q  L  V  N  H  R  H  I  L  V  N  G  R
101 F  N  Y  S  S  S  P  P  I  G  *  P  S  T  Y  F  S  *  W  S
101 L  Q  L  F  L  K  P  A  N  W  L  T  I  D  I  F  *  L  M  V
301 CTTCAACTATTCCTCAAGCCCGCCAATTGGTTAACCATCGACATATTTTAGTTAATGGTC
```

B)

B1)

```
1    D  F  S  I  F  Y  T  R  *  S  S  I  L  M  H  E  D  N  Q  F
1  *  F  F  N  L  L  Y  *  V  I  *  Y  P  Y  A  *  R  *  S  I
1 M  I  F  Q  S  F  I  L  G  N  L  V  S  L  C  M  K  I  I  N
1 ATGATTTTTCAATCTTTTATACTAGGTAATCTAGTATCCTTATGCATGAAGATAATCAAT
```

(1,020 bp)

```
361  E  I  F  I  M  V  *  K  T  S  C  D  S  S  F  R  L  *  T  L
361 R  N  I  Y  Y  G  L  K  N  L  L  *  L  F  F  S  T  I  N  V
361 E  K  Y  L  L  W  F  E  K  P  L  V  T  L  L  F  D  Y  K  R
1081 GAGAAATATTTATTATGGTTTGAAAAACCTCTTGTGACTCTTCTTTTCGACTATAAACGT
```

(4,320 bp)

```
1821  G  P  Y  V  S  T  I  E  N  S  L
1821 G  S  I  C  I  H  D  *  K  F  V
1821 R  V  H  M  Y  P  R  L  K  I  R  *
5461 AGGGTCCATATGTATCCACGATTGAAAATTCGTTAA
```

B2)

```
1    D  F  S  I  F  Y  T  R  *  S  S  I  L  M  H  E  D  N  Q  F
1  *  F  F  N  L  L  Y  *  V  I  *  Y  P  Y  A  *  R  *  S  I
1 M  I  F  Q  S  F  I  L  G  N  L  V  S  L  C  M  K  I  I  N
1 ATGATTTTTCAATCTTTTATACTAGGTAATCTAGTATCCTTATGCATGAAGATAATCAAT
```

(1,020 bp)

```
361  E  I  L  L  W  F  E  K  P  L  V  T  L  L  F  D  Y  K  R  W
361 R  N  I  I  M  V  *  K  T  S  C  D  S  S  F  R  L  *  T  L
361 E  K  Y  Y  Y  G  L  K  N  L  L  *  L  F  F  S  T  I  N  V
1081 GAGAAATATTATTATGGTTTGAAAAACCTCTTGTGACTCTTCTTTTCGACTATAAACGTT
```

Deletion of T

(4,320 bp)

```
1821  V  H  M  Y  P  R  L  K  I  R  *
1821 G  P  Y  V  S  T  I  E  N  S  L
1821 G  S  I  C  I  H  D  *  K  F  V
5461 GGGTCCATATGTATCCACGATTGAAAATTCGTTAA
```
