# Supplementary material for: Plastome evolution in Aurantioideae and plastome-based classification of Citrus
Source: PLoS One. 2026 Oct 7;21(10):e0349882. doi: 10.1371/journal.pone.0349882 (PMC13645046; doi:10.1371/journal.pone.0349882)
Supplement: Supplementary file 7 [file pone.0349882.s007.pdf]

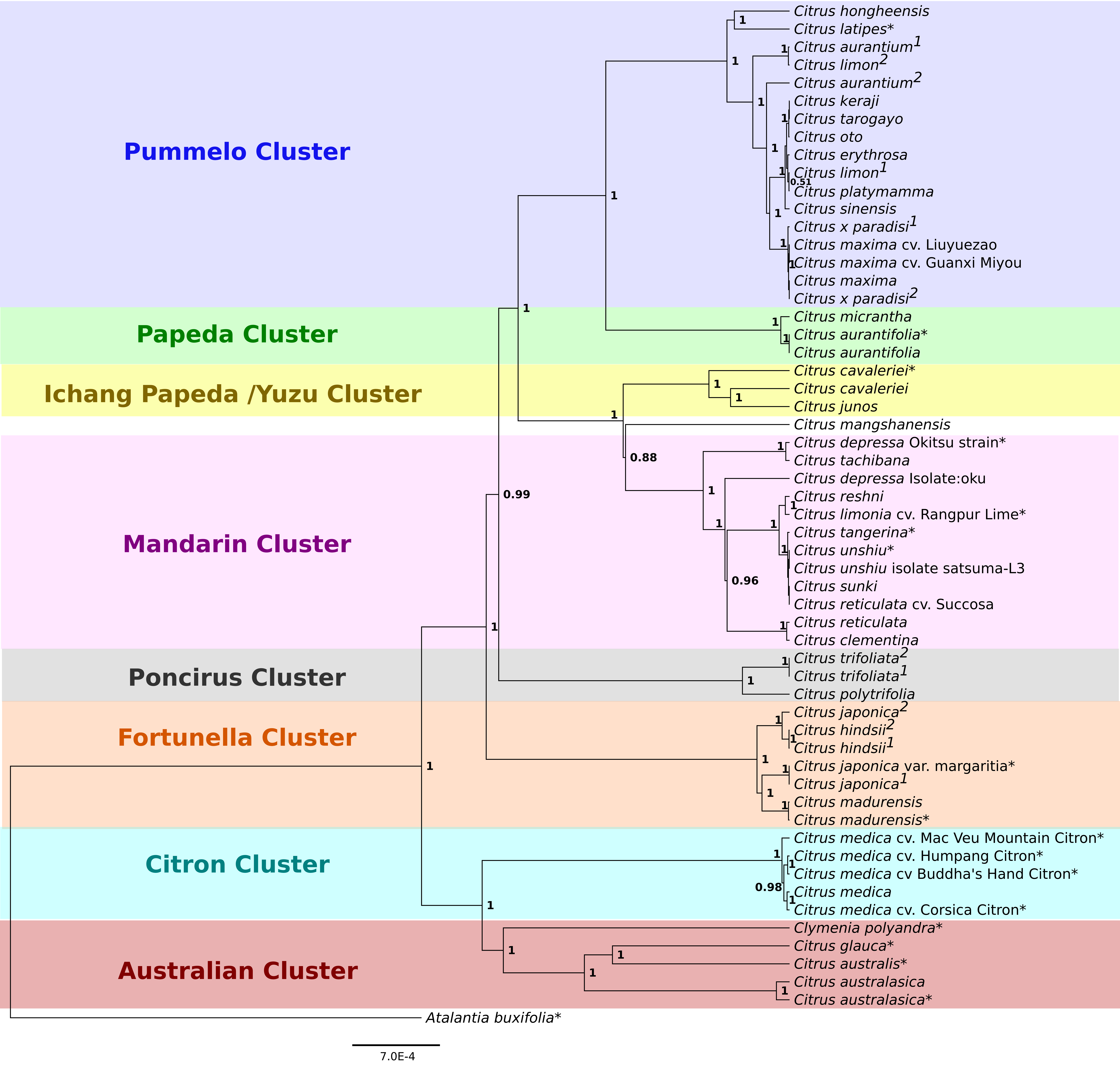
Pummelo Cluster
Papeda Cluster
Ichang Papeda /Yuzu Cluster
Mandarin Cluster
Poncirus Cluster
Fortunella Cluster
Citron Cluster
Australian Cluster
Citrus hongheensis
Citrus latipes*
Citrus aurantium1
Citrus limon2
Citrus aurantium2
Citrus keraji
Citrus tarogayo
Citrus oto
Citrus erythrosa
Citrus limon1
Citrus platymamma
Citrus sinensis
Citrus x paradisi1
Citrus maxima cv. Liuyuezao
Citrus maxima cv. Guanxi Miyou
Citrus maxima
Citrus x paradisi2
Citrus micrantha
Citrus aurantifolia*
Citrus aurantifolia
Citrus cavaleriei*
Citrus cavaleriei
Citrus junos
Citrus mangshanensis
Citrus depressa Okitsu strain*
Citrus tachibana
Citrus depressa Isolate:oku
Citrus reshni
Citrus limonia cv. Rangpur Lime*
Citrus tangerina*
Citrus unshiu*
Citrus unshiu isolate satsuma-L3
Citrus sunki
Citrus reticulata cv. Succosa
Citrus reticulata
Citrus clementina
Citrus trifoliata2
Citrus trifoliata1
Citrus polytrifolia
Citrus japonica2
Citrus hindsii2
Citrus hindsii1
Citrus japonica var. margaritia*
Citrus japonica1
Citrus madurensis
Citrus madurensis*
Citrus medica cv. Mac Veu Mountain Citron*
Citrus medica cv. Humpang Citron*
Citrus medica cv Buddha's Hand Citron*
Citrus medica
Citrus medica cv. Corsica Citron*
Clymenia polyandra*
Citrus glauca*
Citrus australis*
Citrus australasica
Citrus australasica*
Atalantia buxifolia*
0.51
0.88
0.99
0.96
0.98
7.0E-4
