# Supplementary material for: Plastome evolution in Aurantioideae and plastome-based classification of Citrus
Source: PLoS One. 2026 Oct 7;21(10):e0349882. doi: 10.1371/journal.pone.0349882 (PMC13645046; doi:10.1371/journal.pone.0349882)
Supplement: Supplementary file 8 [file pone.0349882.s011.docx]

Dear Dr. Siler and Reviewers,

We sincerely thank the Academic Editor and both reviewers for their careful and constructive evaluation of our manuscript entitled “Plastome evolution in Aurantioideae and plastome-based classification of *Citrus*.” Their insightful comments and suggestions have helped us substantially improve the scientific rigor, clarity, and organization of the manuscript. We have carefully addressed all the points raised and thoroughly revised the manuscript. Line numbers in our responses refer to the clean revised manuscript (Revised_Manuscript.docx); line numbers in the reproduced reviewer comments refer to the original submission. The principal revisions are summarized below:

For the present repository update, the source-data and Supplementary Dataset descriptions have been clarified, and the line references below have been checked against the updated clean manuscript. The scientific responses from the previous revision are retained; no further scientific analyses or changes to the results were made for this repository update.

1. The manuscript has been reorganized according to the reviewer’s recommendation. The Results now proceed logically from general plastome characterization to comparative genomic, phylogenomic, and divergence-time analyses. The main and supplementary figures and tables have also been renumbered accordingly.
2. The Introduction, Materials and Methods, Results, and Discussion have been extensively revised in response to the comments from the Academic Editor and reviewers.
3. The Results section has been thoroughly revised to remove interpretive statements more appropriate for the Discussion.
4. The divergence-time analysis was repeated using MCMCtree in response to the reviewer’s comments. The Materials and Methods, Results, Discussion, and associated figure have been revised to reflect the new analysis.
5. A new Results subsection entitled “Aurantioideae plastomes were highly conserved with localized genomic and sequence variation” has been added to provide a general characterization of the analyzed plastomes.
6. Supplementary Tables S2 and S3 and Supplementary Figures S1, S2, S3, and S5 have been added to provide further methodological details and supporting evidence requested by the reviewers.
7. Additional references recommended by the reviewers have been incorporated, together with other relevant literature needed to support the revised analyses and discussion.

We believe that these revisions have substantially strengthened the manuscript by improving its organization, methodological rigor, precision, and overall scientific value.

We are grateful for the Academic Editor’s and reviewers’ thoughtful guidance and the time and effort devoted to evaluating our work. We sincerely hope that the revised submission meets your expectations and is considered favorably for publication.

# **Response to the Academic Editor’s major points**

We sincerely thank the Academic Editor for summarizing the principal concerns raised during the review. We have carefully considered these issues, conducted additional analyses, and substantially revised the manuscript. Concise responses are provided below; further details are presented in our point-by-point responses to the respective reviewers.

# 1. No new raw data were generated specifically for the analysis reported in this manuscript (Reviewer #2) but already published sequences and publicly available data were only used.

We agree and have clarified the origin of all data throughout the revised manuscript. No new raw sequencing data were generated specifically for this study. The analysis included 103 plastome accessions, comprising 88 Aurantioideae accessions and 15 outgroups. Of the Aurantioideae plastomes, 28 originated from our previous study, 15 were previously assembled from publicly available raw reads, and the remainder were publicly available complete plastome sequences. The Materials and Methods and Supplementary Table S1 have been revised to report these sources transparently. Further details are provided in our response to Reviewer #2.

# 2. For the phylogenetic analyses, the authors failed to cite and compare their findings with major literature findings.

We have substantially revised the phylogenetic Discussion to compare our findings with major previous studies of *Citrus* and related taxa. In particular, we now discuss the eight plastome-based clusters in relation to earlier marker-based and whole-plastome phylogenetic studies, while acknowledging differences in taxon sampling, molecular datasets, and analytical methods. We also clarify the limitations of plastome phylogenies arising from introgression, hybridization, and plastome capture. Relevant references have been added. A detailed explanation is provided in our responses to the reviewers.

# 3. Results on structural variation and pseudogenization - it appears that at least part of them has already been described in another paper by the authors (Reviewer #2).

We appreciate the opportunity to clarify this issue. The approximately 22-kb inversion was previously reported and is neither analyzed nor presented as a finding in the present study. We cite the earlier publication only to acknowledge that previously reported structural observation and to clearly distinguish it from the scope of the current work. The present study focuses specifically on variation at the LSC–IR boundaries and on broadly distributed and lineage-specific patterns of sequence-based pseudogenization. Where raw sequencing data were available, the mutations associated with pseudogenization were further validated by examining read coverage. Further clarification is provided in our detailed response to Reviewer #2.

# 4. The common genome characterization was not reported (Reviewer #1).

We have added a new Results subsection entitled “Aurantioideae plastomes were highly conserved with localized genomic and sequence variation.” This subsection reports plastome size, GC content, and unique protein-coding, tRNA, rRNA, and total gene numbers across the 88 Aurantioideae accessions. Supplementary Table S3 has also been added to provide accession-level plastome characteristics. Please see our detailed response to Reviewer #1.

# 5. Divergence-time analysis - RelTime-ML in MEGA11 with a single calibration point is considered insufficient (Reviewer #2).

We agree that the original analysis required substantial improvement. We therefore repeated the divergence-time analysis using the approximate-likelihood method implemented in MCMCtree within PAML v4.10.10. The analysis used a fixed IQ-TREE topology for 47 taxa, rooted with *Ailanthus altissima*, and incorporated four temporal constraints based on published fossil assessments and divergence estimates. We also performed a calibration-sensitivity analysis by replacing the 47.8-Ma minimum constraint for stem *Zanthoxylum* with a 66-Ma minimum constraint based on *Rutaspermum biornatum*. The Materials and Methods, Results, Discussion, and divergence-time figure have been revised accordingly. Full methodological details and convergence assessments are provided in our response to Reviewer #2.

# 6. Essential information about the sequencing data used for plastome assembly was not provided (Reviewer #2).

We have expanded the Materials and Methods to describe the sources of the sequencing data, library preparation, sequencing platform, read configuration, quality filtering, assembly, annotation, and validation procedures. We have also added Supplementary Table S2, which reports the filtered-read and plastome-mapping statistics for all raw-read datasets included in the study. Further details are provided in our response to Reviewer #2.

# 7. Frequent lack of appropriate literature support for the reported statements, and high rate of self-citation (Reviewer #2).

We have carefully reviewed and revised the citations throughout the manuscript. Statements that previously lacked adequate support are now supported by relevant independent literature, including the references suggested by the reviewers. The revised manuscript contains 61 references, of which eight are publications authored or coauthored by the present authors. Thus, the revised reference list is predominantly based on independent literature. Further details are provided in our point-by-point response to Reviewer #2.

# Regarding fundamental methodology problems this study suffers from, I advise the authors to meticulously study the reviewers' comments, try to perform additional analyses, and considerably transform the manuscript, thus raising its overall quality. Please provide point-by-point reply to reviewers' comments.

We carefully examined every comment from the reviewers and prepared a complete point-by-point response. In addition to extensive revisions of the Introduction, Materials and Methods, Results, and Discussion, we reorganized the manuscript, added general plastome characterization, provided additional sequencing and mapping information, clarified the relationship between the present and previously published findings, expanded the literature-based comparisons, and completely repeated the divergence-time analysis using MCMCtree with multiple temporal constraints and a calibration-sensitivity analysis. We believe that these revisions and additional analyses have addressed the fundamental methodological concerns and substantially improved the rigor, transparency, and overall quality of the manuscript.

# **Reviewer 1**

# The manuscript describes the characterization of the complete plastid genome sequences of members of Aurantioideae.

Thank you very much for carefully reviewing our manuscript and for providing constructive comments and suggestions that helped improve the quality and clarity of our work. We have carefully addressed all of your comments and revised the manuscript accordingly.

# My comments are as follows:

# 1) A mixture used of "chloroplast genome" and "plastid genome" was used in abstract and main text. Please be consistent. Based on the title, "plastid genome" and "plastome" are more preferable.

Thank you for this comment. We have revised the manuscript to use the term “plastome” consistently throughout the text and have replaced “chloroplast genome” in our text; original reference titles have been retained.

# 2) It could be just me, but the arrangement of the workflow is weird. The authors should be characterizing the genome first, then analyzing the genome, perform comparative analysis, then phylogenomics analysis and divergence time analysis. However, the authors started with phylogenomic and divergence time, followed by genome comparative. The common genome characterization was not reported. There is no information on the number of genes annotated, etc. It could be "useless" in the author's perspective, but the number of genes could also indicate some genome evolutionary pattern.

Thank you for this valuable comment. We agree that presenting the basic plastome characteristics before the comparative, phylogenomic, and divergence-time analyses provides a clearer and more logical flow. Accordingly, we have reorganized the manuscript following the suggested sequence of analyses.

We have also added a general characterization of the Aurantioideae plastomes, including plastome size, GC content, and gene number, with the corresponding data provided in Supplementary Table S3. Across the 88 Aurantioideae accessions analyzed, all plastomes contained 30 unique tRNA genes and four unique rRNA genes. Most accessions (85 of 88) contained 83 unique protein-coding genes, resulting in a total of 117 unique genes per plastome.

Although variation in gene number can potentially provide insights into genome evolution, our analysis showed that gene content was highly conserved across Aurantioideae. Therefore, no clear broad-scale genome evolutionary pattern was evident based on gene number alone. The limited differences observed in a small number of accessions were associated with lineage-specific gene loss or pseudogenization events, which are examined in greater detail in the subsequent comparative analyses.

The following text has been added to the revised manuscript:

**Lines 220–229:**

**Aurantioideae plastomes were highly conserved with localized genomic and sequence variation**

A total of 103 plastome accessions were analyzed, comprising 88 Aurantioideae accessions and 15 outgroup taxa (Supplementary Table S3). Among the Aurantioideae accessions, plastome size ranged from 158,779 bp in *Naringi crenulata* to 161,172 bp in *Clausena excavata*. Overall GC content varied by only 0.39 percentage points, ranging from 38.28% in *C. excavata* to 38.67% in *Clausena harmandiana* and *Clausena lansium*. Gene content was also highly uniform across the subfamily. All 88 Aurantioideae plastomes contained 30 unique tRNA genes and four unique rRNA genes. Most accessions (85 of 88) contained 83 unique protein-coding genes, corresponding to a total of 117 unique genes per plastome (Supplementary Table S3).

# 3) The reporting of pseudogenized genes is not practical, in my opinion. The outcome is bioinformatics-based. In a strict manner, a gene can be regarded as pseudo for good when it is proven of its functionality through wet lab. Wet lab was not performed here. The annotation of pseudo genes is just at predicted-level. Having a large amount of discussion on the predicted-to-be-pseudogenized genes seemed to be illogical.

Thank you for this important comment. We agree that experimental validation would be required to conclusively determine the functional status, transcription, or translation of the genes identified as pseudogenized. In the present study, however, pseudogenization refers specifically to sequence-level evidence obtained through comparative plastome analysis rather than to experimentally demonstrated loss of gene function.

In comparative plastome genomics, putative pseudogenization is commonly inferred from disruption of the expected coding sequence, including frameshift mutations, premature stop codons, loss of recognizable start codons, substantial deletions, or truncation of the coding region. Such sequence-based identification has been widely used in previous plastome studies to investigate patterns of gene degeneration and genome evolution. We therefore consider the identification of putative pseudogenization from sequence-level evidence to be consistent with established comparative genomic practice.

In our study, the inferred events were identified through comparison with intact homologous sequences in closely related taxa and were associated with specific disruptive mutations affecting the corresponding coding regions. In addition, where raw sequencing data were available, these mutations were examined using IGV to confirm read coverage and support for the sequence changes underlying the inferred pseudogenization. We recognize that such validation confirms the presence of the sequence disruption but does not, by itself, demonstrate complete loss of biological function.

To address your concern, we have revised the Discussion to distinguish more clearly between sequence-supported putative pseudogenization and experimentally demonstrated functional loss. We now explicitly refer to these events as putative pseudogenization or sequence-level evidence of pseudogenization where appropriate. We have also clarified that possible mechanisms, including relaxed selective constraint, nuclear functional replacement, or lineage-specific structural changes, are evolutionary interpretations rather than experimentally demonstrated processes.

Similarly, the discussion of lineage-specific cases such as *accD*, *ycf1*, *cemA*, *ycf15*, and *rps4* has been revised so that any possible functional implications are presented explicitly as hypotheses requiring further investigation. For example, the possible relationship between *accD* and *ycf1*, potential compensation of *cemA* function by a nuclear-encoded protein, or possible nuclear transfer of a functional gene copy are now discussed cautiously and are not presented as conclusions supported directly by the plastome data. We have also added an explicit statement that transcriptomic, nuclear-genome, and functional analyses would be required to determine whether the observed sequence disruptions result in true functional loss, compensation, or gene transfer.

For these reasons, we have retained the pseudogenization analysis because the sequence-level patterns remain relevant to the comparative and evolutionary objectives of the present study. However, the associated Discussion has been revised to avoid overinterpreting the predicted pseudogenes and to clearly separate sequence-based evidence of gene degeneration from experimentally demonstrated gene function.

**Lines 371–406:** The distribution of putative pseudogenization events further indicates that localized plastome evolution in Aurantioideae has involved changes at multiple loci. Recurrent sequence disruptions affecting *rpl22*, *ycf68*, and *orf56* suggest that coding-sequence degeneration has occurred through different mutational routes. Comparable patterns have been reported elsewhere in angiosperms. For example, *infA* has been independently lost or pseudogenized multiple times, in several cases following functional transfer to the nucleus [48], while repeated plastome loss of *rpl22* has been associated with nuclear functional replacement [49,50]. By contrast, *ycf68* appears to be nonfunctional or weakly constrained in many angiosperms [51], emphasizing the need for caution when interpreting sequence disruptions in genes of uncertain functional status. The patterns detected here may therefore reflect several evolutionary processes, including relaxed selective constraint, lineage-specific structural changes, or, for some genes, functional replacement outside the plastome. However, these mechanisms cannot be distinguished using plastome sequences alone. Recurrent disruptions also caution against treating individual putative pseudogenization events as definitive phylogenetic characters.

In addition to these more broadly distributed patterns, several lineage-specific sequence disruptions warrant further investigation. In *Afraegle paniculata*, the putative pseudogenization of *cemA* [52] raises questions about whether its function has been retained or compensated through another mechanism. Possible explanations include compensation involving the nuclear-encoded *DLDG1* [53] or functional transfer of *cemA* to the nucleus followed by acquisition of a plastid-targeting transit peptide. Neither possibility, however, can be established from the present data. The putative pseudogenization of *ycf15* in *Aegle marmelos* is best interpreted primarily as a lineage-specific sequence feature because the functional status of this gene remains uncertain in many angiosperms [16]. The disruption observed in *rps4* in *Citrus sinensis* is also noteworthy because the sweet orange plastome has been widely used as a reference in citrus plastome studies, yet this feature was not explicitly discussed in the original plastome report [54].

In *Atalantia kwangtungensis*, we recovered sequence-level evidence consistent with the previously reported putative pseudogenization of *accD* [55] and additionally identified disruptive changes in *ycf1*. The *accD* gene encodes a subunit of acetyl-CoA carboxylase [56,57], whereas *ycf1* has been proposed to encode a component of the plastid protein translocon [58]. Because co-evolution between *accD* and *ycf1* has previously been suggested [58], the occurrence of disruptive changes in both genes within the same lineage may warrant further functional investigation. Their co-occurrence alone, however, does not demonstrate a functional relationship or a shared mechanism of degeneration. Collectively, the lineage-specific changes identified here provide sequence-level evidence of putative pseudogenization. Raw-read support for the underlying mutations strengthens confidence in the sequence observations but does not establish loss of gene function. Transcriptomic, nuclear-genome, and functional analyses will be required to determine whether these changes are associated with functional loss, compensation, or gene transfer.

# 4) The annotation of genes is based on the online software GeSeq. Based on my understanding, the latest version (which is not new), does not commonly annotates *orfs, ycf15, ycf68* for most angiosperm. Therefore, it struck my mind that an old/outdated annotated GenBank reference species was used for annotation, which could be inaccurate. Although gene annotations are predictive; GeSeq has been updated over time to provide an accurate annotation for plastid genome, in my opinion.

Thank you for this important comment. We agree that automated plastome annotation can be influenced by the reference sequences used and that particular features, including *orf56*, *ycf15*, and *ycf68*, require careful verification. We would like to clarify that the plastomes in this study were annotated using GeSeq v2.03, and the automated annotations were not accepted without further examination.

In our dataset, GeSeq initially identified *orf56*, *ycf15*, and *ycf68* in the analyzed plastomes. However, because these genes can be inconsistently annotated among publicly available plastome sequences, we specifically examined these regions during manual curation. Their annotations were verified using BLAST searches against homologous sequences and by examining the corresponding nucleotide sequences, translated amino acid sequences, and reading frames. Where necessary, the automated annotations were manually corrected based on these sequence-level comparisons.

Therefore, the final annotations used in our comparative analyses were not based solely on the automated GeSeq output or on annotations transferred directly from potentially outdated GenBank reference sequences. Instead, GeSeq was used for the initial annotation, followed by manual verification and curation, with particular attention to *orf56*, *ycf15*, and *ycf68*. We believe that this procedure minimizes potential annotation errors associated with automated prediction and provides a more reliable basis for the subsequent comparative analyses.

To clarify the annotation procedure, we have revised the Materials and Methods section by specifying the GeSeq version and explicitly stating that all annotations were manually inspected and corrected where necessary.

**Lines 133–137:** Plastome annotation was performed using GeSeq v2.03 [23], with protein-coding sequences predicted using Chloë v0.1.0, tRNA genes identified using tRNAscan-SE v2.0.7 [24], and rRNA genes identified using HMMER. Following automated annotation, all plastome annotations were manually inspected and corrected where necessary to ensure annotation accuracy and consistency. Final circular plastome maps were generated using OGDRAW v1.3.1 [25].

# 5) I have no idea how many new plastomes were reported in this study. I believe that most of the information can be presented in the Supplementary material, yet, it is still essential to be written out in the main text.

Thank you for this comment. We agree that the origin of the plastome datasets used in this study should be clearly stated in the main text, in addition to the detailed information provided in the Supplementary Tables. We have therefore revised both the main text and the Supplementary Tables to clearly indicate which plastomes originated from our previous study and which were obtained from publicly available data.

No new plastomes were generated specifically for the present study. A total of 103 plastome accessions were analyzed, comprising 88 Aurantioideae accessions and 15 outgroup taxa. Among the 88 Aurantioideae accessions, 28 plastomes were generated in our previous study. In addition, publicly available raw sequencing data for 15 accessions had been retrieved and de novo assembled as part of the dataset used in our previous study. The remaining Aurantioideae accessions were obtained as complete plastome sequences from public databases. The outgroup dataset consisted of 14 Rutaceae taxa outside Aurantioideae and *Ailanthus altissima*, which was used as the external outgroup.

Accordingly, the present study combines plastome data generated in our previous work with publicly available raw sequencing data and publicly available complete plastome sequences. We have revised the main text to make the composition and origin of the dataset explicit and have provided detailed accession and source information for all samples in the Supplementary Tables.

In Materials and Methods,

**Lines 99–106**: The present study investigated plastome evolution within the subfamily Aurantioideae of the family Rutaceae. A total of 103 plastome accessions were analyzed, comprising 88 Aurantioideae accessions and 15 outgroup taxa. The dataset included 29 accessions for which sequencing data had been generated in our previous studies [3,9], 15 accessions represented by publicly available raw sequencing datasets, and 59 accessions represented by publicly available complete plastome sequences. Thus, no new sequencing data were generated specifically for the present study. A complete list of the taxa, data sources, and corresponding sequence accession numbers is provided in Supplementary Table S1.

# 6) In most genome comparative analysis at a subfamily level, performing MAUVE could be more useful in providing information of the genome structure evolution at the genera level.

Thank you for this valuable suggestion. We agree that MAUVE is a useful approach for investigating large-scale genome structural variation, including rearrangements, inversions, and locally collinear blocks. However, the primary objective of our comparative plastome analysis was to evaluate patterns of sequence conservation and localized divergence among representative Aurantioideae genera rather than to reconstruct the history of large-scale structural rearrangements.

To strengthen the comparative analysis, we therefore performed whole-plastome comparisons using mVISTA with representative taxa from major genera of Aurantioideae. This analysis enabled us to evaluate the relative conservation of coding and non-coding regions, identify regions showing localized sequence divergence, and assess the overall similarity of plastome sequences across the subfamily. We have not interpreted the mVISTA analysis as a formal test of genome rearrangements.

The mVISTA analysis and its corresponding results have now been added to the Materials and Methods, Results and Discussion sections, respectively, as follows.

In Materials and Method section,

**Lines 147–154:** To assess sequence conservation and divergence among representative Aurantioideae plastomes, whole-plastome sequence comparisons were performed using mVISTA [26] with the Shuffle-LAGAN alignment mode [27], with the Citrus sinensis plastome used as the reference. Representative taxa included Aegle marmelos, Atalantia bilocularis, A. ceylanica, A. roxburghiana, Citropsis gabunensis, Clausena anisata, Citrus glauca, Limonia acidissima, Glycosmis citrifolia, Naringi crenulata, Murraya caloxylon, Micromelum minutum, Murraya koenigii, Murraya paniculata, Paramignya lobata, Swinglea glutinosa, and Triphasia trifolia. The resulting alignments were examined to evaluate patterns of sequence conservation and divergence across the plastomes.

In Results section,

**Lines 230–239:** Whole-plastome sequence conservation was further evaluated among representative Aurantioideae taxa using mVISTA, with *Citrus sinensis* as the reference plastome (S1 Fig). The comparison included representatives from multiple genera across the subfamily, including *Aegle*, *Atalantia*, *Citropsis*, *Clausena*, *Citrus*, *Glycosmis*, *Micromelum*, *Murraya*, *Naringi*, *Paramignya*, *Swinglea*, and *Triphasia*. Coding regions were generally more conserved than non-coding regions, whereas greater sequence divergence was observed mainly in intergenic and other non-coding regions (S1 Fig). The inverted-repeat regions, including the regions containing the rRNA genes, were highly conserved across the sampled taxa. In contrast, localized reductions in sequence similarity were evident at several positions within the large single-copy and small single-copy regions. Despite these localized differences, the overall alignment profiles were highly similar among the representative plastomes (S1 Fig).

In Discussion section,

**Lines 353–359:** The conserved organization and largely uniform gene content observed among Aurantioideae plastomes indicate that substantial lineage diversification has occurred without extensive changes in their overall genomic architecture. Nevertheless, this conservation does not imply structural or functional uniformity at individual loci. Comparative analyses revealed boundary-associated modifications and disruptive sequence changes that were not apparent from overall plastome size or gene counts alone. Consequently, general plastome characterization and detailed comparisons of individual regions provide complementary perspectives on plastome evolution within the subfamily.

# 7) The results section is written as if it is a discussion section. For example: Line 169 and 173. Please check throughout.

Thank you for this valuable comment. We have carefully reviewed the entire Results section and revised it throughout to ensure that it focuses only on the direct presentation of our findings. Interpretative or discussion-oriented statements, including those previously present around Lines 169 and 173, have been removed or relocated where appropriate. The revised Results section now presents the findings more objectively and distinctly from the Discussion.

# 8) *Clymenia polyandra* has already been synonymized to *Citrus polyandra*, according to POWO. (<https://powo.science.kew.org/taxon/urn:lsid:ipni.org:names:772184-1>). Please fact check.

Thank you for pointing this out. We rechecked the taxonomic status of this species and confirmed that *Clymenia polyandra* is treated as a synonym of *Citrus polyandra* in Plants of the World Online (POWO). However, throughout this study, species names were standardized according to the taxonomy database adopted for our dataset in order to maintain consistent nomenclature across all analyzed accessions. In that database, the current name is listed as Clymenia polyandra. Therefore, rather than applying nomenclature from different taxonomic sources selectively, we have retained Clymenia polyandra in the manuscript to ensure consistency with the taxonomic framework used for all other species in the study.

We have revised the Discussion to explicitly acknowledge this synonymy and to explain that the placement of *Clymenia polyandra* within the true citrus group is consistent with previous phylogenetic evidence supporting an expanded circumscription of *Citrus* (Bayer et al., 2009).

**Lines 410–415:** Clymenia polyandra was sister to the sampled Australian species, with this assemblage sister to the Citron cluster. Its position within the true citrus group agrees with the expanded circumscription of Citrus supported by Bayer et al. (2009) [12]. Although the name Clymenia polyandra is retained here following the NCBI taxonomy database, Plants of the World Online (POWO) recognizes it as a synonym of Citrus polyandra; its inclusion within Citrus therefore accords with established phylogenetic and taxonomic treatments.

# **Reviewer 2**

# Witharana and colleagues investigated plastome evolution in the subfamily Aurantioideae (Rutaceae), focusing on phylogenetic classification of Citrus, divergence-time estimation, structural variation at LSC-IR boundaries, and pseudogenization patterns. This manuscript potentially presents some interesting results, but in its current form, in my opinion, it is far from being suitable for publication in a scientific journal such as PLOS One, both in terms of presentation and scientific content. I would suggest considering whether the current content might be better suited as a short communication or as a "resource paper".

Thank you very much for your careful evaluation of our manuscript and for the time and effort you devoted to providing detailed comments and suggestions. We appreciate your concerns regarding both the presentation and scientific content of the original manuscript.

In response to your comments, we have extensively revised the manuscript to improve its organization, clarity, methodological description, and presentation of the results.

# The main issues are outlined below.

# (1) Very limited novelty of the reported results.

Thank you for this comment. We recognize that the individual analytical approaches used in this study are established and that some of the phylogenetic relationships identified here have been reported previously. However, we believe that the scientific contribution of the present study should be considered at the level of its overall scope and integration rather than solely in terms of the novelty of each individual analysis. To our knowledge, a plastome-based assessment of Aurantioideae combining general plastome characterization, structural variation at the LSC–IR boundaries, recurrent and lineage-specific putative pseudogenization, whole-plastome phylogenetics, and divergence-time estimation across a taxonomically broad dataset has not previously been conducted at this scale.

The present study therefore does not aim to introduce a fundamentally new analytical method. Rather, it provides an updated and substantially expanded assessment of plastome evolution across Aurantioideae. By integrating multiple complementary analyses within a common phylogenetic framework, the study allows broadly conserved features, recurrent evolutionary changes, and lineage-specific events to be evaluated together. We believe that this comprehensive perspective is scientifically important because it consolidates previously fragmented observations, places them in a broader evolutionary and temporal context, and provides an updated comparative framework for future studies of Aurantioideae systematics and plastome evolution.

We would also like to emphasize that comprehensive reassessments based on established molecular approaches can have substantial and lasting value even when methodological novelty is limited. In 2013, we published in PLOS ONE a broad *matK*-based phylogenetic analysis of Aurantioideae that similarly emphasized comprehensive taxonomic sampling and scientific soundness rather than methodological novelty. According to Google Scholar, that study had been cited 154 times as of September 12, 2026. We believe that its continued use illustrates the value of comprehensive comparative studies that provide a reliable framework for subsequent research.

A screenshot of the Google Scholar record is provided below as supporting evidence.

In this respect, the present study can be viewed, in part, as a substantial update and extension of that earlier work from a single plastid marker to whole-plastome data and from phylogenetic analysis alone to a broader examination of plastome evolution. The expanded taxonomic sampling, whole-plastome sequence information, structural comparisons, putative pseudogenization analyses, and revised divergence-time estimation together provide a considerably richer evolutionary framework than was previously available. Although we therefore acknowledge that the novelty of the individual analytical methods is limited, we consider the scale, integration, and updated evolutionary synthesis of the present study to represent a meaningful scientific contribution.

# Lines 65-74: I appreciate the authors' honesty in explicitly reporting these details, but this paragraph discussing the prior bioRxiv preprint and subsequent publication history reads more like a personal narrative of the authors' publication trajectory than a standard scientific introduction. Phrases detailing which analyses were "included" or "not included" in subsequent papers, and explanations of why certain findings were omitted during peer review, are better suited to a thesis preface or personal communication than to a journal article. Scientific papers should stand on their own merit and focus on the science, not the history of how the manuscript came to be. Most importantly, the described publication history makes it clear why the current manuscript reports only some of the analyses that could have been performed with these data: this

# manuscript essentially presents some “leftover” analyses from the original preprint that were not developed further in the subsequent publications. This, in itself, is not necessarily a problem; however, as a result, the current manuscript does appear rather “poor” as a standalone research article, in addition to presenting problematic and unclear aspects in the way the dataset used is described, given that it derives from other studies.

Thank you for this constructive comment. We agree that the Introduction should emphasize the scientific rationale and objectives rather than recount the history of manuscript development. We have therefore substantially shortened this passage and removed the detailed narrative concerning subsequent publications and the omission of analyses during peer review. To maintain transparency, we have retained a single sentence acknowledging that the study builds on previously generated sequencing resources and plastome analyses presented in our earlier bioRxiv preprint, while briefly identifying its relationship to our peer-reviewed studies.

The revised paragraph now focuses on investigating plastome conservation and localized variation in Aurantioideae. The objectives follow the analytical sequence of the manuscript: characterization of plastome organization and gene content, examination of LSC–IR boundary variation and recurrent and lineage-specific putative pseudogenization, assessment of maternal-lineage relationships within *Citrus*, and divergence-time estimation. The paragraph concludes with a brief summary of the principal findings and their contribution to understanding plastome evolution within a phylogenetic and temporal framework. These revisions preserve transparency about the relationship to our previous work while emphasizing the scientific purpose and contribution of the present study rather than its publication history.

**Lines 83–95:** Here, we investigate plastome conservation and localized variation in Aurantioideae within a phylogenetic and temporal framework. This study builds on previously generated sequencing resources and plastome analyses presented in our earlier bioRxiv preprint [17], complementing our peer-reviewed studies of plastome–nuclear phylogenetic discordance [9] and a large plastome inversion [3]. We first characterize plastome organization and gene content across the subfamily, then examine structural variation at the LSC–IR boundaries and recurrent patterns of putative gene pseudogenization. We also investigate additional lineage-specific coding-sequence disruptions affecting cemA, ycf15, rps4, and ycf1. Subsequently, we use whole-plastome phylogenetic analysis to assess maternal-lineage relationships within Citrus and estimate divergence times among major Aurantioideae lineages, with particular attention to Asia–Africa disjunctions. Together, these analyses reveal broadly conserved plastome organization alongside localized structural and coding-sequence variation, while providing a phylogenetic and temporal context for understanding plastome diversification within the subfamily.

# Below is a detailed assessment of the novelty of the results presented in this study.

# (1.1) First of all, no new raw data were generated specifically for the analysis reported in this manuscript. Indeed, the authors used sequences generated by their group which were produced in the context of previous, already published studies, and data come from publicly available sources (SRA short-read data and GenBank plastome sequences).

Thank you for this comment. We agree that no new raw sequencing data were generated specifically for the present study. To improve transparency, we have revised the manuscript and Supplementary Tables to clearly document the origin of all datasets used and to distinguish sequences generated in our previous work from those obtained from public repositories.

A total of 103 plastome accessions were analyzed, comprising 88 Aurantioideae accessions and 15 outgroup taxa. Among the 88 Aurantioideae accessions, 28 plastomes were generated from sequencing data produced in our previous study. In addition, publicly available raw sequencing data for 15 accessions had been retrieved and de novo assembled as part of the dataset used in our earlier work. The remaining Aurantioideae accessions were obtained as complete plastome sequences from public databases. The outgroup dataset consisted of 14 Rutaceae taxa outside Aurantioideae and Ailanthus altissima, which was used as the external outgroup.

In Materials and Methods,

**Lines 99–106**: The present study investigated plastome evolution within the subfamily Aurantioideae of the family Rutaceae. A total of 103 plastome accessions were analyzed, comprising 88 Aurantioideae accessions and 15 outgroup taxa. The dataset included 29 accessions for which sequencing data had been generated in our previous studies [3,9], 15 accessions represented by publicly available raw sequencing datasets, and 59 accessions represented by publicly available complete plastome sequences. Thus, no new sequencing data were generated specifically for the present study. A complete list of the taxa, data sources, and corresponding sequence accession numbers is provided in Supplementary Table S1.

Although the present study does not introduce newly generated raw sequencing data, we believe that the systematic reanalysis and integration of existing sequencing resources remain scientifically valuable. The rapid accumulation of publicly available high-throughput sequencing data, particularly short-read datasets, provides substantial opportunities for comparative genomic and evolutionary analyses that extend beyond

the original purposes for which those data were generated. Reanalysis of such datasets can reveal additional structural, sequence-level, phylogenetic, and evolutionary information that may not have been examined in the original studies.

In the present work, we integrate previously generated and publicly available data across a broad taxonomic sampling to characterize plastome conservation, investigate structural variation at the LSC–IR boundaries, identify recurrent and lineage-specific patterns of putative pseudogenization, reconstruct whole-plastome phylogenetic relationships, and estimate divergence times within Aurantioideae. The contribution of the study therefore lies in the comparative synthesis and evolutionary interpretation of these datasets rather than in the generation of new raw sequence data.

# (1.2) Second, the phylogenetic analyses desscribed in this manuscript resolved eight major cpDNA clusters within Citrus. However, contrary to the authors’ claim, this result does not appear to be particularly novel. Indeed, a 2009 study based on PCR-RFLP and trnL-trnF sequence data from chloroplast DNA analyzed 50 Citrus accessions and also placed them into eight major clusters. The paper is: Jena, S. N., Kumar, S., & Nair, N. K. (2009). Molecular phylogeny in Indian Citrus L. (Rutaceae) inferred through PCR-RFLP and trnL-trnF sequence data of chloroplast DNA. Scientia Horticulturae, 119(4), 403-416.

# As stated in the Abstract of that paper: “PCR-RFLP data showed a genetic distance ranging from 0 to 0.79 among 50 accessions of Citrus, and a cluster analysis, based on Neighbor-Joining (NJ) method, placed all the accessions in eight major clusters”.

# Importantly, the authors of the present manuscript have neither compared their results with those of this study nor cited it in any way.

# Similarly, another relatively recent paper, published in Nature, analyzed 58 Citrus accessions and presented a chloroplast genome phylogeny that also resolved a similar number of lineages within Citrus:

# Wu, G. A., Terol, J., Ibanez, V., López-García, A., Pérez-Román, E., Borredá, C., ... & Talon, M. (2018). Genomics of the origin and evolution of Citrus. Nature, 554(7692), 311-316.

# Again, the authors do not even cite this paper, despite its apparent importance in the context of Citrus phylogeny.

# Overall, these omissions not only call into question the novelty of the phylogenetic result reported in the present manuscript, but also raise concerns about how the results have been discussed and contextualized within the existing literature.

Thank you for highlighting these relevant studies and the need to better contextualize our findings. We have revised the Discussion to cite Jena et al. (2009) and explicitly acknowledge that the recovery of eight major clusters is not itself novel. We now explain that our analysis characterizes these relationships using complete plastome sequences, extending sequence coverage beyond selected markers without claiming novelty or improved resolution solely on this basis. We have also expanded comparisons with our previous analysis of 93 accessions using *matK* and the 34-plastome analysis of Carbonell-Caballero et al. (2015), identifying shared relationships among major groups and corresponding maternal affinities of individual accessions. Wu et al. (2018) is now cited when discussing hybridization, admixture, and the distinction between plastome and nuclear ancestry.

The revised Discussion reads as follows:

**Lines 416–438:** Several relationships corroborate earlier plastome studies. Our previous matK analysis of 93 accessions identified three principal groups corresponding to citron, pummelo, and mandarin, with Poncirus and Fortunella outside these groups [10]. Subsequently, Carbonell-Caballero et al. (2015), analyzing 34 plastomes, associated citron with Australian citrus, pummelo with C. micrantha, and mandarins with Ichang papeda, while recognizing Poncirus and Fortunella as distinct lineages [11]. Our tree similarly places the Citron cluster sister to the Australian assemblage, recovers sister Pummelo and Papeda clusters, and associates Ichang Papeda/Yuzu with the broader mandarin-related assemblage, while retaining distinct Poncirus and Fortunella clusters. The eight-cluster scheme therefore describes finer subdivisions within several previously recognized broader relationships. At the accession level, the placement of C. sinensis, C. aurantium, C. × paradisi, and C. limon within the Pummelo cluster, the association of C. aurantifolia with C. micrantha, and the inclusion of C. limonia within the Mandarin cluster are consistent with the maternal affinities reported by Carbonell-Caballero et al. (2015) [11]. These relationships illustrate how cultivated accessions with different taxonomic identities can share closely related plastomes, reflecting their maternal ancestry.

Relative to our earlier matK analysis, which emphasized citron, pummelo, and mandarin groups [10], the present tree provides a more differentiated description of the sampled plastome lineages. However, Jena et al. (2009) also recovered eight major clusters using PCR-RFLP data, so the cluster count itself should not be regarded as novel [59]. Our contribution is to reconstruct and characterize these relationships using complete plastome sequences, extending sequence coverage beyond selected markers. Oueslati et al. (2016), by comparison, developed diagnostic SNP markers from eight plastome regions to identify established Aurantioideae clades [8]. Their marker-based identification and our phylogenetic reconstruction serve related purposes, but their clades span multiple taxonomic levels and are not directly equivalent to our eight clusters.

# (1.3) Regarding the divergence-time analysis, the authors indicated two comparable Asia-Africa splits within Aurantioideae, whereas the divergence between Australian and Asian Citrus lineages was more recent. Concerning the latter split, this is not a novel result, as it is entirely consistent with the 2018 Nature paper by Wu et al., which also provided divergence-time estimates for these splits and, as noted above, appears not to have been considered in the discussion of the present manuscript. Therefore, perhaps the only potentially novel aspect of the divergence-time analysis is the identification of the two comparable Asia-Africa splits (~5 Ma) within Aurantioideae. However, regarding the reliability of this result, see point (2).

Thank you for this important comment. We agree that the evolutionary differentiation of Australian and Asian *Citrus* has been investigated previously and that Wu et al. (2018) should have been explicitly discussed. We have revised the Discussion to acknowledge their findings and clarify the contribution of our analysis.

In response to the methodological concerns raised in point (2), we re-estimated divergence times using MCMCtree with four calibration constraints and updated the Materials and Methods, Results, and Discussion accordingly. Importantly, the revised estimates concern deeper lineage separations involving Australian *Citrus*, rather than the subsequent radiation within the Australian clade discussed by Wu et al. (2018). These events are not directly equivalent; therefore, we do not interpret our estimates as confirmation of their reported radiation age.

Nevertheless, re-examining evolutionary relationships using plastome data within a broader taxonomic framework provides a valuable complementary perspective. Our analysis places divergences involving Australian *Citrus* alongside two geographically comparable Asia–Africa separations, *Citropsis*–*Naringi* and *Afraegle*–*Aegle*, estimated at approximately 19.51 and 18.14 Ma, respectively. The younger divergences involving Australian *Citrus* thus contribute to a broader comparison of the timing of lineage differentiation within Aurantioideae.

We have revised the manuscript to distinguish previously established findings from the additional comparative context provided here. We also clarify that lineage divergence dates do not directly establish the timing of geographic separation or dispersal. These revisions acknowledge the value of reassessing previous findings without overstating either the novelty of our results or their agreement with earlier divergence-time estimates.

In Results,

**Lines 331–342:** Two biogeographically parallel lineage separations were identified within Aurantioideae (Fig 4). In the Thanaka group, African Citropsis and Asian Naringi diverged at an estimated 19.51 Ma (95% HPD: 11.85–28.40 Ma). In the Bael fruit group, African Afraegle and Asian Aegle diverged at 18.14 Ma (95% HPD: 9.43–28.08 Ma) (Fig 4). Their similar posterior mean estimates and broadly overlapping HPD intervals indicate comparable early Miocene divergence times.

Divergences involving Australian Citrus had younger posterior mean estimates, although their HPD intervals overlapped those of the two Asia–Africa separations (Fig 4). The lineage comprising Australian Citrus, Clymenia polyandra, and Citrus medica diverged from the remaining sampled true citrus fruit trees at 14.71 Ma (95% HPD: 9.47–20.69 Ma) (Fig 4). Within this lineage, C. medica separated from Clymenia polyandra and Australian Citrus at 11.81 Ma (95% HPD: 7.47–16.88 Ma), followed by the separation of Clymenia polyandra from Australian Citrus at 10.57 Ma (95% HPD: 6.58–15.22 Ma; Fig 4).

In Discussion,

**Lines 446–469:** The divergence-time analysis extends this phylogenetic framework by placing major lineage separations within a temporal context. The estimated separations between *Citropsis* and *Naringi* and between *Afraegle* and *Aegle*, approximately 19.5 and 18.1 Ma, respectively, fall within the early Miocene. These broadly comparable estimates suggest a temporal parallel between two lineages exhibiting present-day Asia–Africa disjunctions, although similar point estimates do not establish synchronous geographic isolation or a shared cause. Progressive Himalayan–Tibetan uplift and associated changes in atmospheric circulation, monsoon dynamics, and habitat distribution provide a possible environmental context for changes in the ranges of their Asian ancestors [60]. However, these prolonged geological processes cannot be directly linked to the inferred separations, and dispersal, range fragmentation, and regional extinction remain alternative explanations for the observed distributions.

The younger divergences involving Australian *Citrus* may instead be considered in the context of changing dispersal opportunities between Southeast Asia and Australasia. Increased Miocene floristic exchange between the Sunda and Sahul regions provides a plausible regional framework but does not establish the route or timing of *Citrus* migration into Australia [61]. Wu et al. (2018) previously inferred a late Miocene radiation of Asian *Citrus*, followed by an early Pliocene radiation of Australian limes [5]. The present study offers a complementary plastome-based assessment within a broader Aurantioideae framework. However, the deeper separations examined here are not equivalent to diversification within the Australian clade and therefore do not directly confirm the previously reported radiation age. These findings highlight temporal variation among geographically differentiated lineages within Aurantioideae. Their biogeographic interpretation remains conditional on age uncertainty and the distinction between plastome divergence and geographic separation. Ancestral-range reconstruction and independent nuclear evidence will be needed to evaluate the processes underlying these distributions.

# (1.4) Results on structural variation and pseudogenization: these do indeed appear to be the only truly novel contribution of this study, but in my view, this is too limited to support a standalone paper. However, the authors themselves acknowledge: “Another represented a further development of the preprint’s structural observations by examining in detail an approximately 22 kb inversion in the *psbM*–*trnG-UCC* region and its associated short inverted repeats.” It therefore appears that at least part of the results on structural variation contained in this dataset has already been described in another paper by the authors.

Thank you for this important comment. We agree that the approximately 22-kb inversion in the *psbM–trnG-UCC* region was already characterized in our previous study and is not considered a novel result of the present manuscript.

The main novelty of this study lies in extending the analysis from individual structural observations to a broader comparative assessment of plastome evolution across Aurantioideae. Specifically, we identify and compare lineage-specific and recurrent changes at the LSC–IR boundaries, including variation involving *rpl22* and *rps19*, together with associated gene fragmentation, local gene loss, and pseudogenization. We also characterize the phylogenetic distribution and sequence-level basis of multiple pseudogenization events across the subfamily, with representative mutations further supported by raw-read alignments where available.

Therefore, the contribution of the present study is not the previously reported inversion itself, but the broader comparative framework that reveals how multiple forms of plastome structural and sequence-level variation are distributed across Aurantioideae.

# (2) Methodological weakness in the divergence-time analysis.

# The use of RelTime-ML in MEGA11 with a single calibration point is potentially problematic. RelTime is a relatively simple approach that may not adequately account for rate variation; moreover, although the use of a single calibration point may be appropriate, it is insufficient. A more robust approach, such as BEAST2 or MCMCTree with multiple fossil calibrations or secondary calibrations from the literature (if available), would strengthen the reliability and precision of the estimated divergence times.

Thank you for this valuable comment. We agree that relying on RelTime-ML with a single calibration point may limit the robustness and precision of divergence-time estimates. In response to this suggestion, we completely replaced the previous RelTime-ML analysis with a new divergence-time analysis using MCMCtree implemented in PAML v4.10.10.

The revised analysis incorporated four temporal constraints derived from published fossil evidence and secondary divergence estimates, including one root constraint and three internal minimum-age constraints. We also performed a prior-only analysis to evaluate the effective time priors before estimating the posterior divergence times. Posterior dating was conducted under an independent-rates relaxed-clock model using two independent MCMC chains, and convergence was assessed using rank-normalized (R-hat) and bulk and tail effective sample sizes.

In addition, we performed a calibration-sensitivity analysis by replacing the 47.8-Ma minimum-age constraint for the stem *Zanthoxylum* lineage with a 66-Ma minimum-age constraint based on the fossil *Rutaspermum biornatum*, while retaining all other analytical settings. This additional analysis allowed us to evaluate the sensitivity of the divergence-time estimates to an alternative fossil calibration and supported the robustness of the overall temporal patterns recovered.

Based on the new MCMCtree analysis, all divergence-time estimates were recalculated, and Fig 4 and the corresponding Materials and Methods, Results, and Discussion sections were revised accordingly.

In Materials and Methods,

**Lines 192–213: Divergence-time estimation**

Divergence times were estimated using MCMCtree in PAML v4.10.10 [36] with the approximate-likelihood method [37]. The ML topology inferred from the plastome alignment using IQ-TREE v2.3.6 [38] was fixed for molecular dating and rooted with Ailanthus altissima as the outgroup. The analysis included 47 taxa and a 77,661-bp nucleotide alignment. Branch lengths, gradient vectors, and the Hessian matrix required for the approximate-likelihood calculation were estimated using BASEML under the HKY85+Γ model with five discrete gamma rate categories and subsequently used in MCMCtree [37,39,40].

Four temporal constraints were applied following published fossil assessments and divergence estimates. The root separating *A. altissima* from the sampled Rutaceae was assigned a secondary soft bound of 100–117 Ma [B (1.00, 1.17)] [41]. Fossil-based soft minimum constraints were assigned to stem *Zanthoxylum* at 47.8 Ma [L (0.478)] [41], the stem *Clausena* + *Glycosmis* lineage at 27.23 Ma [L (0.2723)] [42], and stem *Citrus* at 11.6 Ma [L (0.116)] [43]. A prior-only analysis (usedata = 0) was first performed to evaluate the effective time priors. Posterior dating was then conducted using the approximate likelihood (usedata = 2) under an independent-rates relaxed clock (clock = 2) with a birth–death-sampling prior (BDparas = 1 1 0.1 m), rgene_gamma = 2 20 1, and sigma2_gamma = 1 10 1. Two independent MCMC chains were run, each with 200,000 burn-in iterations followed by 20,000 retained samples collected every 100 iterations. Convergence was assessed using rank-normalized R-hat and bulk and tail effective sample sizes [44]. Calibration sensitivity was assessed by replacing the 47.8-Ma minimum-age constraint for Zanthoxylum with a 66-Ma minimum-age constraint based on the fossil Rutaspermum biornatum [41], while keeping all other analytical settings unchanged.

In Results,

**Lines 331–342:** Two biogeographically parallel lineage separations were identified within Aurantioideae (Fig 4). In the Thanaka group, African Citropsis and Asian Naringi diverged at an estimated 19.51 Ma (95% HPD: 11.85–28.40 Ma). In the Bael fruit group, African Afraegle and Asian Aegle diverged at 18.14 Ma (95% HPD: 9.43–28.08 Ma) (Fig 4). Their similar posterior mean estimates and broadly overlapping HPD intervals indicate comparable early Miocene divergence times.

Divergences involving Australian Citrus had younger posterior mean estimates, although their HPD intervals overlapped those of the two Asia–Africa separations (Fig 4). The lineage comprising Australian Citrus, Clymenia polyandra, and Citrus medica diverged from the remaining sampled true citrus fruit trees at 14.71 Ma (95% HPD: 9.47–20.69 Ma) (Fig 4). Within this lineage, C. medica separated from Clymenia polyandra and Australian Citrus at 11.81 Ma (95% HPD: 7.47–16.88 Ma), followed by the separation of Clymenia polyandra from Australian Citrus at 10.57 Ma (95% HPD: 6.58–15.22 Ma; Fig 4).

In Discussion,

**Lines 446–469:** The divergence-time analysis extends this phylogenetic framework by placing major lineage separations within a temporal context. The estimated separations between *Citropsis* and *Naringi* and between *Afraegle* and *Aegle*, approximately 19.5 and 18.1 Ma, respectively, fall within the early Miocene. These broadly comparable estimates suggest a temporal parallel between two lineages exhibiting present-day Asia–Africa disjunctions, although similar point estimates do not establish synchronous geographic isolation or a shared cause. Progressive Himalayan–Tibetan uplift and associated changes in atmospheric circulation, monsoon dynamics, and habitat distribution provide a possible environmental context for changes in the ranges of their Asian ancestors [60]. However, these prolonged geological processes cannot be directly linked to the inferred separations, and dispersal, range fragmentation, and regional extinction remain alternative explanations for the observed distributions.

The younger divergences involving Australian *Citrus* may instead be considered in the context of changing dispersal opportunities between Southeast Asia and Australasia. Increased Miocene floristic exchange between the Sunda and Sahul regions provides a plausible regional framework but does not establish the route or timing of *Citrus* migration into Australia [61]. Wu et al. (2018) previously inferred a late Miocene radiation of Asian *Citrus*, followed by an early Pliocene radiation of Australian limes [5]. The present study offers a complementary plastome-based assessment within a broader Aurantioideae framework. However, the deeper separations examined here are not equivalent to diversification within the Australian clade and therefore do not directly confirm the previously reported radiation age. These findings highlight temporal variation among geographically differentiated lineages within Aurantioideae. Their biogeographic interpretation remains conditional on age uncertainty and the distinction between plastome divergence and geographic separation. Ancestral-range reconstruction and independent nuclear evidence will be needed to evaluate the processes underlying these distributions.

# (3) Insufficient description of sample and sequencing data.

# Lines 94-96: this sentence don’t provide the reader with any quantitative information about the relative contributions of each data source. I suggest to specify the numbers of each subset of samples in the main text. Moreover, ensure that tables in supplementary material provides comprehensive details for each one of them, and also consider adding a brief summary table in the main text (in the main text, no tables are currently reported).

Thank you for this helpful comment. We agree that the original description did not provide sufficient quantitative information regarding the contribution of each data source. We have therefore revised the Materials and Methods section to specify the number of accessions included from each data source and have also revised the Supplementary Tables to provide comprehensive sample-level information.

A total of 103 plastome accessions were analyzed, comprising 88 Aurantioideae accessions and 15 outgroup taxa. Among the 88 Aurantioideae accessions, 28 were represented by sequencing data generated in our previous studies, 15 were represented by publicly available raw sequencing datasets that had been de novo assembled as part of the dataset used in our earlier work, and the remaining 45 Aurantioideae accessions were obtained as complete plastome sequences from public databases. The outgroup dataset consisted of 14 Rutaceae taxa outside Aurantioideae together with *Ailanthus altissima*, which was used as the external outgroup. Thus, no new sequencing data were generated specifically for the present study.

We considered your suggestion to include a summary table in the main text. However, because the complete sample-level information, including taxon names, data sources, accession numbers, and dataset categories, is already provided in the Supplementary Tables, we decided not to add an additional table to the main manuscript in order to avoid duplication. Instead, we added a concise quantitative summary in the Materials and Methods section so that readers can immediately understand the composition of the dataset, while the detailed information remains available in the Supplementary Tables.

The Materials and Methods section has been revised as follows:

**Lines 99–106**: The present study investigated plastome evolution within the subfamily Aurantioideae of the family Rutaceae. A total of 103 plastome accessions were analyzed, comprising 88 Aurantioideae accessions and 15 outgroup taxa. The dataset included 29 accessions for which sequencing data had been generated in our previous studies [3,9], 15 accessions represented by publicly available raw sequencing datasets, and 59 accessions represented by publicly available complete plastome sequences. Thus, no new sequencing data were generated specifically for the present study. A complete list of the taxa, data sources, and corresponding sequence accession numbers is provided in Supplementary Table S1.

# In addition, I also note that the Materials and Methods section does not provide essential information about the sequencing data used for plastome assembly. The current text states that DNA extraction and short-read sequencing were performed as described in previous studies, but this is insufficient for a standalone research article. I recommend that the authors include the following details in the present manuscript: sequencing platform and chemistry; average sequencing depth (coverage), total number of raw reads generated per sample (or the range); some quality control statistics. This holds for both newly generated data produced by the authors, and also SRA data that were downloaded and re-assembled by the authors (where available).

Thank you for highlighting the need for a more complete description of the sequencing data used for plastome assembly. In response, we have revised the Materials and Methods section to provide the relevant DNA extraction, library preparation, sequencing platform, and read-processing information in the present manuscript rather than relying solely on our previous study.

We have also added Supplementary Table S2 to provide per-sample sequencing and mapping statistics for the 44 filtered-read datasets used for *de novo* plastome assembly, including both sequencing data generated in our previous study and publicly available raw-read datasets retrieved from the SRA. For the quality-filtered reads, we report the number of read pairs, total filtered bases, average read length, Q20 and Q30 percentages, and GC content; these statistics were calculated using a custom Python script. In addition, Qualimap v2.1.1 was used to obtain mapping statistics after the filtered reads were mapped back to the corresponding assembled plastomes, including the percentage of mapped reads, mean sequencing coverage, and mean mapping quality. These additions provide quantitative information on sequencing depth and read quality for each dataset used for plastome assembly.

The Materials and Methods section has been revised accordingly to describe these procedures and the associated sequencing information.

**Lines 113–117:** DNA quality was evaluated by electrophoresis on a 1% agarose gel, and DNA concentration was determined using a DNA BR Assay Kit (Thermo Fisher Scientific, USA). Sequencing libraries were prepared from total genomic DNA by Novogene (Singapore) using the NEBNext Ultra DNA Library Prep Kit for Illumina (New England Biolabs, USA) and sequenced on an Illumina NovaSeq 6000 platform to generate 150-bp paired-end reads.

**Lines 120–123:** Summary statistics for the filtered reads, including the number of read pairs, total filtered bases, average read length, Q20 and Q30 percentages, and GC content, were calculated using a custom Python script (Supplementary Table S2).

**Lines 130–132:** Qualimap v2.1.1 [22] was used to obtain quantitative mapping statistics, including the percentage of reads mapped to the assembled plastome, mean sequencing coverage, and mean mapping quality (Supplementary Table S2).

# (4) Lack of background on pseudogenization in the Introduction.

# The introduction does not include any substantive background on plastid gene pseudogenization, despite this being one of the main novel contributions of the study. Currently, the term “pseudogenization” appears only in the abstract and results sections. I recommend adding a dedicated paragraph that would: define pseudogenes and their causes (in the context of plastid genomes); summarize the current understanding of pseudogenization in plant plastomes, noting that it is relatively common and often associated with functional gene transfer to the nucleus or relaxed selective constraints; mention that pseudogenization events are frequently correlated with structural rearrangements; highlight why this is particularly relevant in Aurantioideae, considering the group’s complex evolutionary history, etc.

Thank you for highlighting this omission. We have added a dedicated paragraph to the Introduction defining plastid pseudogenes and describing the sequence disruptions commonly used to identify putative pseudogenization, including frameshifts, premature termination codons, loss of recognizable start codons, and substantial deletions. The paragraph summarizes relevant findings from previous studies, discusses relaxed selective constraints and functional replacement by nuclear-encoded copies as possible evolutionary contexts, and explains how pseudogenization can accompany structural changes, including IR

boundary shifts. We also clarify the relevance of examining these patterns within the structural and phylogenetic framework of Aurantioideae. To accurately reflect the scope of our study, we explicitly distinguish sequence-based inference of putative pseudogenization from experimental demonstration of gene function or identification of the underlying evolutionary mechanism. Supporting references have been added.

**Lines 67–82:** Gene pseudogenization represents another aspect of plastome evolution that can occur within an otherwise conserved genomic framework [13]. Pseudogenes are gene-derived sequences that have lost their original coding capacity; in comparative plastome analyses, putative pseudogenes are generally identified by disruptions such as frameshift mutations, premature termination codons, loss of recognizable start codons, or substantial deletions [14]. Such changes have been documented across diverse plant lineages and may accumulate under relaxed selective constraints or, in some cases, following functional replacement by a nuclear-encoded copy [14,15]. Pseudogenization can also accompany structural changes, including shifts in IR boundaries that generate truncated gene copies, linking variation in gene integrity with plastome architecture. However, sequence disruption alone does not establish the underlying evolutionary mechanism or demonstrate loss of biological function, particularly for putative coding regions whose functional status remains uncertain [16]. In Aurantioideae, examining these disruptions alongside boundary variation and plastome phylogeny provides an opportunity to distinguish shared patterns from lineage-specific changes within a taxonomically complex group. Accordingly, comparative assessment of gene integrity can complement structural and phylogenetic analyses without implying experimental validation of gene function.

# (5) Frequent lack of appropriate literature support for the reported statements, and high rate of self citation.

Thank you for identifying this concern. We have carefully reviewed the entire manuscript and added appropriate independent references to statements that previously lacked sufficient bibliographic support. We also expanded the reference list from 37 to 61 references.

# There is recurrent lack of bibliographic citations supporting the statements made by the authors.

# Some examples include:

# Lines 42-48: some literature citations are needed here to support these statements (although some of them are quite well known).

# Lines 48-49: citation needed.

# Lines 62-64: citation needed.

# Line 127: please add the correct citation which, according to TimeTree website, should be:

# S. Kumar, M. Suleski, J.E. Craig, A.E. Kasprowicz, M. Sanderford, M. Li, G. Stecher, and S.B. Hedges, 2022. TimeTree 5: An Expanded Resource for Species Divergence Times. Molecular Biology and Evolution, DOI: 10.1093/molbev/msac174.

# Moreover, the TimeTree version should be specified. Line 139: citation needed for the tool “genbank_to”; see: <https://genbank-to.readthedocs.io/en/latest/changelog.html#citation>. Lines 266-269: Some literature citations are needed.

# Lines 279-281: citation needed.

We have thoroughly revised the manuscript to provide appropriate references for scientific statements, methodological descriptions, and interpretations. Relevant independent literature, including the references recommended by you, has been added throughout the revised manuscript.

Thank you for providing the correct TimeTree citation. However, the original TimeTree-based calibration and the associated text have been removed because we completely repeated the divergence-time analysis using the approximate-likelihood method implemented in MCMCtree within PAML v4.10.10. The revised analysis uses multiple temporal constraints based on published fossil assessments and divergence estimates rather than the previous TimeTree-based approach. Appropriate references for MCMCtree, PAML, and the temporal constraints have been added to the revised Materials and Methods.

# In addition, as many as 7 of the 37 references (i.e., references nos. 3, 4, 5, 7, 31, 32, and 34) include one or more of the co-authors of the present study among their authors, corresponding to a self-citation rate of 18.9%. Although many of these citations may be considered appropriate, this figure is rather striking; nevertheless, it could be also partly due to the low overall number of references (see above comment).

We acknowledge your concern and have carefully reassessed all self-citations. We substantially expanded the coverage of independent literature. The revised manuscript now contains 61 references, of which eight are publications authored or coauthored by the present authors. Thus, the proportion of self-citations has decreased from 18.9% (7/37) to 13.1% (8/61), and the revised reference list is predominantly based on independent literature.

# Minor comments

# (1) Lines 35-37: True, but authors should not forget introgression and cp capture in several plant groups, acting as a confounding factor. Indeed, the authors discuss these limitations at lines 51-53. I only suggest to rephrase a little bit this sentence in order to avoid apparat contradictory statements.

Thank you for this helpful suggestion. We agree that the original wording could be interpreted as implying that plastome-based phylogenetic relationships necessarily reflect the complete evolutionary history of the taxa. We have therefore slightly revised the sentence to clarify that plastomes are particularly informative for tracing plastome lineages, while the potential effects of introgression and plastome capture are discussed later in the Introduction.

The revised sentence reads as follows:

**Lines 35–37:** Plastomes have become widely used resources in plant systematics because they can be assembled efficiently from genome-skimming data and provide abundant phylogenetic information for tracing plastome lineages [1,2].

# (2) Line 86: Instead of “in Aurantioideae of Rutaceae”, perhaps “in the subfamily Aurantioideae of the Rutaceae” could be more appropriate and clearer.

Thank you for this suggestion. We have revised the sentence for clarity as follows:

**Lines 99–100:** The present study investigated plastome evolution within the subfamily Aurantioideae of the family Rutaceae.

# (3) Lines 134-136: Did all the reported structural features were identified by the authors visually? Or by using which tools? These methodological details should be better specified; please also note that dedicated tools such as IRscope could be used for precise IR boundary quantification.

Thank you for this comment. We initially attempted to use IRscope; however, compatibility issues arose with our manually curated GenBank files. Therefore, the LSC–IR and IR–SSC boundaries were examined and confirmed manually based on the annotated plastome sequences. We have revised the Materials and Methods section to clarify that the boundary analysis was performed manually.

**Lines 155–157:** To further investigate structural variation at plastome junctions, boundary regions were manually examined and compared among representative Aurantioideae plastomes, with particular attention to the junctions between the LSC and the IRs.

# (4) Lines 142-142: In pseudogene identification, do the authors validated each putative pseudogenization event by inspecting the raw read alignment in IGV, for confirming that the mutation was supported by a sufficient coverage of reads?

Thank you for this valuable comment. Where raw sequencing reads were available, we validated the candidate pseudogenization events by inspecting the corresponding read alignments in IGV and confirming that the mutations were supported by sufficient read coverage. Representative IGV screenshots are now provided in Supplementary Figs S2, S3, and S5. For example, the nucleotide substitution generating the premature stop codon in *rpl22* showed 100% support for the altered base among mapped reads, indicating that the mutation was unlikely to result from a sequencing or assembly error.

We have revised the Materials and Methods and Results sections accordingly.

In Materials and methods,

**Lines 180–184:** Where raw sequencing reads were available, candidate pseudogenization events were further validated by visual inspection of read alignments in IGV. Read coverage across the affected regions was examined to confirm that the sequence variants responsible for pseudogenization were supported by the underlying raw reads rather than resulting from assembly or annotation errors.

In Results,

**Lines 269–271:** Representative IGV read alignments showed that the mutations associated with putative pseudogenization of *rpl22*, *orf56* and *ycf68* were consistently supported by mapped sequencing reads (S2 Fig for *rpl22* and *orf56*; S3 Fig for *ycf68*).

**Lines 276–280:** In *Afraegle paniculata*, *cemA* was inferred to be pseudogenized relative to the intact sequence in *Aegle marmelos*, with a 5-bp insertion of adenine residues disrupting the coding sequence (S4 and S5 Figs). In *Aegle marmelos*, *ycf15* lacked a recognizable start codon compared with the intact sequence in *Citrus sinensis* (S4 and S5 Figs).

# (5) Lines 173-174: This is Discussion.

Thank you for this comment. In response to this and a similar comment from Reviewer 1, we revised the Results section to remove discussion-like interpretations. The sentence previously appearing at Lines 173–174 has therefore been removed.

# (6) Lines 189: the reference to a figure is needed here.

Thank you for this comment. Following the revised divergence-time analysis, we updated the corresponding Results section and have now cited the revised Fig 4 at the appropriate location.

**Lines 331–342:** Two biogeographically parallel lineage separations were identified within Aurantioideae (Fig 4). In the Thanaka group, African Citropsis and Asian Naringi diverged at an estimated 19.51 Ma (95% HPD: 11.85–28.40 Ma). In the Bael fruit group, African Afraegle and Asian Aegle diverged at 18.14 Ma (95% HPD: 9.43–28.08 Ma) (Fig 4). Their similar posterior mean estimates and broadly overlapping HPD intervals indicate comparable early Miocene divergence times.

Divergences involving Australian Citrus had younger posterior mean estimates, although their HPD intervals overlapped those of the two Asia–Africa separations (Fig 4). The lineage comprising Australian Citrus, Clymenia polyandra, and Citrus medica diverged from the remaining sampled true citrus fruit trees at 14.71 Ma (95% HPD: 9.47–20.69 Ma) (Fig 4). Within this lineage, C. medica separated from Clymenia polyandra and Australian Citrus at 11.81 Ma (95% HPD: 7.47–16.88 Ma), followed by the separation of Clymenia polyandra from Australian Citrus at 10.57 Ma (95% HPD: 6.58–15.22 Ma; Fig 4).

# (7) Line 191: “more recent”: please quantify.

Thank you for this comment. We have revised the text to quantify the comparison more explicitly by reporting the posterior mean divergence times and 95% HPD intervals for the Australian Citrus lineage and comparing them with the older Asia–Africa separations.

**Lines 336–342:** Divergences involving Australian Citrus had younger posterior mean estimates, although their HPD intervals overlapped those of the two Asia–Africa separations (Fig 4). The lineage comprising Australian Citrus, Clymenia polyandra, and Citrus medica diverged from the remaining sampled true citrus fruit trees at 14.71 Ma (95% HPD: 9.47–20.69 Ma) (Fig 4). Within this lineage, C. medica separated from Clymenia polyandra and Australian Citrus at 11.81 Ma (95% HPD: 7.47–16.88 Ma), followed by the separation of Clymenia polyandra from Australian Citrus at 10.57 Ma (95% HPD: 6.58–15.22 Ma; Fig 4).

# (8) Line 220: “in contrast”: it is not clear to me where the two statements are opposed.

Thank you for this comment. Following the revisions made to the Results section in response to Reviewer 1, the previous wording containing “in contrast” has been removed. The revised text now presents the pseudogenization patterns directly and more clearly, as follows:

**Lines 263–269:** The *infA* gene was inferred to be pseudogenized in all examined taxa, whereas putative pseudogenization of *ycf68* and *orf56* occurred in multiple lineages across the phylogeny (Fig 2). Sequence comparisons showed that *orf56* pseudogenization was associated with the insertion of a cytosine nucleotide, resulting in a frameshift mutation (Fig 2). Similarly, *ycf68* pseudogenization was associated with either the deletion of thymine residues within a poly-T region or the insertion of adenine residues, both of which resulted in frameshift mutations (Fig 2).

# (9) Lines 245-247: see my previous comment to lines 65-74.

Thank you for this comment. We have revised the opening of the Discussion to focus on the scientific findings and significance of the study, while retaining only a brief statement acknowledging the earlier preprint.

**Lines 345–352:** The present study provides an integrated assessment of plastome evolution and diversification within Aurantioideae through comparative plastome analyses, evaluation of pseudogenization patterns, phylogenetic inference, and divergence-time estimation. Aurantioideae plastomes were highly conserved in overall organization and gene content, with localized variation concentrated at LSC–IR boundaries and in genes exhibiting putative pseudogenization. This combination of broad conservation and localized change provides a framework for examining structural and coding-sequence evolution alongside phylogenetic relationships. Some of these plastome-based analyses were initially reported in our earlier preprint [17] but had not previously been developed in peer-reviewed form.

# (10) Lines 260-262: This paragraph on *Clymenia polyandra* presents several issues. The phrase "has not formally undergone a name change" is unscientific in tone, while "it is expected to be included" is speculative. I suggest reformulating more cautiously and, in an evidence, -based manner, for example: "In our plastome phylogeny, Clymenia polyandra was placed within the Citrus clade, suggesting that this taxon may be a member of Citrus...". And what about current phylogenetic information from literature about this genus?

# See e.g.:

# Bayer, R. J., Mabberley, D. J., Morton, C., Miller, C. H., Sharma, I. K., Pfeil, B. E., ... & Sykes, S. (2009). A molecular phylogeny of the orange subfamily (Rutaceae: Aurantioideae) using nine cpDNA sequences. American Journal of Botany, 96(3), 668-685.

# Oueslati, A., Ollitrault, F., Baraket, G., Salhi-Hannachi, A., Navarro, L., & Ollitrault, P. (2016). Towards a molecular taxonomic key of the Aurantioideae subfamily using chloroplastic SNP diagnostic markers of the main clades genotyped by competitive allele-specific PCR. BMC genetics, 17(1), 118.

# The results described by the authors should be presented in light of prior knowledge available in the literature. Furthermore, the statement "From this perspective, the present analysis effectively represents a 56-accession study" should also be rephrased in a clearer way.

Thank you for recommending a more precise, evidence-based treatment. We have removed the statements “has not formally undergone a name change” and “it is expected to be included.” The revised Discussion reports that *Clymenia polyandra* was sister to the sampled Australian species and places this result in the context of Bayer et al. (2009), whose phylogeny supported an expanded circumscription of *Citrus*. We also clarify that, although *Clymenia polyandra* is retained as the name used in this manuscript, POWO treats it as a synonym of *Citrus polyandra*. Its inclusion within *Citrus* is therefore presented as consistent with prior knowledge. Oueslati et al. (2016) is also discussed in relation to the identification of established Aurantioideae clades using diagnostic plastome SNPs. Finally, we have replaced the ambiguous statement about “effectively” representing 56 accessions with a direct description of the whole-plastome analysis of 56 accessions.

**Lines 407–438:** Alongside the localized structural and coding-sequence changes identified here, the whole-plastome analysis of 56 accessions recovered eight major clusters within the sampled Citrus assemblage: Pummelo, Papeda, Ichang Papeda/Yuzu, Mandarin, Poncirus, Fortunella, Australian, and Citron. Clymenia polyandra was sister to the sampled Australian species, with this assemblage sister to the Citron cluster. Its position within the true citrus group agrees with the expanded circumscription of Citrus supported by Bayer et al. (2009) [12]. Although the name Clymenia polyandra is retained here following the NCBI taxonomy database, Plants of the World Online (POWO) recognizes it as a synonym of Citrus polyandra; its inclusion within Citrus therefore accords with established phylogenetic and taxonomic treatments.

Several relationships corroborate earlier plastome studies. Our previous matK analysis of 93 accessions identified three principal groups corresponding to citron, pummelo, and mandarin, with Poncirus and Fortunella outside these groups [10]. Subsequently, Carbonell-Caballero et al. (2015), analyzing 34 plastomes, associated citron with Australian citrus, pummelo with C. micrantha, and mandarins with Ichang papeda, while recognizing Poncirus and Fortunella as distinct lineages [11]. Our tree similarly places the Citron cluster sister to the Australian assemblage, recovers sister Pummelo and Papeda clusters, and associates Ichang Papeda/Yuzu with the broader mandarin-related assemblage, while retaining distinct Poncirus and Fortunella clusters. The eight-cluster scheme therefore describes finer subdivisions within several previously recognized broader relationships. At the accession level, the placement of C. sinensis, C. aurantium, C. × paradisi, and C. limon within the Pummelo cluster, the association of C. aurantifolia with C. micrantha, and the inclusion of C. limonia within the Mandarin cluster are consistent with the maternal affinities reported by Carbonell-Caballero et al. (2015) [11]. These relationships illustrate how cultivated accessions with different taxonomic identities can share closely related plastomes, reflecting their maternal ancestry.

Relative to our earlier matK analysis, which emphasized citron, pummelo, and mandarin groups [10], the present tree provides a more differentiated description of the sampled plastome lineages. However, Jena et al. (2009) also recovered eight major clusters using PCR-RFLP data, so the cluster count itself should not be regarded as novel [59]. Our contribution is to reconstruct and characterize these relationships using complete plastome sequences, extending sequence coverage beyond selected markers. Oueslati et al. (2016), by comparison, developed diagnostic SNP markers from eight plastome regions to identify established Aurantioideae clades [8]. Their marker-based identification and our phylogenetic reconstruction serve related purposes, but their clades span multiple taxonomic levels and are not directly equivalent to our eight clusters.

# (11) Line 276: The Isthmus of Panama is geographically distant from the Afro-Asian region, and its connection to plant dispersal between Asia and Africa is highly speculative. I recommend removing the reference to the Isthmus of Panama and focusing instead on climatic or geological changes that more directly affected the Afro-Asian region, or, as an alternative, to better detail how the closure of the Isthmus of Panama could have affected plant dispersal between Asia and Africa, with appropriate literature citations.

Thank you for this important comment. We agree that the previous reference to the Isthmus of Panama was geographically indirect and overly speculative in the context of Asia–Africa plant disjunctions. We have therefore removed this reference and revised the Discussion to focus on geological and climatic processes more relevant to the Afro-Asian region. In particular, we now discuss Himalayan–Tibetan uplift and associated changes in atmospheric circulation, monsoon dynamics, and habitat distribution as a possible environmental context, while clearly acknowledging that these processes cannot be directly linked to the inferred divergence events and that dispersal, range fragmentation, and regional extinction remain alternative explanations.

**Lines 446–456:** The divergence-time analysis extends this phylogenetic framework by placing major lineage separations within a temporal context. The estimated separations between *Citropsis* and *Naringi* and between *Afraegle* and *Aegle*, approximately 19.5 and 18.1 Ma, respectively, fall within the early Miocene. These broadly comparable estimates suggest a temporal parallel between two lineages exhibiting present-day Asia–Africa disjunctions, although similar point estimates do not establish synchronous geographic isolation or a shared cause. Progressive Himalayan–Tibetan uplift and associated changes in atmospheric circulation, monsoon dynamics, and habitat distribution provide a possible environmental context for changes in the ranges of their Asian ancestors [60]. However, these prolonged geological processes cannot be directly linked to the inferred separations, and dispersal, range fragmentation, and regional extinction remain alternative explanations for the observed distributions.
