# Supplementary material for: Plastome evolution in Aurantioideae and plastome-based classification of Citrus
Source: PLoS One. 2026 Oct 7;21(10):e0349882. doi: 10.1371/journal.pone.0349882 (PMC13645046; doi:10.1371/journal.pone.0349882)
Supplement: Peer review history

# Peer review: Decision Letter 0

**DOI:** 10.1371/journal.pone.0349882.r001

**Authors:** Branislav T. Šiler

24 Aug 2026

Dear Dr. Nagano,

Thank you for submitting your manuscript to PLOS One. After careful consideration, we feel that it has merit but does not fully meet PLOS One’s publication criteria as it currently stands. Therefore, we invite you to submit a revised version of the manuscript that addresses the points raised during the review process.

==============================

**Both reviewers noted serious flaws in the methodology used and the results' quality derived from it. Some major points are:**

**- no new raw data were generated specifically for the analysis reported in this manuscript (Reviewer #2), but already published sequences and publicly available data were only used.**

**- for the phylogenetic analyses, the authors failed to cite and compare their findings with major literature findings.**

**- results on structural variation and pseudogenization - it appears that at least part of them has already been described in another paper by the authors (Reviewer #2).**

**- the common genome characterisation was not reported (Reviewer #1).**

**- divergence-time analysis - RelTime-ML in MEGA11 with a single calibration point is considered insufficient (Reviewer #2).**

**- essential information about the sequencing data used for plastome assembly was not provided (Reviewer #2).**

**- frequent lack of appropriate literature support for the reported statements, and high rate of self-citation (Reviewer #2).**

**Regarding fundamental methodology problems this study suffers from, I advise the authors to meticulously study the reviewers' comments, try to perform additional analyses, and considerably transform the manuscript, thus raising its overall quality. Please provide point-by-point reply to reviewers' comments.**

==============================

Please submit your revised manuscript by Oct 23 2026 11:59PM. If you will need more time than this to complete your revisions, please reply to this message or contact the journal office at plosone@plos.org. When you're ready to submit your revision, log on to https://www.editorialmanager.com/pone/ and select the 'Submissions Needing Revision' folder to locate your manuscript file.

- A letter that responds to each point raised by the academic editor and reviewer(s). You should upload this letter as a separate file labeled 'Response to Reviewers'.
- A marked-up copy of your manuscript that highlights changes made to the original version. You should upload this as a separate file labeled 'Revised Manuscript with Track Changes'.
- An unmarked version of your revised paper without tracked changes. You should upload this as a separate file labeled 'Manuscript'.

If you would like to make changes to your financial disclosure, please include your updated statement in your cover letter. Guidelines for resubmitting your figure files are available below the reviewer comments at the end of this letter.

If applicable, we recommend that you deposit your laboratory protocols in protocols.io to enhance the reproducibility of your results. Protocols.io assigns your protocol its own identifier (DOI) so that it can be cited independently in the future. For instructions see: https://journals.plos.org/plosone/s/submission-guidelines#loc-laboratory-protocols. Additionally, PLOS One offers an option for publishing peer-reviewed Lab Protocol articles, which describe protocols hosted on protocols.io. Read more information on sharing protocols at https://plos.org/protocols?utm_medium=editorial-email&utm_source=authorletters&utm_campaign=protocols.

As the corresponding author, your ORCID iD is verified in the submission system and will appear in the published article. PLOS supports the use of ORCID, and we encourage all coauthors to register for an ORCID iD and use it as well. Please encourage your coauthors to verify their ORCID iD within the submission system before final acceptance, as unverified ORCID iDs will not appear in the published article. *Only*  the individual author can complete the verification step; PLOS staff *cannot*  verify ORCID iDs on behalf of authors.

We look forward to receiving your revised manuscript.

Kind regards,

Branislav T. Šiler, Ph.D.

Academic Editor

PLOS One

Journal Requirements:

When submitting your revision, we need you to address these additional requirements.

1. Please ensure that your manuscript meets PLOS ONE's style requirements, including those for file naming. The PLOS ONE style templates can be found at

https://journals.plos.org/plosone/s/file?id=wjVg/PLOSOne_formatting_sample_main_body.pdf and

https://journals.plos.org/plosone/s/file?id=ba62/PLOSOne_formatting_sample_title_authors_affiliations.pdf

2. Please note that PLOS One has specific guidelines on code sharing for submissions in which author-generated code underpins the findings in the manuscript. In these cases, we expect all author-generated code to be made available without restrictions upon publication of the work. Please review our guidelines at https://journals.plos.org/plosone/s/materials-and-software-sharing#loc-sharing-code and ensure that your code is shared in a way that follows best practice and facilitates reproducibility and reuse.

3. Thank you for stating the following financial disclosure:

"This work was partially supported by a Grant-in-Aid for Scientific Research (18K05623) from the Japan Society for the Promotion of Science to Y.N. "

Please state what role the funders took in the study.  If the funders had no role, please state: "The funders had no role in study design, data collection and analysis, decision to publish, or preparation of the manuscript."

If this statement is not correct you must amend it as needed.

Please include this amended Role of Funder statement in your cover letter; we will change the online submission form on your behalf.

4.  Thank you for stating the following in the Competing Interests/Financial Disclosure * (delete as necessary) section:

"The authors declare that they have no competing interests related to this work. For transparency, Y.N. conducts collaborative research with Senoo Suisan, Inc. and Sante Laboratories, Inc., and serves as a director of Smart Review Technologies Co., Ltd. These activities are not related to the present study."

We note that you received funding from a commercial source: "Smart Review Technologies Co., Ltd"

Please provide an amended Competing Interests Statement that explicitly states this commercial funder, along with any other relevant declarations relating to employment, consultancy, patents, products in development, marketed products, etc.

Within this Competing Interests Statement, please confirm that this does not alter your adherence to all PLOS ONE policies on sharing data and materials by including the following statement: "This does not alter our adherence to PLOS ONE policies on sharing data and materials.” (as detailed online in our guide for authors http://journals.plos.org/plosone/s/competing-interests).  If there are restrictions on sharing of data and/or materials, please state these. Please note that we cannot proceed with consideration of your article until this information has been declared.

Please include your amended Competing Interests Statement within your cover letter. We will change the online submission form on your behalf.

5. If the reviewer comments include a recommendation to cite specific previously published works, please review and evaluate these publications to determine whether they are relevant and should be cited. There is no requirement to cite these works unless the editor has indicated otherwise.

[Note: HTML markup is below. Please do not edit.]

Reviewers' comments:

Reviewer's Responses to Questions

**Comments to the Author**

1. Is the manuscript technically sound, and do the data support the conclusions?

Reviewer #1: Yes

Reviewer #2: Partly

**********

2. Has the statistical analysis been performed appropriately and rigorously?

Reviewer #1: I Don't Know

Reviewer #2: No

**********

3. Have the authors made all data underlying the findings in their manuscript fully available?

The PLOS Data policy

Reviewer #1: Yes

Reviewer #2: Yes

**********

4. Is the manuscript presented in an intelligible fashion and written in standard English?

Reviewer #1: Yes

Reviewer #2: Yes

**********

Reviewer #1: The manuscript describes on the characterisation of the complete plastid genome sequences of members of Aurantioideae. My comments are as follows:

1) A mixture use of "chloroplast genome" and "plastid genome" was used in abstract and main text. Please be consistent. Based on the title, "plastid genome" and "plastome" are more preferable.

2) It could be just me, but the arrangement of the workflow is weird. The authors should be characterising the genome first, then analyse the genome, perform genome comparative analysis, then phylogenomics analysis and divergence time analysis. However, the authors started with phylogenonomic and divergence time, followed by genome comparative. The common genome characterisation was not reported. There is no information on the number of genes annotated, etc. It could be "useless" in the author's perspective, but the number of genes could also indicate some genome evolutionary pattern.

3) The reporting of pseudogenised genes is not practical, in my opinion. The outcome is bioinformatics-based. In a strict manner, a gene can be regarded as pseudo for good when it is proven of its functionality through wet lab. Wet lab was not performed here. The annotation of pseudo genes are just at predicted-level. Having a large amount of discussion on the predicted-to-be-pseudogenised genes seemed to be illogical.

4) The annotation of genes are based on the online software GeSeq. Based on my understanding, the latest version (which is not new), does not commonly annotates orfs, ycf15, ycf68 for most angiosperm. Therefore, it strucks my mind that an old/outdated annotated GenBank reference species was used for annotation, which could be inaccurate. Although gene annotations are predictive; GeSeq has been updated over time to provide an accurate annotation for plastid genome, in my opinion.

5) I have no idea how many new plastomes were reported in this study. I believe that most of the information can be presented in the Supplementary material, yet, it is still essential to be written out in the main text.

6) In most genome comparative analysis at a subfamily level, performing MAUVE could be more useful in providing information of the genome structure evolution at the genera level.

7) The results section are written as if it is a discussion section. For example: Line 169 and 173. Please check throughout.

8) Clymenia polyandra has already been synonymised to Citrus polyandra, according to POWO. (https://powo.science.kew.org/taxon/urn:lsid:ipni.org:names:772184-1). Please fact check.

Reviewer #2: Witharana and colleagues investigated plastome evolution in the subfamily Aurantioideae (Rutaceae), focusing on phylogenetic classification of Citrus, divergence-time estimation, structural variation at LSC-IR boundaries, and pseudogenization patterns.

This manuscript potentially presents some interesting results, but in its current form, in my opinion, it is far from being suitable for publication in a scientific journal such as PLOS One, both in terms of presentation and scientific content. I would suggest considering whether the current content might be better suited as a short communication or as a "resource paper".

The main issues are outlined below.

(1) Very limited novelty of the reported results.

Lines 65-74: I appreciate the authors' honesty in explicitly reporting these details, but this paragraph discussing the prior bioRxiv preprint and subsequent publication history reads more like a personal narrative of the authors' publication trajectory than a standard scientific introduction. Phrases detailing which analyses were "included" or "not included" in subsequent papers, and explanations of why certain findings were omitted during peer review, are better suited to a thesis preface or personal communication than to a journal article. Scientific papers should stand on their own merit and focus on the science, not the history of how the manuscript came to be.

Most importantly, the described publication history makes it clear why the current manuscript reports only some of the analyses that could have been performed with these data: this manuscript essentially presents some “leftover” analyses from the original preprint that were not developed further in the subsequent publications. This, in itself, is not necessarily a problem; however, as a result, the current manuscript does appear rather “poor” as a standalone research article, in addition to presenting problematic and unclear aspects in the way the dataset used is described, given that it derives from other studies.

Below is a detailed assessment of the novelty of the results presented in this study.

(1.1) First of all, no new raw data were generated specifically for the analysis reported in this manuscript. Indeed, the authors used sequences generated by their group which were produced in the context of previous, already published studies, and data come from publicly available sources (SRA short-read data and GenBank plastome sequences).

(1.2) Second, the phylogenetic analyses desscribed in this manuscript resolved eight major cpDNA clusters within Citrus. However, contrary to the authors’ claim, this result does not appear to be particularly novel. Indeed, a 2009 study based on PCR-RFLP and trnL-trnF sequence data from chloroplast DNA analyzed 50 Citrus accessions and also placed them into eight major clusters. The paper is: Jena, S. N., Kumar, S., & Nair, N. K. (2009). Molecular phylogeny in Indian Citrus L. (Rutaceae) inferred through PCR-RFLP and trnL-trnF sequence data of chloroplast DNA. Scientia Horticulturae, 119(4), 403-416.

As stated in the Abstract of that paper: “PCR-RFLP data showed a genetic distance ranging from 0 to 0.79 among 50 accessions of Citrus, and a cluster analysis, based on Neighbor-Joining (NJ) method, placed all the accessions in eight major clusters”.

Importantly, the authors of the present manuscript have neither compared their results with those of this study nor cited it in any way.

Similarly, another relatively recent paper, published in Nature, analyzed 58 Citrus accessions and presented a chloroplast genome phylogeny that also resolved a similar number of lineages within Citrus:

Wu, G. A., Terol, J., Ibanez, V., López-García, A., Pérez-Román, E., Borredá, C., ... & Talon, M. (2018). Genomics of the origin and evolution of Citrus. Nature, 554(7692), 311-316.

Again, the authors do not even cite this paper, despite its apparent importance in the context of Citrus phylogeny.

Overall, these omissions not only call into question the novelty of the phylogenetic result reported in the present manuscript, but also raise concerns about how the results have been discussed and contextualized within the existing literature.

(1.3) Regarding the divergence-time analysis, the authors indicated two comparable Asia-Africa splits within Aurantioideae, whereas the divergence between Australian and Asian Citrus lineages was more recent. Concerning the latter split, this is not a novel result, as it is entirely consistent with the 2018 Nature paper by Wu et al., which also provided divergence-time estimates for these splits and, as noted above, appears not to have been considered in the discussion of the present manuscript. Therefore, perhaps the only potentially novel aspect of the divergence-time analysis is the identification of the two comparable Asia-Africa splits (~5 Ma) within Aurantioideae. However, regarding the reliability of this result, see point (2).

(1.4) Results on structural variation and pseudogenization: these do indeed appear to be the only truly novel contribution of this study, but in my view, this is too limited to support a standalone paper. However, the authors themselves acknowledge: “Another represented a further development of the preprint’s structural observations by examining in detail an approximately 22 kb inversion in the psbM–trnG-UCC region and its associated short inverted repeats.” It therefore appears that at least part of the results on structural variation contained in this dataset has already been described in another paper by the authors.

(2) Methodological weakness in the divergence-time analysis.

The use of RelTime-ML in MEGA11 with a single calibration point is potentially problematic. RelTime is a relatively simple approach that may not adequately account for rate variation; moreover, although the use of a single calibration point may be appropriate, it is insufficient. A more robust approach, such as BEAST2 or MCMCTree with multiple fossil calibrations or secondary calibrations from the literature (if available), would strengthen the reliability and precision of the estimated divergence times.

(3) Insufficient description of sample and sequencing data.

Lines 94-96: this sentence don’t provide the reader with any quantitative information about the relative contributions of each data source. I suggest to specify the numbers of each subset of samples in the main text. Moreover, ensure that tables in supplementary material provides comprehensive details for each one of them, and also consider adding a brief summary table in the main text (in the main text, no tables are currently reported).

In addition, I also note that the Materials and Methods section does not provide essential information about the sequencing data used for plastome assembly. The current text states that DNA extraction and short-read sequencing were performed as described in previous studies, but this is insufficient for a standalone research article. I recommend that the authors include the following details in the present manuscript: sequencing platform and chemistry; average sequencing depth (coverage), total number of raw reads generated per sample (or the range); some quality control statistics. This holds for both newly generated data produced by the authors, and also SRA data that were downloaded and re-assembled by the authors (where available).

(4) Lack of background on pseudogenization in the Introduction. The introduction does not include any substantive background on plastid gene pseudogenization, despite this being one of the main novel contributions of the study. Currently, the term “pseudogenization” appears only in the abstract and results sections. I recommend adding a dedicated paragraph that would: define pseudogenes and their causes (in the context of plastid genomes); summarize the current understanding of pseudogenization in plant plastomes, noting that it is relatively common and often associated with functional gene transfer to the nucleus or relaxed selective constraints; mention that pseudogenization events are frequently correlated with structural rearrangements; highlight why this is particularly relevant in Aurantioideae, considering the group’s complex evolutionary history, etc.

(5) Frequent lack of appropriate literature support for the reported statements, and high rate of self citation.

There is recurrent lack of bibliographic citations supporting the statements made by the authors. Some examples include:

Lines 42-48: some literature citations are needed here to support these statements (although some of them are quite well known).

Lines 48-49: citation needed.

Lines 62-64: citation needed.

Line 127: please add the correct citation which, according to TimeTree website, should be:

S. Kumar, M. Suleski, J.E. Craig, A.E. Kasprowicz, M. Sanderford, M. Li, G. Stecher, and S.B. Hedges, 2022. TimeTree 5: An Expanded Resource for Species Divergence Times. Molecular Biology and Evolution, DOI: 10.1093/molbev/msac174.

Moreover, the TimeTree version should be specified.

Line 139: citation needed for the tool “genbank_to”; see: https://genbank-to.readthedocs.io/en/latest/changelog.html#citation.

Lines 266-269: Some literature citations are needed.

Lines 279-281: citation needed.

In addition, as many as 7 of the 37 references (i.e., references nos. 3, 4, 5, 7, 31, 32, and 34) include one or more of the co-authors of the present study among their authors, corresponding to a self-citation rate of 18.9%. Although many of these citations may be considered appropriate, this figure is rather striking; nevertheless, it could be also partly due to the low overall number of references (see above comment).

Minor comments

Lines 35-37: True, but authors should not forget introgression and cp capture in several plant groups, acting as a confounding factor. Indeed, the authors discuss these limitations at lines 51-53. I only suggest to rephrase a little bit this setence in order to avoid apparet contraddictory statements.

Line 86: Instead of “in Aurantioideae of Rutaceae”, perhaps “in the subfamily Aurantioideae of the Rutaceae” could be more appropriate and clear.

Lines 134-136: Did all the reported structural features were identified by the authors visually? Or by using which tools? These methodological details should be better specified; please also note that dedicated tools such as IRscope could be used for precise IR boundary quantification.

Lines 142-142: In pseudogene identification, do the authors validated each putative pseudogenization event by inspecting the raw read alignment in IGV, for confirming that the mutation was supported by a sufficient coverage of reads?

Lines 173-174: This is Discussion.

Lines 189: the reference to a figure is needed here.

Line 191: “more recent”: please quantify.

Line 220: “in contrast”: it is not clear to me where the two statements are opposed.

Lines 245-247: see my previous comment to lines 65-74.

Lines 260-262: This paragraph on Clymenia polyandra presents several issues. The phrase "has not formally undergone a name change" is unscientific in tone, while "it is expected to be included" is speculative. I suggest reformulating more cautiously and in an evidence-based manner, for example: "In our plastome phylogeny, Clymenia polyandra was placed within the Citrus clade, suggesting that this taxon may be a member of Citrus...".

And what about current phylogenetic information from literature about this genus? See e.g.:

Bayer, R. J., Mabberley, D. J., Morton, C., Miller, C. H., Sharma, I. K., Pfeil, B. E., ... & Sykes, S. (2009). A molecular phylogeny of the orange subfamily (Rutaceae: Aurantioideae) using nine cpDNA sequences. American Journal of Botany, 96(3), 668-685.

Oueslati, A., Ollitrault, F., Baraket, G., Salhi-Hannachi, A., Navarro, L., & Ollitrault, P. (2016). Towards a molecular taxonomic key of the Aurantioideae subfamily using chloroplastic SNP diagnostic markers of the main clades genotyped by competitive allele-specific PCR. BMC genetics, 17(1), 118.

The results described by the authors should be presented in light of prior knowledge available in the literature. Furthermore, the statement "From this perspective, the present analysis effectively represents a 56-accession study" should also be rephrased in a clearer way.

Line 276: The Isthmus of Panama is geographically distant from the Afro-Asian region, and its connection to plant dispersal between Asia and Africa is highly speculative. I recommend removing the reference to the Isthmus of Panama and focusing instead on climatic or geological changes that more directly affected the Afro-Asian region, or, as an alternative, to better detail how the closure of the Isthmus of Panama could have affected plant dispersal between Asia and Africa, with appropriate literature citations.

**********

what does this mean?). If published, this will include your full peer review and any attached files.

If you choose “no”, your identity will remain anonymous but your review may still be made public.

**Do you want your identity to be public for this peer review?**  For information about this choice, including consent withdrawal, please see our Privacy Policy

Reviewer #1: No

Reviewer #2: No

**********

[NOTE: If reviewer comments were submitted as an attachment file, they will be attached to this email and accessible via the submission site. Please log into your account, locate the manuscript record, and check for the action link "View Attachments". If this link does not appear, there are no attachment files.]

To ensure your figures meet our technical requirements, please review our figure guidelines: https://journals.plos.org/plosone/s/figures

You may also use PLOS’s free figure tool, NAAS, to help you prepare publication quality figures: https://journals.plos.org/plosone/s/figures#loc-tools-for-figure-preparation.

NAAS will assess whether your figures meet our technical requirements by comparing each figure against our figure specifications.

# Author comment: Author response to Decision Letter 1

**DOI:** 10.1371/journal.pone.0349882.r002

14 Sep 2026

Dear Dr. Siler and Reviewers,

Thank you for your careful evaluation and constructive comments on our manuscript, “Plastome evolution in Aurantioideae and plastome-based classification of Citrus.”

We have carefully addressed the comments from the Academic Editor and both reviewers and substantially revised the manuscript. Detailed point-by-point responses, including the corresponding changes and line references, are provided in the accompanying “Response to Reviewers” document.

Major revisions include reorganizing the manuscript; adding general plastome characterization and methodological details; clarifying data sources and the relationship to our previous work; repeating the divergence-time analysis using MCMCtree with multiple temporal constraints and a calibration-sensitivity analysis; and expanding comparisons with previous studies. We have also clarified the distinction between sequence-based evidence of putative pseudogenization and experimentally demonstrated functional loss.

We appreciate your thoughtful guidance and hope that the revised manuscript satisfactorily addresses your concerns.

Attachment Submitted filename: Response_to_Reviewers.docx

# Peer review: Decision Letter 1

**DOI:** 10.1371/journal.pone.0349882.r003

**Authors:** Branislav T. Šiler

21 Sep 2026

Plastome evolution in Aurantioideae and plastome-based classification of Citrus

PONE-D-26-21585R1

Dear Dr. Nagano,

We’re pleased to inform you that your manuscript has been judged scientifically suitable for publication and will be formally accepted for publication once it meets all outstanding technical requirements.

Within one week, you’ll receive an e-mail detailing the required amendments. When these have been addressed, you’ll receive a formal acceptance letter and your manuscript will be scheduled for publication.

An invoice will be generated when your article is formally accepted. Please note, if your institution has a publishing partnership with PLOS and your article meets the relevant criteria, all or part of your publication costs will be covered. Please make sure your user information is up-to-date by logging into Editorial Manager at Editorial Manager® and clicking the ‘Update My Information' link at the top of the page. For questions related to billing, please contact billing support.

If your institution or institutions have a press office, please notify them about your upcoming paper to help maximize its impact. If they’ll be preparing press materials, please inform our press team as soon as possible -- no later than 48 hours after receiving the formal acceptance. Your manuscript will remain under strict press embargo until 2 pm Eastern Time on the date of publication. For more information, please contact onepress@plos.org.

Kind regards,

Branislav T. Šiler, Ph.D.

Academic Editor

PLOS One

Additional Editor Comments (optional):

Reviewers' comments:

Reviewer's Responses to Questions

**Comments to the Author**

Reviewer #2: All comments have been addressed

**********

2. Is the manuscript technically sound, and do the data support the conclusions?

Reviewer #2: Yes

**********

3. Has the statistical analysis been performed appropriately and rigorously?

Reviewer #2: Yes

**********

4. Have the authors made all data underlying the findings in their manuscript fully available?

The PLOS Data policy

Reviewer #2: Yes

**********

5. Is the manuscript presented in an intelligible fashion and written in standard English?

Reviewer #2: Yes

**********

Reviewer #2: (No Response)

**********

what does this mean?). If published, this will include your full peer review and any attached files.

If you choose “no”, your identity will remain anonymous but your review may still be made public.

**Do you want your identity to be public for this peer review?**  For information about this choice, including consent withdrawal, please see our Privacy Policy

Reviewer #2: No

**********

# Editor report: Acceptance letter

**DOI:** 10.1371/journal.pone.0349882.r004

**Authors:** Branislav T. Šiler

PONE-D-26-21585R1

PLOS One

Dear Dr. Nagano,

I'm pleased to inform you that your manuscript has been deemed suitable for publication in PLOS One. Congratulations! Your manuscript is now being handed over to our production team.

At this stage, our production department will prepare your paper for publication. This includes ensuring the following:

* All references, tables, and figures are properly cited

* All relevant supporting information is included in the manuscript submission,

* There are no issues that prevent the paper from being properly typeset

You will receive further instructions from the production team, including instructions on how to review your proof when it is ready. Please keep in mind that we are working through a large volume of accepted articles, so please give us a few days to review your paper and let you know the next and final steps.

Lastly, if your institution or institutions have a press office, please let them know about your upcoming paper now to help maximize its impact. If they'll be preparing press materials, please inform our press team within the next 48 hours. Your manuscript will remain under strict press embargo until 2 pm Eastern Time on the date of publication. For more information, please contact onepress@plos.org.

You will receive an invoice from PLOS for your publication fee after your manuscript has reached the completed accept phase. If you receive an email requesting payment before acceptance or for any other service, this may be a phishing scheme. Learn how to identify phishing emails and protect your accounts at https://explore.plos.org/phishing.

If we can help with anything else, please email us at customercare@plos.org.

Thank you for submitting your work to PLOS One and supporting open access.

Kind regards,

PLOS One Editorial Office Staff

on behalf of

Dr. Branislav T. Šiler

Academic Editor

PLOS One
